# Clock neurons modulate seizure timing through a diurnal switch

**DOI:** 10.1038/s41467-026-77884-4

**Published:** 2026-09-30

**Authors:** Vishnu Anand Cuddapah, Cynthia T. Hsu, Chitrakshi Pant, Camilo Guevara, Jianing Yang, Samantha Killiany, Hrishit M. Shah, Mary E. Putt, Amita Sehgal

**Affiliations:** 1 Jan and Dan Duncan Neurological Research Institute, Texas Children’s Hospital, Houston, Texas USA; 2 Division of Neurology and Developmental Neuroscience, Department of Pediatrics, Baylor College of Medicine, Houston, Texas USA; 3 Howard Hughes Medical Institute, University of Pennsylvania, Philadelphia, PA USA; 4 Chronobiology and Sleep Institute, Perelman School of Medicine, University of Pennsylvania, Philadelphia, PA USA; 5 Department of Biostatistics, Epidemiology and Informatics University of Pennsylvania, Philadelphia, PA USA; 6 CHOP/Penn Intellectual and Developmental Disabilities Research Center, Philadelphia, PA USA

**Keywords:** Circadian mechanisms, Epilepsy

## Abstract

People with epilepsy experience daily oscillations in seizure risk. Using a video-tracking model to automate detection, we explored mechanisms underlying spontaneous seizures in *Drosophila* and find that flies, like people, exhibit daily oscillations in seizure risk. These rhythms are absent in flies lacking a molecular circadian clock and are independent of sleep. Thermogenetic screening and cell-specific clock perturbations reveal that the ventrolateral neurons, including “morning cells”, and dorsolateral neurons, comprising the “evening cells”, modulate diurnal oscillations in seizure risk. Pigment-dispersing factor and short neuropeptide F from morning cells increase seizure risk during the day. During this time, morning cell activity increases whole-brain activity. At night, morning cells switch to glycine signaling likely acting on evening cells to decrease seizure risk. Optogenetic inhibition of clock neurons suppresses seizure risk. Our findings reveal an unexpected role for brain clock neurons in modulating daily seizure risk.

## Abstract (web summary)

Neural mechanisms that drive diurnal oscillations in seizure susceptibility are not fully understood. Here, in this manuscript, fruit flies show the daily rhythm of seizure risks, driven by brain clock neurons and independent of sleep amount. Silencing these neurons suppressed seizures, further demonstrating the role for brain clock neurons in modulating daily seizure risk.

## Introduction

For more than 100 years, clinicians have observed daily oscillations in seizure risk in people with epilepsy. An early study of 110 people with epilepsy over 10 years revealed that seizure timing through the day is non-random^1^. Fast-forward to the modern era, and seizure diaries as well as implantable brain stimulation devices across larger cohorts of individuals continue to reveal that for people with epilepsy, seizures and interictal epileptiform activity are more likely to occur at particular times of the day^2–4^. While more than 80% of people with epilepsy exhibit diurnal oscillations in seizure risk, the exact time of day that seizure risk peaks varies from person to person and relates to underlying genetic and structural etiologies^2–5^. Across all etiologies, the peak time for seizure occurrence on average is around the early- to mid-day, though across individuals, seizure peaks can occur at any time of the day^1,4,6^. Interictal epileptiform discharges also exhibit diurnal rhythms as revealed by prolonged recordings from implanted brain stimulation devices^2^. The phase of peak seizure timing is frequently tied to underlying brain regions involved, as well as genetic etiologies^5,6^.

Identification of the molecular and cellular mechanisms that drive these diurnal oscillations in seizure susceptibility has been challenging because there are multiple processes that oscillate over a 24 h period, driven by the circadian clock or by environmental cycles. Dissecting the contribution of these different processes to seizure rhythms in people^7,8^, or even rodent models^7–10^, has been complex. Therefore, we sought to identify underlying mechanisms in *Drosophila*, as flies are an experimentally tractable model organism that exhibit well-characterized biological rhythms and lend themselves to the modeling of human seizures.

In the fly, daily behavioral rhythms are driven by ~ 240 neurons that contain a molecular clock in the brain^11^. These clock neurons are evenly divided between the two brain hemispheres and organized into 4 lateral and 3 dorsal neuronal clusters. The lateral clusters include the small and large ventrolateral neurons (LNvs), dorsolateral neurons (LNds), and lateral posterior neurons (LPNs). The LNvs express pigment-dispersing factor (PDF), which plays a critical role in synchronizing the activity of brain clock neurons. Small LNvs in particular play an important role in driving behavioral rhythms and are orthologous to the suprachiasmatic nucleus in mammals. The activity of small LNvs increases in the early morning, thereby increasing fly behavioral activity, so these cells are known as “morning cells”. In contrast, the activity of LNds peaks around early evening, so these cells are referred to as “evening cells” and known to also play a role in behavioral rhythms. The dorsal neuronal clusters include the DN1s, DN2s, and DN3s. The DN1s are downstream of small LNvs and promote activity in the early morning. In addition to clock neurons, glial subsets in the fly brain contain functional molecular clocks. Therefore, we sought to understand if flies exhibit diurnal oscillations in seizure risk, and if so, identify the driving cellular and molecular mechanisms.

## Results

### Long-term video recording of flies reveals daily seizure rhythms

**Fig. 1:**
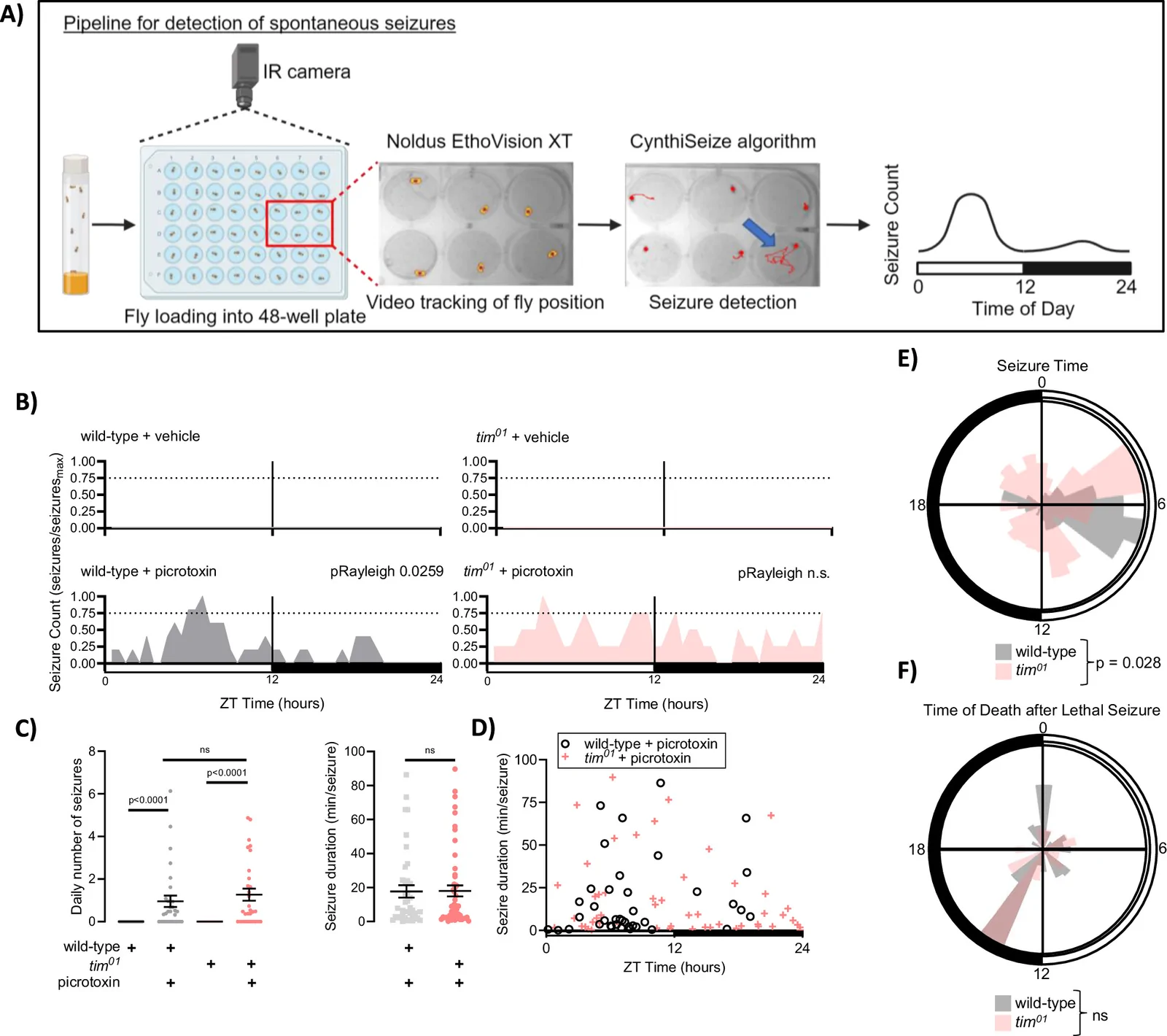
Flies exhibit diurnal seizure rhythms that are lost in *tim*^*01*^ clock-mutant flies. **A** Overview of experimental pipeline depicting tracking of fly positions in individual wells of a 48-well plate with subsequent automated detection of seizures using the CynthiSeize algorithm. This allows for measurement of seizure risk through the day. Created in BioRender. Sehgal, A. (2026) https://BioRender.com/ry6c49k**B** Seizures preferentially occur during the daytime in wild-type flies (*gray)* fed picrotoxin. In *tim*^*01*^ mutant flies (*red*) fed picrotoxin, seizures occur randomly throughout the day, even in a 12 h light:12 h dark cycle. “pRayleigh” indicates statistical significance by the Rayleigh test with permutation method. *n* = 30 flies/condition. **C** Daily number of seizures and seizure durations. Negative binomial model with Wald test and mixed effects model were used, respectively. **D** Daily distribution of seizure duration for each seizure. In (**C**, **D**), *n* = 39 seizures for ‘wild-type + picrotoxin’ and *n* = 51 seizures for ‘*tim*^*01 *^+ picrotoxin’. **E, F**, Rose plots depicting time of seizure occurrence (**E**) and time of death due to lethal seizures (**F**). The radius of each rose plot bar indicates the normalized count of seizure episodes occurring during the given time bin. *n* = 28-29 lethal seizures/condition. Watson’s two-sample test of homogeneity was used to detect differences in seizure timing between groups. Data are presented as mean values ± SEM. Source data are provided as a Source Data file.

We first asked whether seizures in *Drosophila* exhibit diurnal rhythms as seen in people and mammalian models^2,4,8,12^. Flies were loaded into individual wells of 48-well plates and continuously video recorded for 96 hours (Fig. 1A). Fly position over time was measured using Noldus EthoVision XT. Using this position information and frame-to-frame pixel changes, tonic-clonic seizures were identified as previously described^13^. Wild-type Canton-S flies did not exhibit seizures, but when fed picrotoxin, a GABA_A_-receptor antagonist, seizures were induced. We refer to the seizures induced by picrotoxin feeding as “spontaneous” seizures, as no acute stimulus is delivered to precipitate a seizure (in contrast to “bang-sensitive” or temperature-sensitive seizures described below). Interestingly, when measuring these seizures across a 24-hour cycle of 12 h of light and 12 h of dark (12 h light:12 h dark), seizures were more likely to occur during the day (Fig. 1B). We employed a permutation-based Rayleigh test to determine whether the diurnal patterns seen in seizure occurrence differed statistically from a uniform, or equivalently random, distribution. Controlling for individual flies having multiple seizures^14^, we found that seizure distribution across a 24 h day was non-uniform (Fig. 1B–D).

We hypothesized this diurnal pattern might be driven by the circadian molecular clock. When *timeless* (*tim*^*01*^) mutant flies, which lack a functional molecular clock but still exhibit diurnal patterns of activity in a light:dark cycle^15^, were fed picrotoxin, seizures were randomly distributed throughout the day and did not show evidence of differing from a uniform distribution (Fig. 1B–D; pRayleigh > 0.05). While there were no differences in the number or duration of seizures (Fig. 1C, D), the timing of seizures through the day differed significantly as measured by Watson’s two-sample test of homogeneity (Fig. 1E). These differences in seizure timing were present even though *tim*^*01*^ flies exhibit diurnal behavioral rhythms in a 12 h light:12 h dark cycle^15^. While the timing of all seizures appeared random in *tim*^*01*^ mutants, lethal seizures were most likely in the early evening as seen in wild-type flies (Fig. 1F, *lower panel*). This indicates that diurnal rhythms related to lethal seizures may be independent of a functional molecular clock. Picrotoxin-treated wild-type and *tim*^*01*^ flies slept at similar levels (Supplementary Fig. 1A), showing that the time-of-day effects were not due to increased sleep need^13^. Survival after picrotoxin treatment was similar in wild-type and *tim*^*01*^ flies (Supplementary Fig. 1B). Also, differences are not due to altered feeding as *tim*^*01*^ mutants and other clock mutants show rhythmic feeding in a light:dark cycle^16–18^. We find too that food intake is similar in wild-type flies and *tim*^*01*^ mutants at two different times of day (Supplementary Fig. 3A). Importantly, shifting the timing of picrotoxin exposure by 12 h to the nighttime continued to lead to daytime-predominant seizures (Supplementary Fig. 1C).

These daytime predominant seizures persisted in constant darkness, though, as seen for other circadian processes^17^, the rhythm is dampened (Supplementary Fig. 2A, B). In constant light, diurnal differences in seizures were dampened, and there was a shift in phase to a nighttime peak in seizures. Because constant light disrupts the clock more slowly^19^ and less completely than a *tim*^*01*^ null mutant, residual circadian organization of seizures is not surprising. In addition, flies in constant light exposed to picrotoxin sleep less (Supplementary Fig. 2E), which independently increases seizure risk^13^. Surprisingly in constant light, we detected a seizure in a wild-type fly and a six-fold increase in picrotoxin-induced seizures, reflecting previously-described toxicity of constant light exposure^20^ (Supplementary Fig. 2C, D and Supplementary Video 2). After confirming picrotoxin-induced seizures exhibit diurnal rhythms that are dependent on the molecular clock, we sought to identify the cell type(s) driving rhythms.

### Diurnal rhythms in bang-sensitive seizures depend on circadian clock neurons

**Fig. 2:**
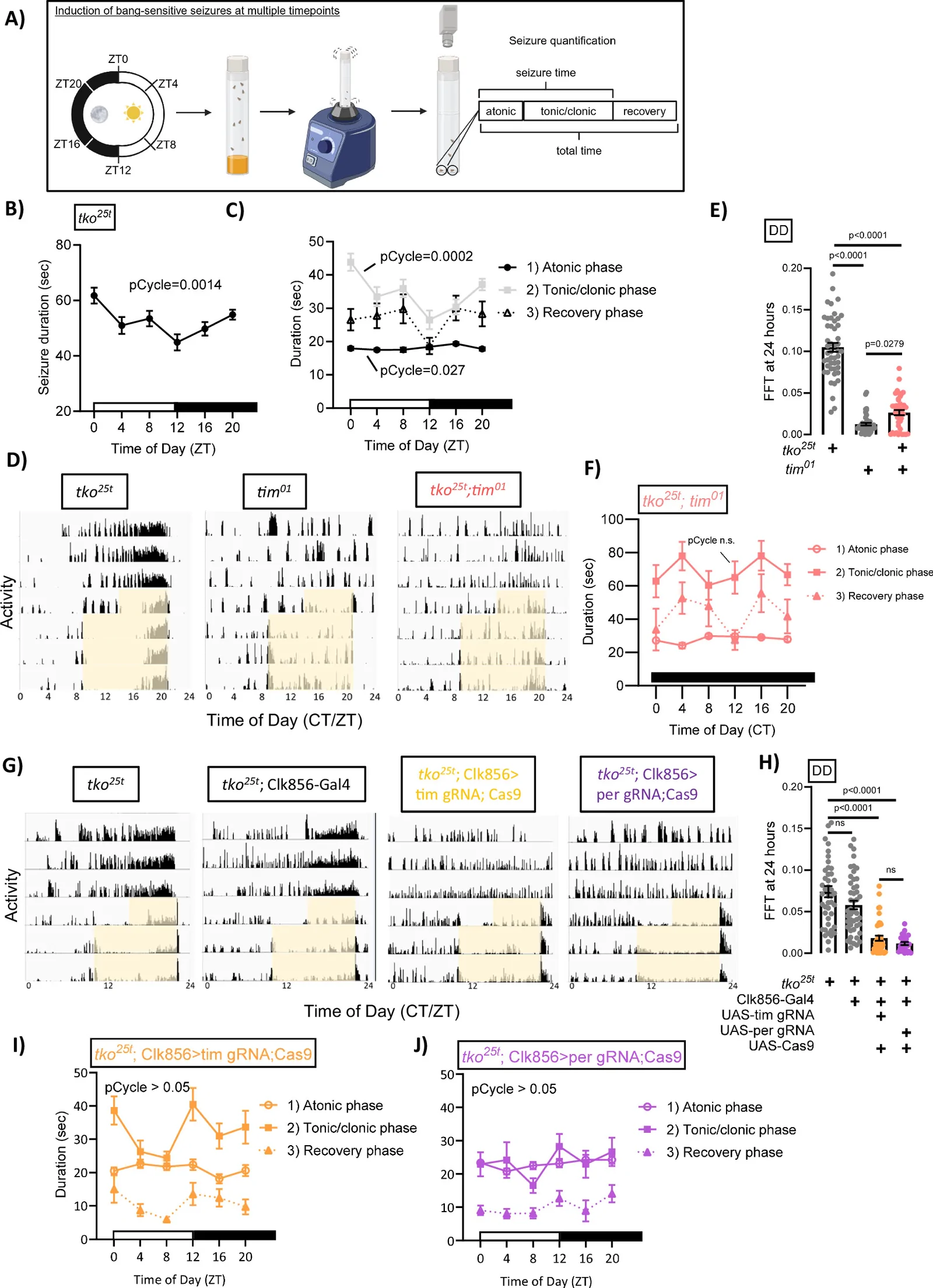
Induced seizures exhibit diurnal rhythms in severity that are dependent on the molecular clock. **A** Experimental overview showing 6 timepoints at which bang-sensitive seizures were induced using a table-top vortexor. Seizures were then video recorded to quantify the atonic, tonic/clonic, and recovery phases. Created in BioRender. Sehgal, A. (2026) https://BioRender.com/kmpo9hx**B**, **C** Duration of seizures or seizure components in *tko*^25t^ flies across the day. At ZT0 *n* = 38 seizures, at ZT4 *n* = 37 seizures, at ZT8 *n* = 37 seizures, at ZT12 *n* = 32 seizures, at ZT16 *n* = 41 seizures, at ZT20 *n* = 37 seizures. JTK cycle, a test for rhythmicity, was used the and *p*-value is provided in the Figure as “pCycle”. **D**, **G** Representative activity records in which vertical lines indicate activity. The rows represent successive days over which activity was monitored. Yellow shading indicates light for a 12-hour light:12 h dark period. **E**, **H** Fast Fourier Transform (FFT) calculations. For FFT analyses, *n* = 48 flies for ‘*tko*^25t^’, *n* = 47 flies for ‘*tim*^*01*^’, *n* = 41 flies for ‘*tko*^25t^*/tim*^*01*^’, *n* = 44 flies for ‘*tko*^25t^’, *n* = 47 for ‘*tko*^25t^;Clk856-Gal4’, *n* = 43 for ‘*tko*^25t^;Clk856-Gal4 > UAS-*tim* gRNA, UAS-Cas9’, *n* = 27 for ‘*tko*^25t^;Clk856-Gal4 > UAS-*per* gRNA, UAS-Cas9’. One-way ANOVA with Tukey’s multiple comparisons test was used. **F** Seizure duration in *tko*^25t^;*tim*^*01*^ double-mutants show no rhythms through the day in constant darkness, as indicated by the *black* bar. At CT0 *n* = 12 seizures, at CT4 *n* = 12 seizures, at CT8 *n* = 6 seizures, at CT12 *n* = 10 seizures, at CT16 *n* = 14 seizures, at CT20 n = 15 seizures. JTK cycle was used to test rhythmicity, and the *p*-value is provided in the Figure as pCycle. **I**, **J** Rhythms of seizure duration are suppressed after perturbation of the molecular clock. For ‘*tko*^25t^;Clk856 > *tim* gRNA; Cas9’, at ZT0 *n* = 20 seizures, at ZT4 *n* = 21 seizures, at ZT8 *n* = 24 seizures, at ZT12 *n* = 22 seizures, at ZT16 *n* = 19 seizures, at ZT20 *n* = 22 seizures. For ‘*tko*^25t^;Clk856 > *per* gRNA; Cas9’, at ZT0 *n* = 19 seizures, at ZT4 *n* = 24 seizures, at ZT8 *n* = 18 seizures, at ZT12 *n* = 18 seizures, at ZT16 *n* = 12 seizures, at ZT20 *n* = 14 seizures. JTK cycle was used to test rhythmicity, and the *p*-value is provided in the Figure as pCycle. Data are presented as mean values ± SEM. Source data are provided as a Source Data file.

To screen for the cells driving diurnal rhythms in seizures and assess other models of epilepsy, we opted for a well-characterized model of epilepsy in which “bang-sensitive” seizures are acutely induced. “Bang-sensitive” seizures can be induced in *tko*^25t^ flies after exposure to a strong mechanical stimulus^21^, such as shaking with a tabletop vortex mixer (Fig. 2A). These induced seizures are stereotyped and follow a sequence of behavior that includes (a) an atonic phase manifesting as loss of tone, (b) a tonic-clonic convulsive phase, and (c) a post-ictal recovery phase (Fig. 2A). This identification of seizure phases was not possible using our video tracking-based approach in Fig. 1A. Seizures were induced at 6 timepoints through the day (ZT0, ZT4, ZT8, ZT12, ZT16, and ZT20 where “ZT” is zeitgeber time) and video recorded to allow for manual quantification of each phase of the seizure (Fig. 2A and Supplementary Video 1). The duration of seizures oscillated over 24 h as measured by JTK Cycle analysis, an algorithm used to detect rhythmicity, with peak seizure times occurring around ZT0 (Fig. 2B). This oscillation in seizure duration was predominantly driven by a strong cycle in tonic-clonic durations, and to a lesser extent, by a weak cycle in the atonic phase (Fig. 2C). While a seizure rhythm persisted in complete darkness, it was dampened (Supplementary Fig. 1E, F), consistent with other clock-dependent processes^17^. No significant differences in the likelihood of seizure induction were noted in *tko*^25t^ flies through the day (Supplementary Fig. 1D). We also tested two clinically relevant models of epilepsy: *para*^*GEFS+*^ and *para*^*DS*^ ^22,23^. These flies contain knock-in mutations in *paralytic*, the fly ortholog of the human *SCN1A*, which is implicated in Genetic Epilepsy with Febrile Seizures Plus (GEFS + ) and Dravet Syndrome. Incubation of flies at 30°C revealed a diurnal seizure rhythm with a peak occurring at the day-to-night transition (Supplementary Fig. 4). Thus, as seen in people with epilepsy, rhythms in seizure severity are widespread, occurring across the 3 genetic models and 1 pharmacological model we studied here, with the phase of the seizure peak being etiology-specific.

To assess if the rhythm in severity of bang-sensitive seizures was clock-dependent, we crossed *tko*^25t^ flies to clock-deficient *tim*^*01*^ flies and confirmed a dampening/loss of behavioral rhythms (Fig. 2D, E). *tko*^25t^*; tim*^*01*^ flies exhibited no 24-hour rhythms in seizure duration with the emergence of a possible ultradian rhythm (Fig. 2F and Supplementary Fig. 1H). Again, the likelihood of seizure induction across groups did not change across the day (Supplementary Fig. 1G). Both neurons and glia in the fly brain contain molecular clocks, so we used CRISPR/Cas9-mediated disruption of core clock components to find which cell(s) mediated seizure rhythms. Guide RNA (gRNA) targeting either *timeless* or *period* and Cas9^24^ were expressed under the control of the Clk856-Gal4 driver^25^ to target most of the 240 clock neurons. As expected, compared to *tko*^25t^ and *tko*^25t^; *Clk856*-Gal4 controls, expression of gRNA targeting either *timeless* or *period* led to a loss of behavioral rhythms (Fig. 2G, H). In addition, disruption of *timeless* or *period* expression led to a loss in rhythms of seizure duration (Fig. 2I, J). In contrast, driving *timeless* gRNA and Cas9 in glial cells did not affect seizure rhythms (Supplementary Fig. 1I, J). These results indicate that a functional molecular clock in brain clock neurons drives daily oscillations in seizure severity.

**Fig. 3:**
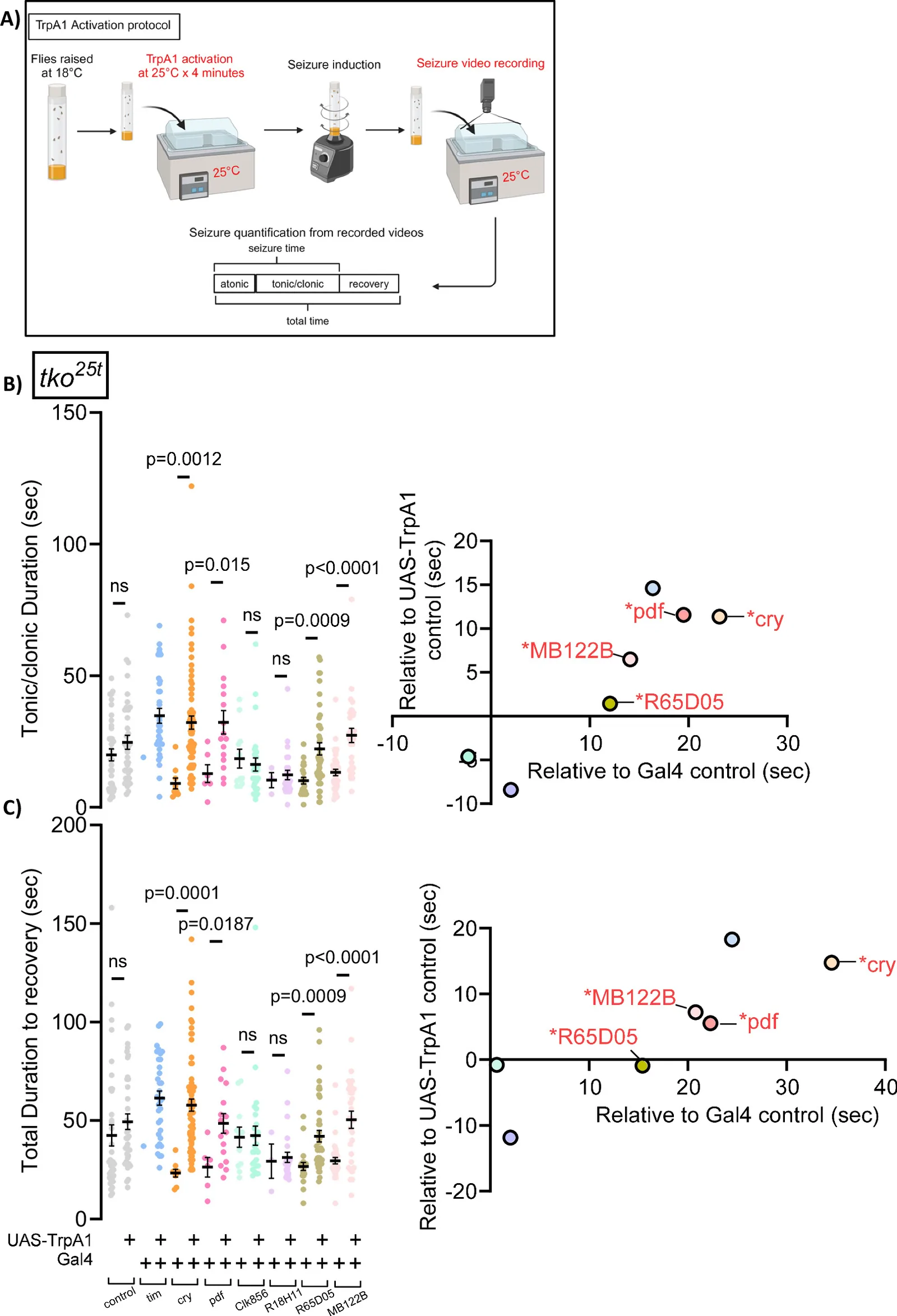
A Gal4 screen of clock neurons reveals subtypes that acutely worsen induced seizures. **A** Schematic depicting the TrpA1 activation protocol in which flies were raised at 18 °C then between ZT0-4 transferred to a 25 °C water bath for 4 min. Seizures were then induced, and flies were returned to the water bath for video recording and subsequent seizure quantification. Created in BioRender. Sehgal, A. (2026) https://BioRender.com/351daou**B**, **C** *tko*^25t^ flies exhibited prolonged tonic/clonic times and increased total duration to recovery when TrpA1 expression was driven by the *cry*-Gal4, *Pdf*-Gal4, R65D05-Gal4, and MB122B-Gal4 drivers. In the *xy* plots, the *x* is calculated by subtracting the Gal4 genetic control average seizure duration from the experimental group. The *y* is calculated by subtracting the UAS genetic control average seizure duration from the experimental group. Groups that are significantly different from the corresponding genetic control are labeled in *red* with an asterisk. The number flies assessed for each condition is given in *()*: *tko*^25t^ (37), TrpA1 (37), tim (1), tim > TrpA1 (33), cry (9), cry > TrpA1 (67), Clk856 (11), Clk856 > TrpA1 (27), pdf (6), pdf > TrpA1 (16), R65D05 (21), R65D05 > TrpA1 (41), R18H11 (3), R18H11 > TrpA1 (25), MB122B (37), and MB122B > TrpA1 (29). For unknown reasons, in *tim*-Gal4;*tko*^25t^ flies, only 1 out of 96 flies tested exhibited a seizure so significance testing is not performed for this group. Two-group two-tailed *t* test. Data are presented as mean values ± SEM. Source data are provided as a Source Data file.

Given that a functional molecular clock in clock neurons was necessary for diurnal changes in seizure severity, we next sought to identify the relevant cellular subpopulation. In *tko*^25t^ bang-sensitive mutants, we drove the expression of TrpA1, a temperature-sensitive cation channel, that allows for depolarization of cells of interest when the temperature is raised. UAS-TrpA1 was expressed under the control of each of 7 Gal4 drivers (*tim*-Gal4, *cry*-Gal4, *Pdf*-Gal4, *Clk856*-Gal4, R18H11-Gal4, R65D05-Gal4, and MB122B-Gal4) to identify the subsets of clock neurons capable of modulating seizure severity. Flies were raised at 18 °C then transferred to 25 °C to induce opening of TrpA1 (Fig. 3A). This activation protocol was developed because it induces opening of TrpA1^26^, but also avoids known direct temperature-dependent effects on seizure severity observed at higher temperatures^27^. Seizures were then induced between ZT0-4, and seizure likelihood and duration were quantified through video recording. We found that acute hyperactivation of cells expressing *cry*-Gal4, which include LNvs, some LNds, some DN1s, but not DN2s and DN3s^28^, led to prolonged tonic-clonic times (Fig. 3B) and overall time to recovery (Fig. 3C and Supplementary Fig. 5D). TrpA1 expression with *Pdf*-Gal4, which targets LNvs including the *Pdf*+ small LNvs known as “morning cells”, and MB122B-Gal4, which targets “evening cells” comprised of the LNds and *Pdf*- 5^th^ sLNv, both lengthened seizure times (Fig. 3 and Supplementary Fig. 5C). R65D05-Gal4, which targets the LPN neurons, also led to prolonged seizure times (Fig. 3 and Supplementary Fig. 5). Direct activation of DN1s using R18H11-Gal4 did not significantly change seizure durations (Fig. 3B, C). Interestingly, acute activation of *Clk856*-Gal4, which expresses in most clock neurons, did not change seizure durations (Fig. 3B, C), perhaps due to antagonistic effects of subsets of clock neurons. While UAS-TrpA1 expression alone without a genetic driver did increase the likelihood of seizure induction in genetic controls (Supplementary Fig. 5A), consistent with leaky transgene expression, no significant differences were noted in the tonic/clonic time or total duration to recovery (Fig. 3B, C). Through these thermogenetic experiments, we found that acute activation of 3 clock subpopulations, including morning cells, evening cells, and LPNs, is capable of changing seizure severity.

### Clock function in morning cells and evening cells controls seizure rhythms and risk

**Fig. 4:**
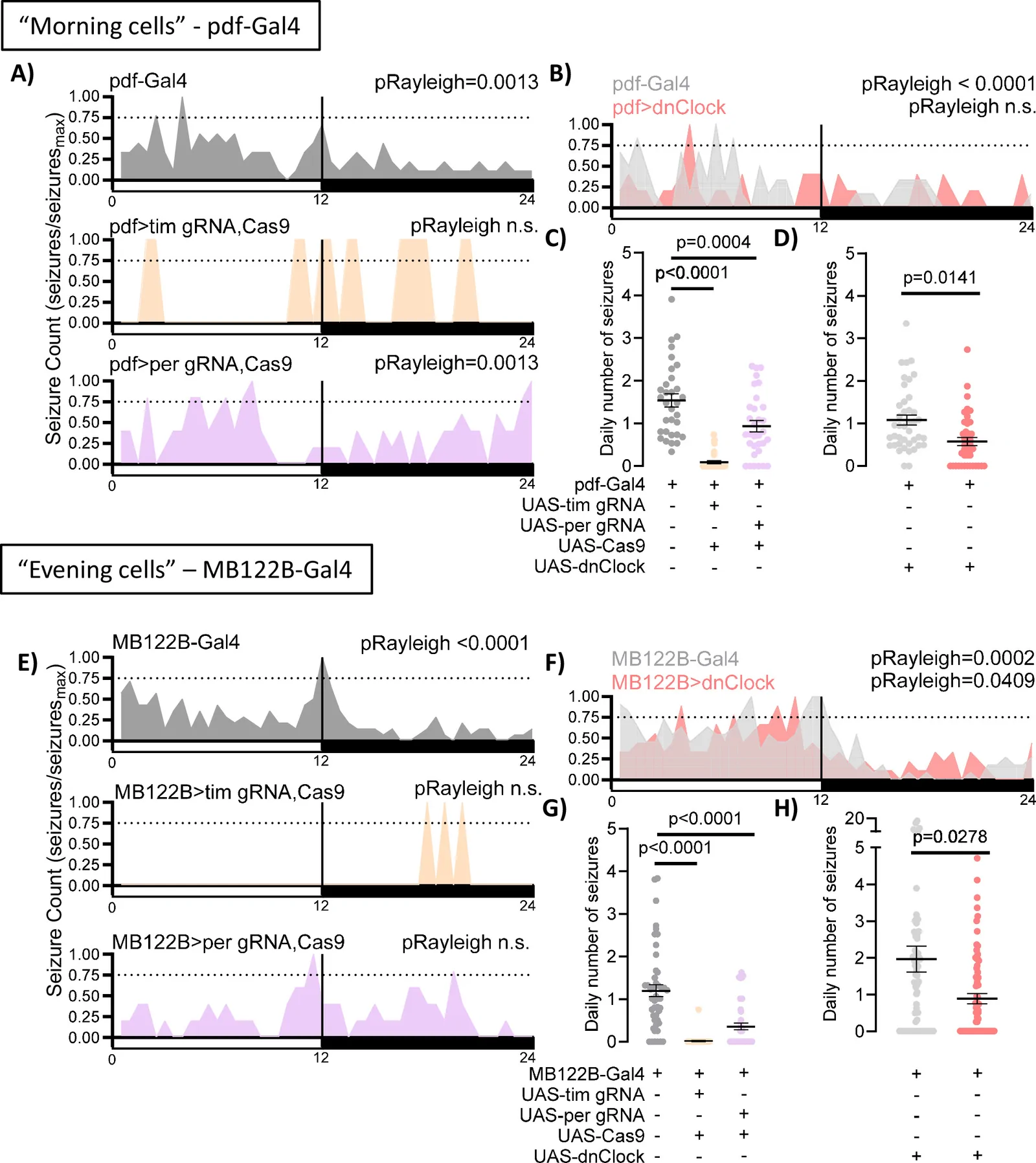
Molecular clock disruption in morning cells and evening cells changes seizure timing and likelihood. **A**, **B**, **E**, **F** Disruption of the molecular clock in *Pdf*+ morning cells, and also evening cells, leads to a relative increase in nighttime seizures. “pRayleigh” indicates results of the Rayleigh test. **C**, **D**, **G**, **H** Molecular clock disruption in morning cells and evening cells leads to seizure suppression. The number flies assessed for each condition is given in *()*: pdf-Gal4 (32), pdf > tim gRNA,Cas9 (32), pdf > per gRNA,Cas9 (32), pdf-Gal4 (41), pdf > dnClock (41), MB122B-Gal4 (47), MB122B > tim gRNA,Cas9 (47), MB122B > per gRNA,Cas9 (47), MB122B-Gal4 (72), and MB122B > dnClock (72). Negative binomial model with Wald test was used. Data are presented as mean values ± SEM. Source data are provided as a Source Data file.

As the activity of morning cells, evening cells, and LPNs can modulate seizure severity, we next sought to understand if molecular clock function was necessary in these clusters to produce daily rhythms in seizure likelihood. As this could not be assayed in the induced seizure models, we returned to our chronic video tracking platform for the detection of spontaneous seizures in picrotoxin-treated flies. Flies were again fed picrotoxin, and then seizure timing and duration were quantified using the video tracking platform. While genetic control flies continued to have daytime-predominant seizures, the nighttime seizures became more frequent when gRNA targeting either *timeless* or *period* with Cas9 was expressed in morning cells using the *Pdf*-Gal4 driver (Fig. 4A). Unexpectedly, not only did targeting of the molecular clock change seizure timing, but it also suppressed seizures. Expression of *timeless* gRNA in morning cells blocked daytime seizures, while expression of *period* gRNA led to a decrease in the total number of seizures with a relative increase in nighttime seizures (Fig. 4A, C). Similarly, targeting of the positive arm of the molecular clock with dnClock, a dominant-negative allele of Clock, led to a loss of seizure rhythms (pRayleigh n.s.), with a decrease in daily numbers of seizures (Fig. 4B, D). To distinguish the effects of small versus large LNvs, which are both targeted by the *Pdf*-Gal4 driver, we drove gRNA targeting *period* with Cas9 using the GMR_SS00681-Gal4 driver to selectively target small LNvs. Disruption of clock function in small LNvs led to the emergence of nighttime seizure peaks without a change in daily seizure number (Supplementary Fig. 6). We note that among control genotypes, daytime seizures varied from midday (Figs. 1B, 4A) to early and late in the day (Supplementary Fig. 6A). Changes in seizure severity did not correlate with changes in sleep amount (Supplementary Fig. 7A–D) and given the maintenance of control and experimental flies in a 12-h light:12 h dark cycle, sleep continued to be consolidated at night (Supplementary Fig. 7). These experiments demonstrated that a functional molecular clock in *Pdf*+ morning cells promotes diurnal seizure rhythms.

We then asked if a molecular clock is necessary in evening cells for daily seizure rhythms. While genetic controls again demonstrated increased seizure risk during the day, driving *timeless* or *period* gRNA in evening cells using the split-Gal4 driver MB122B-Gal4 led to a loss of daytime seizures (Fig. 4E). As seen in morning cells, there was strong suppression of the total numbers of seizures when the molecular clock was disrupted in evening cells (Fig. 4G). However, unlike the effects seen in morning cells, driving dnClock in evening cells did not blunt daytime seizures (Fig. 4F), though it did decrease the total number of seizures (Fig. 4H). This might indicate that while a functional molecular clock in morning cells is necessary for seizure rhythms, clock function in evening cells strengthens seizure rhythms, but may be dispensable. These effects could not be explained by consistent effects of daytime or nighttime sleep (Supplementary Fig. 7E–H). In addition, perturbation of clock function in the LPNs using R65D05-Gal4 to drive *timeless* or *period* gRNA did not significantly suppress daytime seizures or change sleep amount (Supplementary Fig. 8). Therefore, molecular clock function in morning cells, with some contribution from evening cells, increases seizure risk during the day.

### Optogenetic control of morning and evening cells modulates seizure risk

**Fig. 5:**
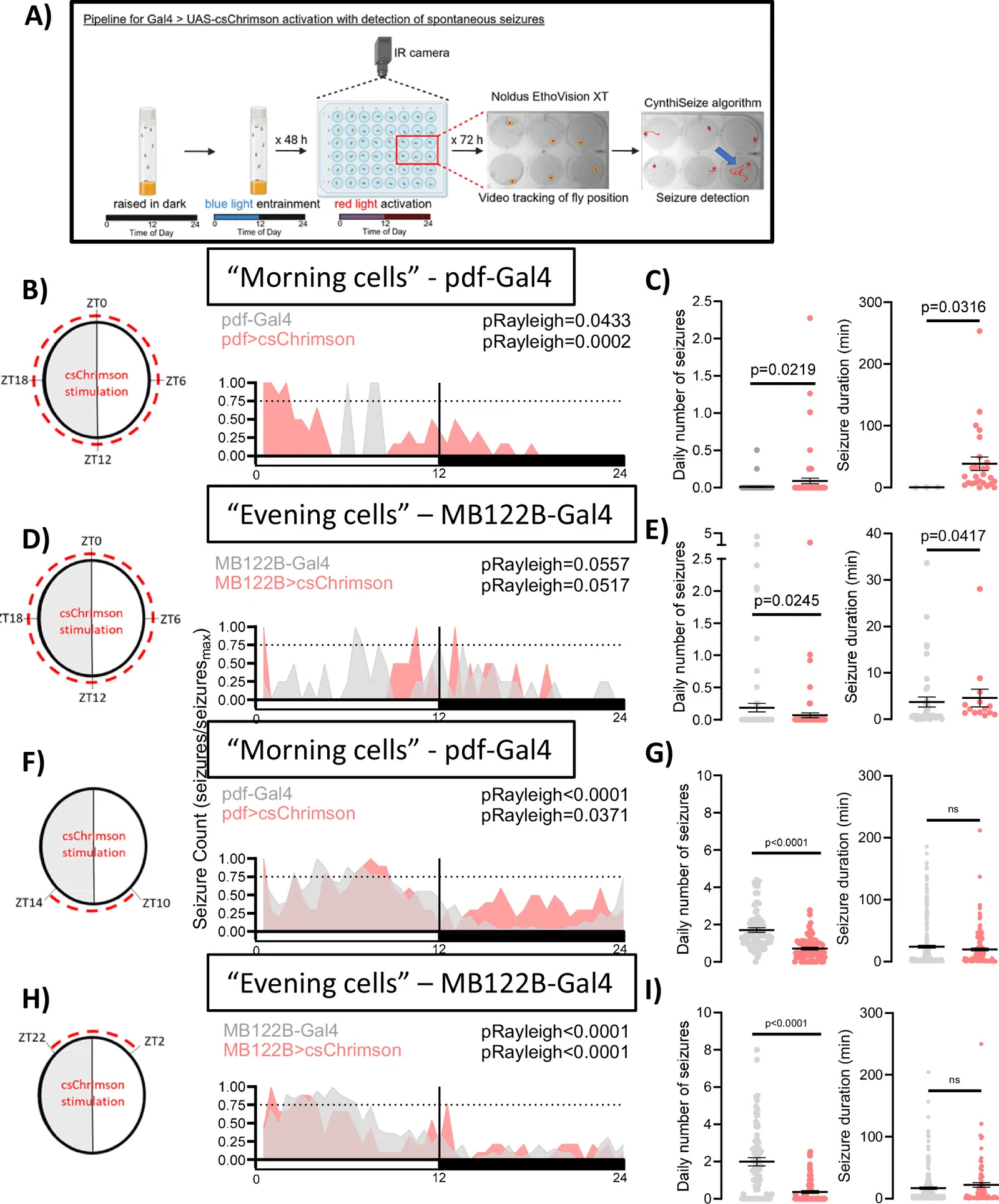
Direct optogenetic activation of morning cells and evening cells changes seizure likelihood. **A** Schematic depicting experimental protocol: flies are raised in the dark then entrained in blue light for 48 h. Flies are then placed into 48-well plates for csChrimson stimulation with red light for 72 h. Red light was switched on for 5 min and off for 25 min at the times depicted in (**B**, **D**, **F**, **H***)*. Noldus EthoVision XT was used for video tracking, and seizures were detected using the CynthiSeize algorithm. Created in BioRender. Sehgal, A. (2026) https://BioRender.com/wdglfdk**B**, **D**, **F**, **H** Clocks depict times of the day that red light was used to activate csChrimson. Activation of morning cells throughout the day increased the number of daytime seizures (**B**), while activation of evening cells throughout the day qualitatively suppressed daytime seizures (**D**). The lack of detection of a rhythm in *d* for MB122B-Gal4 controls is likely related to a reduced power to detect a statistical difference secondary to decreased seizure numbers in a lower concentration of picrotoxin. Activation of morning cells (**F**) or evening cells (**H**) during times when they are not typically active led to a suppression of daytime seizures. For (**B**, **C***)* and (**F**, **I***)*, *n* = 72 flies/condition. For (**D**, **E***)*, *n* = 94 flies/condition. “pRayleigh” indicates results of the Rayleigh test. **C**, **E**, **G**, **I** Activation of morning cells throughout the day leads to an increase in seizure number and duration. Activation of morning cells when they are not typically active (ZT10-14) or evening cells leads to a reduced number of seizures. For low picrotoxin conditions (**B**–**E**), *n* = 3 seizures for ‘pdf-Gal4’, *n* = 26 seizures for ‘pdf > csChrimson’, *n* = 42 seizures for ‘MB122B-Gal4’, and *n *= 14 seizures for ‘MB122B > csChrimson’. For high picrotoxin conditions (**F**–**I**), *n* = 316 seizures for ‘pdf-Gal4’, *n* = 150 seizures for ‘pdf > csChrimson’, *n* = 241 seizures for ‘MB122B-Gal4’, and *n* = 90 seizures for ‘MB122B > csChrimson’. A negative binomial model with a Wald test was used for seizure number. A mixed effects model was used for seizure duration. Data are presented as mean values ± SEM. Source data are provided as a Source Data file.

TrpA1 activation suggested that the activity of specific clock neurons needs to be modulated for rhythms of seizure duration, but did not indicate how this activity relates to seizure occurrence. Given that cycles of the molecular clock positively correlate with cycles of clock neuron activity^29–31^, we sought to test if clock neuron activity is directly associated with seizure risk. Based upon our above findings involving the clock machinery, we hypothesized that the activity of morning cells and evening cells underlie seizure rhythms. In each of these clusters, we expressed csChrimson, a channelrhodopsin that is activated by red light^32^; this tool has the added benefit of causing minimal behavioral artifacts while also penetrating the cuticle, allowing for seizure detection in freely moving flies. Flies were entrained for 48-hours to a 12-hour light:12-hour dark cycle, using blue light, which effectively entrains flies while limiting excitation of csChrimson through the cuticle^33–36^. Subsequently, seizures were detected via video recording for 72 h under an IR camera (Fig. 5A). Red light stimulation occurred around the clock, with lights on for 5 minutes and off for 25 min to decrease adaptation from constant light exposure as seen in other channelrhodopsins^26^.

Activation of csChrimson in morning cells using the *Pdf*-Gal4 driver led to an increase in seizure number and duration (Fig. 5B, C). We noted that a high seizure density was associated with lethality (Supplementary Fig. 1B), so we reduced the concentration of picrotoxin by 10-fold to avoid lethality and improve our ability to see increases in seizure numbers. Despite stimulation throughout the day, seizures clustered in the early morning (Fig. 5B), when morning cells are known to have more synaptic connections with downstream partners^37^. We repeated the same stimulation protocol after driving csChrimson expression in evening cells using the MB122B-Gal4 driver, using the higher picrotoxin concentration, and conversely found a reduction in seizure number (Fig. 5D). In genetic controls, although not strictly statistically significant (Fig. 5D, *p* = 0.056), seizures appeared to cluster in the morning. When morning cells were hyperactivated with the higher picrotoxin concentration from ZT10-14, a time when these cells are typically inactive^31,38^, seizure rhythms were dampened, though still present (Fig. 5F), with a decrease in seizure risk through the day (Fig. 5G). Evening cells behaved similarly: activation of evening cells from ZT22-2, when they are typically inactive, led to a decrease in seizure risk without affecting seizure rhythms (Fig. 5H, I). Together, these results indicate that (1) morning and evening cell activity can change seizure risk, and (2) the effect of morning cell activity on seizure risk is time-of-day dependent.

**Fig. 6:**
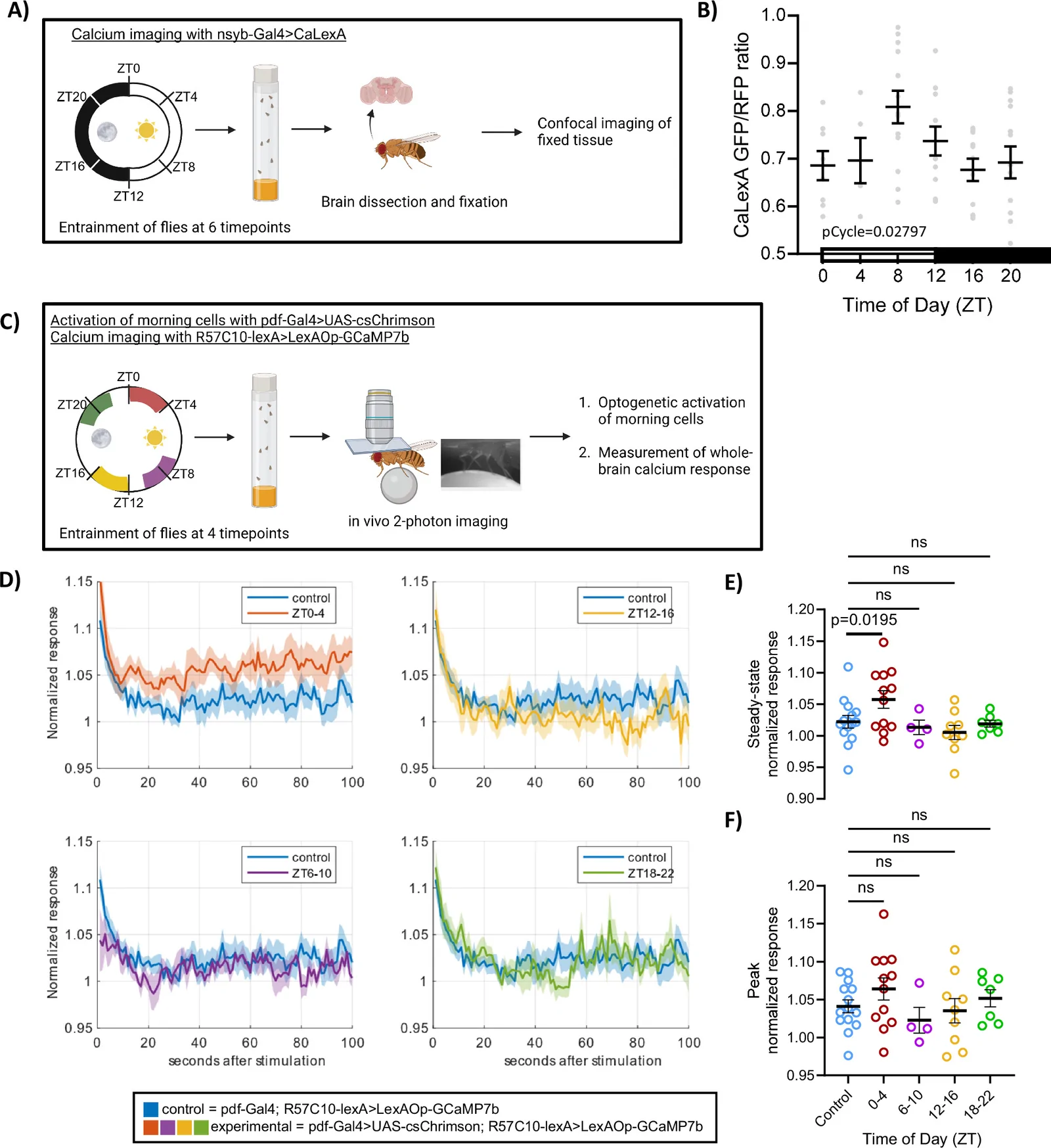
Morning cells increase global brain activity during the day. **A** CaLexA was driven pan-neuronally using nsyb-Gal4. Brains from male adult flies were dissected and fixed at 6 timepoints through the day. Created in BioRender. Sehgal, A. (2026) https://BioRender.com/7245gli**B** CaLexA signal cycles through the day with a peak during the daytime. At ZT0 *n* = 8 brains, at ZT4 *n* = 5 brains, at ZT8 *n* = 13 brains, at ZT12 *n* = 12 brains, at ZT16 *n* = 11 brains, and at ZT20 *n* = 13 brains. MetaCycle (pCycle) was used to test for rhythmicity. **C** pdf-Gal4 was used to drive UAS-csChrimson expression in morning cells. In addition, R57C10-lexA was used to drive LexAOp-GCaMP7b pan-neuronally. Adult female flies were imaged in a “fly-on-the-ball” configuration at 4 timepoints through the day. csChrimson was stimulated with whole-field red light for 100 ms. GCaMP7b response was measured throughout the brain. Created in BioRender. Sehgal, A. (2026) https://BioRender.com/nkvv7wo**D** GCaMP7b signal increased after morning cell stimulation between ZT0-4, but not at other times. **E** Steady-state (20–100 seconds) or (**F**), acute (0–20 seconds) calcium responses were recorded after stimulation. For Control *n* = 14 brains, at ZT0-4 *n* = 12 brains, at ZT6-10 *n *= 4 brains, at ZT12-16 *n* = 9 brains, and at ZT18-22 *n* = 7 brains. One-way ANOVA with uncorrected Fisher’s LSD test was used. Data are presented as mean values ± SEM. Source data are provided as a Source Data file.

Seizures are manifestations of hypersynchronous, high-frequency brain activity. This led us to ask if morning cells might increase seizure risk by controlling cycles in whole-brain activity. We drove expression of CaLexA, a genetically-encoded fluorescent calcium sensor^39^, pan-neuronally using *nsyb*-Gal4 and measured calcium signal as a surrogate measure of brain activity at 6 timepoints through the day (Fig. 6A). We found that intracellular calcium concentration as measured by CaLexA oscillates through the day with a peak during the daytime (Fig. 6B). Because the kinetics of CaLexA are slow due to its transcription-dependence and need for GFP molecules to diffuse into distal processes^39^, the true biological peak of calcium is likely hours earlier than ZT8, closer to the early- to mid-day. To further understand if this peak in brain activity during the day is being driven by morning cell activity, we again drove expression of csChrimson in morning cells while performing in vivo 2-photon imaging at 4 timepoints through the day (Fig. 6C). Whole-brain calcium responses were measured by GCaMP7b signal expressed pan-neuronally using the R57C10-lexA driver (Fig. 6C). Stimulation of csChrimson with red light for 100 ms led to an increase in intracellular calcium throughout the brain, but only when the stimulation was delivered during the day between ZT0-4, as compared to a genetic control (Fig. 6D). This increase in intracellular calcium manifested as an increase in steady-state calcium levels (Fig. 6E) without a significant increase in the peak calcium response (Fig. 6F). We also quantified the background frequency before and after stimulation at all timepoints but did not detect significant differences. Together, these data indicate that morning cell activity globally increases brain activity and seizure risk during the day.

To inhibit morning and evening cells, we expressed GtACR1, a channelrhodopsin permeable to anions and activated by green light^40^, using the *Pdf*-Gal4 and MB122B drivers, respectively (Supplementary Fig. 9A). Exposure to pulses of visible green light around the clock led to sleep fragmentation. As anticipated, inhibition of morning cells around the clock led to a dampening of seizure rhythms (Supplementary Fig. 9B) and a decrease in total seizure likelihood relative to genetic controls (Supplementary Fig. 9C, D). Similar effects were seen with inhibition of evening cells (Supplementary Fig. 9E–G). Thus, these data support the hypothesis that the molecular clock in morning and evening cells modulates neuronal activity to drive daily seizure rhythms.

### PDF and sNPF drive daytime seizures, while glycine suppresses nighttime seizures

**Fig. 7:**
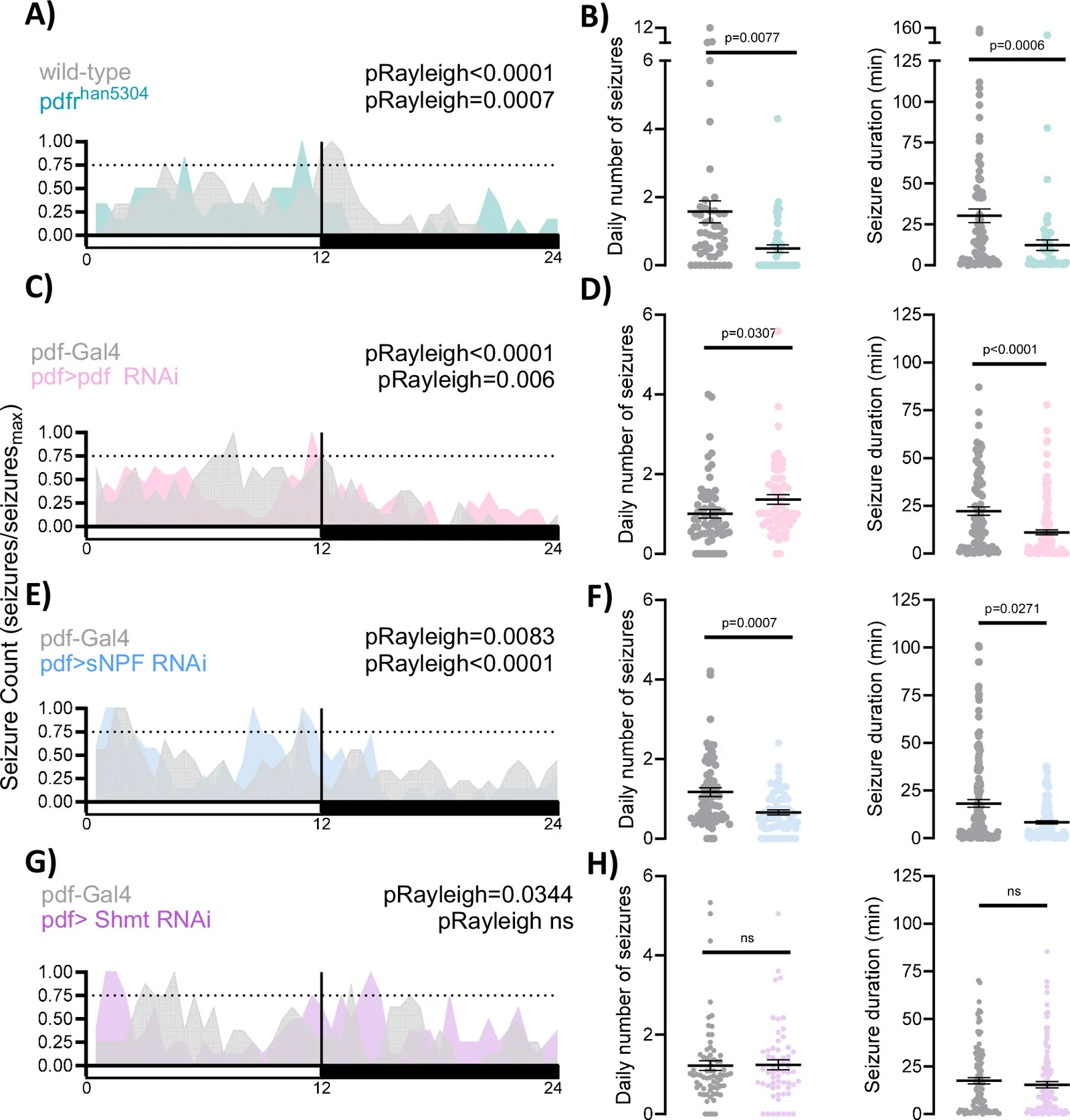
PDF and sNPF from morning cells promote daytime seizures, while glycine (synthesized by Shmt) suppresses nighttime seizures. **A**, **C**, **E**, **G** Disruption of glycine from morning cells, but not PDF and sNPF, change seizure rhythmicity. For (**A**, **B***)* *n* = 48 flies/condition, for (**C**, **D**), *n* = 62 flies/condition, for (**E**, **F**), *n *= 65 flies/condition, and for (**B**), *n* = 68 flies in the ‘pdf-Gal4’ condition and *n* = 59 in the ‘pdf > Shmt RNAi’ condition. “pRayleigh” indicates results of the Rayleigh test. Shmt RNAi leads to significant changes in seizure timing as compared to genetic controls as detected using Watson’s two-sample test of homogeneity (*p* < 0.05). **B**, **D**, **F**, **H** Disruption of PDF or sNPF signaling, but not glycine signaling, leads to changes in seizure severity. The number seizures assessed for each condition is given in *()* and listed in order of appearance: ‘wild-type’ (75), ‘*pdfr*^*han5304*^’ (54), ‘pdf-Gal4’ (86), ‘pdf>pdf RNAi’ (131), ‘pdf-Gal4’ (138), ‘pdf > sNPF RNAi’ (117), ‘pdf-Gal4’ (108), and ‘pdf > Shmt RNAi’ (112). A negative binomial model with a Wald test was used for seizure number. A mixed effects model was used for seizure duration. Data are presented as mean values ± SEM. Source data are provided as a Source Data file.

We next sought to understand how morning cells communicate with downstream neurons to drive daily seizure rhythms. Morning cells release multiple neuropeptides include PDF^41–43^, sNPF^44^, and glycine^45^. We quantified spontaneous seizures after picrotoxin feeding in *pdf* receptor (*pdfr*) mutants, *pdfr*^*han5304*^ ^46^ and found a significant decrease in the number and duration of seizures (Fig. 7B). Surprisingly, while there was a decrement in the number of seizures, the daytime seizure clustering was preserved (Fig. 7A). Similar results were seen when RNAi targeted to *pdf* was expressed in morning cells using the *pdf*-Gal4 driver (Fig. 7C, D), though there was an increased number of briefer seizures. sNPF RNAi expressed in morning cells using the *pdf-*Gal4 driver also led to a decrease in the number and severity of predominantly daytime seizures (Fig. 7F). Again, diurnal rhythms in seizures were not perturbed after sNPF knockdown (Fig. 7E). This blunting of daytime seizures in *pdfr*^*han5304*^ mutants, after sNPF knockdown, and to a lesser extent, after PDF knockdown led to a decrease in lethality induced by picrotoxin (Supplementary Fig. 10A, C, E) and could not be explained by a change in daytime or nighttime sleep (Supplementary Fig. 10B, D, F). We note that prior studies have demonstrated changes in sleep amount in *pdfr*^*han5304*^ mutants and after sNPF knockdown^47,48^, but in the presence of picrotoxin, we did not see significant differences.

In contrast, RNAi targeting serine hydroxymethyltransferase (Shmt), an enzyme that catalyzes the conversion of serine to glycine, in morning cells did not change the total number or severity of seizures (Fig. 7H). However, glycine knockdown led to an increase in nighttime seizures with a loss of seizure rhythmicity (pRayleigh n.s.) (Fig. 7G). There was no overall change in lethality (Supplementary Fig. 10G) and no loss of nighttime sleep to explain the increased seizure risk at night (Supplementary Fig. 10H). Taken together, these results reveal that the neuropeptide repertoire in morning cells controls a diurnal switch: PDF and sNPF increase daytime seizures, while glycine decreases nighttime seizures.

**Fig. 8:**
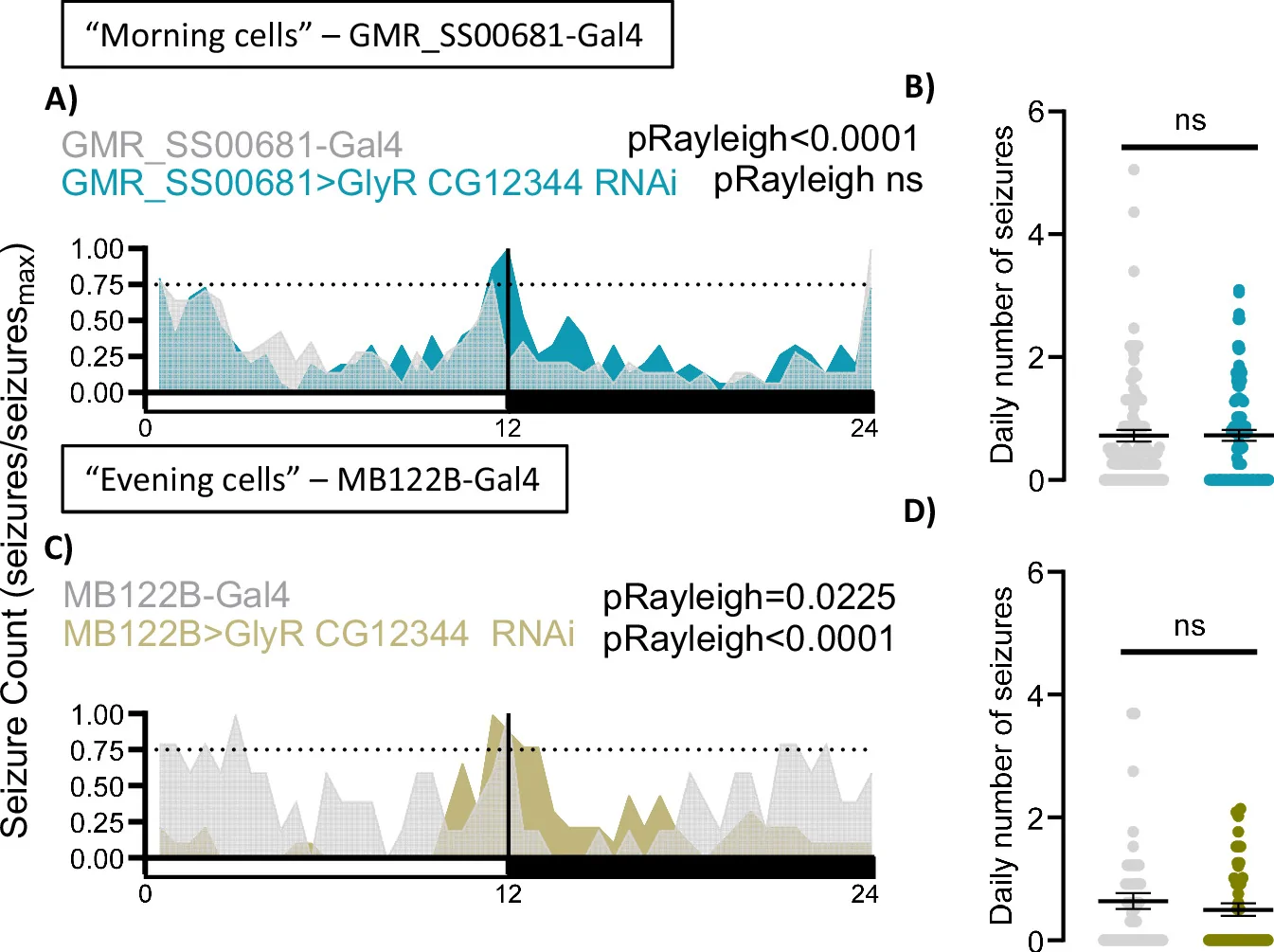
Glycine receptors (CG12344) on evening cells mediate suppression of nighttime seizures. **A**, **C** Knockdown of glycine receptor in evening cells leads to a relative increase in nighttime seizures. *n* = 47-96 flies/condition. “pRayleigh” indicates results of the Rayleigh test. Knockdown of CG12344 in evening cells, but not morning cells, led to a significant change in seizure timing as detected by a Watson’s two-sample test of homogeneity (*p* < 0.05). **B**, **D** There were no significant differences detected in seizure numbers with knockdown of CG12344 in morning cells and evening cells. For (**A**, **B**), *n* = 96 flies/condition. For (**C**, **D**), *n* = 47 flies/condition. Negative binomial model with Wald test was used. Data are presented as mean values ± SEM. Source data are provided as a Source Data file.

To determine the site of glycine action, we concentrated our attention on morning cells and evening cells. Expression of RNAi targeting the glycine receptor subunit CG12344^45^ using the GMR_SS00681-Gal4 driver specific for small LNvs, the morning cells, led to a subtle blunting of seizure rhythms (Fig. 8A) with no change in seizure numbers or sleep (Fig. 8B and Supplementary Fig. 11A, B). In contrast, knockdown of CG12344 in evening cells using the MB122B-Gal4 driver led to a relative shift of seizures to the evening (Fig. 8C) with no significant change in seizure numbers or sleep (Fig. 8D and Supplementary Fig. 11C, D). This resembled the result obtained with glycine knockdown in morning cells and indicates that glycine from morning cells may act on glycine receptors on evening cells at night to decrease seizure risk. As the night progresses, morning cell activity ramps up^49^ and direct synaptic contact with evening cells increases^45,50,51^. Therefore, our data suggest that during this ramping up of morning cell activity through the night, glycine can act on evening cells to suppress nighttime seizures.

### Descending motor neurons are downstream of clock neurons

We finally sought to understand what downstream networks were being controlled by clock neurons to modulate seizure risk. The clonic movements of seizures have been previously ascribed to the giant fiber pathway^52^ composed of large descending motor neurons that are upstream of jump and flight muscles. When a high-frequency stimulus is delivered directly to the descending giant fiber pathway, tonic-clonic movements identical to bang-sensitive seizures are induced^52^. We examined the NeuPrint hemibrain connectome dataset^53,54^ to determine if synaptic connections exist between clock neurons and giant fiber neurons. Using a threshold of 5 synaptic connections, we found that most morning cells (i.e., s-LNv1, s-LNv2, and s-LNv4) are separated by 3 synapses from giant fiber neurons (Supplementary Fig. 12A).

We hypothesized that the control of daily seizure rhythms by clock neurons occurs upstream of giant fiber neurons. Given that disruption of PDF or sNPF signaling from morning cells dampened daytime seizures (Fig. 7A–F), we asked if direct hyperactivation of giant fiber neurons might “rescue” seizure severity. In *pdfr*^*han5304*^ mutants, which exhibit decreased daytime seizures compared to wild-type flies (Fig. 7A, B), we drove expression of csChrimson in giant fiber neurons using the R68A06-Gal4 driver^55^. Activation of giant fiber neurons around the clock led to an increase in seizure duration (Supplementary Fig. 12C) without a significant change in seizure timing (Supplementary Fig. 12B) or lethality (Supplementary Fig. 12D). This increase in seizure severity indicates that giant fiber neurons are downstream of clock neurons.

To assess the role of giant fiber neurons in mediating time-specific inputs from upstream clock neurons, we inhibited giant fiber neurons from ZT0-4, when morning cells are known to be active and worsen daytime seizures. Inhibition of giant fiber neurons with GtCR1 during this narrow time window using the R68A06-Gal4 driver led to a ~ 50% reduction in the daily number of seizures (Supplementary Fig. 12E, F). This inhibition of seizures led to improved survival from 25% to 75% at 96 h (Supplementary Fig. 12G). Together, these data suggest descending motor neurons that mediate seizures are downstream of clock inputs, and that inhibition of downstream motor pathways during relatively narrow windows can be used to decrease seizures and improve survival.

## Discussion

**Fig. 9:**
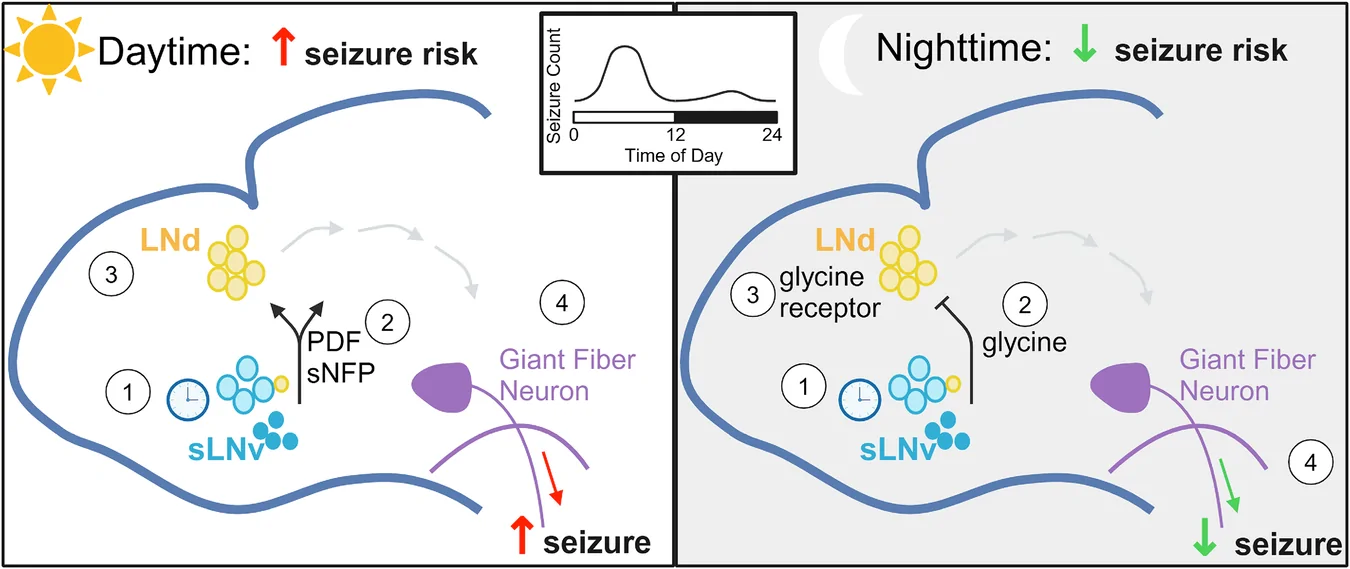
A diurnal switch in clock neurons controls daily seizure rhythms. During the daytime, PDF and sNPF released from morning cells promote seizure likelihood. This likely occurs upstream of the giant fiber neurons that mediate seizures. At night, glycine from morning cells likely acts on glycine receptors on evening cells to suppress seizures. Created in BioRender. Sehgal, A. (2026) https://BioRender.com/xfrjfe4.

While oscillations in seizure risk over 24 h periods have been appreciated from people to mammalian models, the mechanisms driving this cycle have been elusive. After implementing an automated video-based detection system for continuous seizure detection, we find (1) flies exhibit diurnal rhythms in seizures, (2) PDF and sNPF from morning cells are seizure-promoting especially during the daytime, (3) glycine from morning cells likely inhibits nighttime seizures through its action on evening cells, and (4) morning cells are capable of increasing global brain activity, but only during the early day (Fig. 9). While this manuscript was under review, Huertas-Radi et al. reported clock-dependent rhythms in the severity of mechanically induced seizures in bang-sensitive mutants^56^, consistent with our finding that seizure timing is under circadian control. Our data indicate that clock neurons through this diurnal switch uniquely modulate seizure risk, and that this effect, at least partly, occurs through control of descending motor neurons mediating tonic-clonic seizures.

When *tim*^01^ mutants, which globally lack a functional molecular clock, were administered picrotoxin, the timing of spontaneous seizures was disrupted without a difference in total seizure number (Fig. 1B, D, E). However, when *tim*^*01*^ mutants were crossed to *tko*^25t^ bang-sensitive mutants, induced seizures were less likely to occur (Supplementary Fig. 1G), but more severe when they did occur (Supplementary Fig. 1H). We and others have noted similar patterns of reduced seizure inducibility in prior studies of sleep loss^13,57^, whereby seizures are less likely to be induced after sleep loss, but the seizures are more severe when they occur. This is attributed to seizure-prone bang-sensitive mutants being in a refractory state because of spontaneous, non-stimulus-evoked seizures, which were systematically identified with chronic video recording^13^. These spontaneous seizures make the likelihood of inducing seizures in *tko*^25t^*/tim*^*01*^ double-mutant flies less, and complicate the interpretations of the likelihood of seizure induction. Thus, the chronic video recording paradigm we implement here provides an opportunity to more robustly capture daily oscillations in seizure risk.

While controlling seizure timing through the day, we find that the molecular clock does not control the timing of lethal seizures. Lethal seizures peak at night in both wild-type and clock mutant flies between ZT12-18 (Fig. 1F). This nocturnal susceptibility to death related to seizures is reminiscent of sudden unexplained death in epilepsy (SUDEP), the leading cause of death related to refractory epilepsy^58^. Interestingly, the likelihood of SUDEP peaks at night in both people^59,60^ and epilepsy models implementing nocturnal animals^61^. While circadian regulation has been suspected^61^, disentangling the separate contributions of the sleep-wake status, sleep need, and circadian rhythms has been a challenge^62^. The increased risk of lethal seizures at night in clock mutant flies demonstrates that this process is clock-independent; though speculative at this point, one possibility is that lethal seizures in the early evening are driven by sleep need. At ZT12, when the lights are switched from on to off, flies have a high sleep need and fall asleep. Thus, the high lethality at night could be tied to (a) the sleep state, (b) a transition from wake to sleep, or (c) a high sleep need. Therefore, further studies examining susceptibility to lethal seizures during sleep-wake transitions or during times of high sleep need are warranted.

Here we find that (a) spontaneous seizures arising from picrotoxin treatment and (b) induced seizures in *tko*^25t^ mutant flies both peaked in severity during the day (Figs. 1B, 2B). This peak in seizure severity is remarkably similar in phase to the daytime peaks in seizure risk previously identified in people with epilepsy^1,4^. Using chronic video recording with automated seizure detection, we find that this daytime seizure timing is driven predominantly by PDF and sNPF release from PDF+ morning cells in flies (Fig. 7). We separated the contributions of small and large LNvs by targeting clock function in small LNvs specifically (Supplementary Fig. 6) and found the emergence of nighttime seizure peaks. The emergence of these nighttime peaks may be due to the loss of nighttime glycine release, which suppresses nighttime seizures. Aligning the seizure rhythms we observe in *tko*^25t^ mutants and picrotoxin-treated flies with prior calcium imaging data from clock neurons^49,63^ demonstrates that the phase of peak seizure risk overlaps with calcium peaks in both small and large LNvs. Together, these data suggest that increases in seizure risk in the early day are driven by small LNvs while mid-day seizures are predominantly driven by large LNvs. In children and adults with epilepsy, early-to-mid day peaks in seizure risk are also seen in focal seizures arising from the frontal, parietal, and temporal lobes^5,63^.

The molecular clock does not drive a single “seizure time” for all epilepsies. Instead, the molecular clock likely drives rhythms in physiology that interact with distinct genetic and structural etiologies to produce individualized peak seizure times. For example, for the picrotoxin-induced seizures and bang-sensitive seizures in *tko*^25t^ mutants we study here, seizure severity was greatest during the daytime, but in *para*^*GEFS+*^ and *para*^*DS*^ flies, temperature-sensitive seizures clustered at the day-to-night transition. In people with epilepsy, sleep-related hypermotor epilepsy is typified by nighttime seizures, and pediatric focal seizures arising from the occipital lobe can arise in the early evening^5^. These nighttime predominant seizures might result from regulation by other clock neurons, e.g., LNds, that are predominantly active in the evening^49,64^. Just as the multiple circadian phases of downstream non-clock neurons are set by various clusters of clock neurons^64^, the phase-shifting of seizure risk might be predominantly controlled by these same subclasses of clock neurons. Alternatively, genes implicated in monogenic epilepsies, including *SCN1A* (orthologous to the *paralytic* gene in flies we study here), *KCND2*^65^, and *KCNA4*^66^, also control firing of neurons in the suprachiasmatic nucleus^67,68^, which might alter the phase of seizure timing.

It is important to note that clock neurons are not static and instead undergo diurnal structural plasticity. Synaptic terminals of *Pdf*+ morning cells in the fly^37,50,51^ and vasoactive intestinal peptide+ neurons in the mouse suprachiasmatic nucleus^50^ undergo diurnal remodeling whereby synaptic complexity is highest during the day. Beyond simply strengthening connections with existing synaptic partners, morning cells are known to synapse with different neurons at different times of the day^50^. Thus, although we optogenetically stimulated *Pdf *+ neurons throughout the day and night, seizure risk and brain activity may have preferentially increased during the day (Figs. 5B, 6D, E) because this is when downstream connectivity to ictogenic circuits was also peaking. Similarly, morning cells increase contact with evening cells at night^45,50^, and this provides an explanation for why glycine from morning cells has a preferential effect on evening cells at night to suppress seizures (Figs. 7G, H, 8C, D). Thus, the time-of-day specific effects of neuromodulators we identify here may largely be driven by oscillations in structural plasticity. A limitation of our in vivo two-photon experiments was that upon activation of *Pdf*+ neurons, we assessed whole brain activity instead of activity of specific brain regions. While it is remarkable that activation of *Pdf*+ neurons in the morning can increase brain-wide activity, future studies should attempt to construct more specific anatomical and temporal maps of activation using standardization techniques, including BIFROST mapping^69^.

Here, beyond noting the structural connections as identified by electron microscopy (Supplementary Fig. 12A), we do not explore how clock neurons and giant fiber neurons are functionally connected due to technical limitations in calcium imaging of giant fiber neurons. The neuPrint hemibrain connectome reveals that as few as 3 synapses are required for communication from morning cells to giant fiber neurons^53,54^. These synapses occur at the (1) superior lateral protocerebrum (SLP), (2) anterior ventrolateral protocerebrum (AVLP), and (3) giant fiber neuron. The SLP functions as a hub for sensory processing with dense connectivity with mushroom body neurons^70,71^. The AVLP contains a cluster of descending motor neurons^71^. Finally, the giant fiber neuron mediates the startle reflex in flies and is upstream of the tergotrochanter and dorsal longitudinal wing-depressor muscles. Thus, the tonic-clonic movements that characterize the seizures we describe here may arise from hyperexcitability at the level of the AVLP and giant fiber neuron.

While the fly model offers valuable insights into mechanisms controlling seizure timing, translating these findings to human epilepsies presents inherent limitations. The mammalian brain utilizes complex networks across billions of neurons of various subtypes. In contrast, the fly brain contains approximately 140,000 neurons and lacks a cerebral cortex, which is the primary seat of seizures in humans. The specific brain regions driving fly seizures, beyond the giant fiber system, remain unmapped. Seizures in people are defined by both stereotyped behavioral and electrographic features. Because obtaining electrical access in freely moving flies is technically challenging, fly seizures have been predominantly described using behavioral readouts. Consequently, seizures that do not manifest as sustained tonic-clonic movements are likely undetected. Nevertheless, the fundamental molecular mechanisms governing neuronal excitability are highly conserved between flies and humans. This includes voltage-gated ion channels, synaptic release machinery, and molecular clock machinery. Despite its anatomical simplicity, the fly model not only exhibits seizures but also demonstrates daily rhythms in seizure susceptibility, providing a powerful reductionist system for identifying conserved seizure triggers.

Our findings complement the Molecular Oscillations and Rhythmicity of Epilepsy (MORE) hypothesis^72^ well. The MORE hypothesis posits that diurnal molecular rhythms in the brain place the brain in oscillating levels of seizure risk through the day. We find this occurs in the fly through rhythmic release of PDF, sNPF, and glycine from clock neurons through the day. Seizure forecasting is receiving growing attention as a strategy to predict and inactivate seizures before they occur in people with refractory epilepsy^73^. Our data demonstrate that accounting for contributions of clock neurons to circadian seizure cycles will enhance our ability to predict times of greatest seizure susceptibility.

## Methods

### *Drosophila* melanogaster

Wild-type Canton-S or w^1118^ flies were in this study. UAS, Gal4, and mutant fly lines already not in the lab or gifted were obtained from Bloomington *Drosophila* Stock Center in Indiana, USA. The following fly stock were used in this study: *tko*^25t^ (gift from Dr. Dan Kuebler), *tim*^*01*^ (Lab stock^15^), *pdfr*^*han5304*^ (Bloomington *Drosophila* Stock Center (BDSC) RRID:BDSC_33068), Clk856-Gal4 (gift from Dr. Michael Rosbash), repo-Gal4 (Lab stock), tim-Gal4 (Lab stock), cry-Gal4 (Lab stock), pdf-Gal4 (Lab stock), R18H11-Gal4 (RRID:BDSC_48832), R65D05-Gal4 (RRID:BDSC_39351), GMR_MB122B-Gal4 (H. Dionne, A. Nern, and G. Rubin, Janelia Research Center, VA), GMR_SS00681-Gal4 (H. Dionne, A. Nern, and G. Rubin, Janelia Research Center, VA), R68A06-Gal4 (RRID:BDSC_39449), R57C10-lexA (RRID:BDSC_52817), UAS-tim gRNA, UAS-Cas9 (RRID:BDSC_90768)^24^, UAS-per gRNA, UAS-Cas9 (RRID:BDSC_90769)^24^, UAS-pdf RNAi (RRID:BDSC_25802), UAS-sNPF RNAi (RRID:BDSC_25867), UAS-Shmt RNAi (RRID:BDSC_57739), UAS-CG12344 RNAi (RRID:BDSC_26250), UAS-TrpA1 (gift from Leslie Griffith)^74^, UAS-csChrimson (Lab stock), UAS-GtACR1 (RRID:BDSC_92983), LexAOp-GCaMP7b (RRID:BDSC_80915), UAS-CD8::RFP, LexAop-CD8::GFP-2A-CD8::GFP; UAS-mLexA-VP16-NFAT, LexAop-CD2::GFP (UAS-CaLexA, lab stock)^75^.

Unless otherwise noted, mated male flies aged 5–10 days post-eclosion were used. Flies were maintained in incubators with a 12 h light:12 h dark cycle at 25 °C and approximately 60–65% relative humidity. Flies were maintained on a standard cornmeal-molasses diet, including 64.7 g/L cornmeal (LabScientific FLY-8010-20), 27.1 g/L dry yeast (LabScientific FLY-8040-10), 8 g/L agar (Fisher NC1429200), 61.6 mL/L molasses (Genesee Scientific 62-118), 10.2 mL/L 20% tegosept, and 2.5 mL/L propionic acid.

### Video tracking analyses of spontaneous seizures and sleep

Flies were placed into 24- or 48-well plates containing agar (2%) and sucrose (5%) with vehicle or drugs mixed into the agar. A toxicity assay was performed to initially determine picrotoxin (FisherAC131210010) dosing. For each experiment, 0.25-0.5 mg/ml of picrotoxin was used. For assays in which a ceiling effect was observed, the picrotoxin concentration was reduced to 0.05 mg/ml. Flies were then placed into individuals well and sealed into the wells with a plastic film (PerkinElmer TopSeal A Plus; 6050185). Small holes were introduced into the film overlying wells using a needle to allow for air and humidity flow. The plates were then placed into a *Drosophila* incubator with light, temperature, and relative humidity control. Individual plates were illuminated with an infrared (IR) light and visualized with an IR camera (monochrome GigE camera with IR pass filter) to allow for imaging in light-dark conditions. Videos were collected at 25 frames per second for 96 h per experiment.

EthoVision XT (Noldus Information Technology) was used to track the position of the fly body over time, yielding XY coordinates over time. As previously described^13^, thresholds for ‘velocity’, ‘acceleration’, ‘mobility’, ‘mobility state’, and ‘movement’ were developed to define a “hyperkinetic event” (HE). Seizures were subsequently defined as 5 HE occurring in 50 s, with at least 10 HE per seizure. As previously described^13^, thresholds were developed such that wild-type flies (Canton-S, w^1118^, and iso^31^) on vehicle never exhibited movements that were defined as seizures. Movement^76^ and sleep were quantified as previously described^13^.

For optogenetics experiments, flies were raised in the dark. (1) For csChrimson experiments at the time of entrainment in blue light for 48 h, flies were placed in all-trans retinal (ATR) (300 μM). After entrainment, flies were then transferred to 24- or 48-well plates containing picrotoxin and ATR with red light stimulation. Red lights were turned on for 5 minutes and turned off for 25 min at the times detailed in the Fig. (2) For GtACR1 experiments at the time of entrainment in red light for 48 h, flies were placed in ATR (1 mM). After entrainment, flies were then transferred to 24- or 48-well plates containing picrotoxin and ATR with green light stimulation. Green lights were turned on for 5 min and turned off for 25 min at the times detailed in the figures.

### Quantification of bang-sensitive seizures

As previously described^13^, *tko*^25t^ bang-sensitive mutants were placed in groups of 4 flies into vials containing agar (2%) (Fisher NC1429200) and sucrose (5%). Vials were struck on a countertop four times and then vortexed on a Fisher Vortex Genie 2 (#12-812) at setting ‘5’ for 5 s. Vials were then placed in front of a camera for video recording on a PC using OBS Studio 29.0. After the experiment, videos were manually scored for the percentage flies with seizure in each vial, duration in the atonic (“paralysis”) phase, duration in the tonic/clonic (“convulsive”) phase, and duration in the postictal (“recovery”) phase for each individual fly. Scorers were blinded when possible. To achieve the multiple timepoints tested, separate sets of flies were entrained in incubators on different light-dark cycles.

For the Gal4 screen of clock neuron subsets using UAS-TrpA1, flies were raised at 18 °C to minimize TrpA1 activation. Flies were then placed in a 25 °C water bath for 4 min. Vials were quickly removed and seizures induced as described above. Vials were then returned to the water bath at 25 °C with subsequent video recording for seizure scoring.

### Quantification of behavioral rhythmicity using multibeam infrared monitors

Flies were placed into locomotor tubes containing agar (2%) and sucrose (5%). Locomotor tubes were loaded into DAM5H multibeam monitors (Trikinetics) and placed in complete darkness for 4 days for quantification of behavioral rhythmicity. After 4 days, a 12 h light:12 h dark cycle was introduced for 3 days. ClockLab software was subsequently used to generate actograms and quantify behavioral rhythmicity.

### CAFE assay

Flies were collected within 24 h post-eclosion and aged for 3–5 days while maintained in a 12 h light:12 h dark cycle at 25°C. Canton-S (wild type) and *tim*^*01*^ mutant flies were placed into custom CApillary FEeder (CAFE) assay chambers: four glass capillaries (1 mm inner diameter) filled with 5 µL of 5% sucrose solution embedded in plastic wide-mouth vials with soft plugs. Continuous feeding was evaluated in the morning (ZT0-5) and evening (ZT12-17). Fluid levels were measured before and after each time window. Capillaries from empty control vials (without flies) served as an evaporation control. Feeding intake was normalized per fly and compared across genotypes and timepoints.

### CaLexA imaging

CaLexA imaging was performed using adult male flies as previously described^13^.

### Two-photon in vivo imaging

Experimental female flies aged 5–10 days were raised in the dark. Female flies were used as they are larger and more amenable to microdissection. Flies were then transferred to ATR (300 μM) (Sigma-Aldrich R2500) and entrained in a box that filtered red light out on a 12 h light:dark cycle for 72 h. Flies were then collected at 4 timepoints through the day and quickly anesthetized with ice. Flies were then mounted by head and thorax to a fixed position on a perfusion chamber with cuticle dissection to expose the dorsal brain (Murthy and Turner, 2013). Cuticle dissections were performed in a dark room with diffuse, low light illuminating the field of dissection.

This preparation was mounted over an air-suspended ball (600 mL air/second) and imaged using a Bruker Nanosystems Ultima two-photon microscope in an anterior-posterior imaging plane. Images were acquired using a Chameleon ULTRA I laser directed through a resonant scanning galvanometer with a 20X Olympus objective mounted on a piezo-electric Z-focus. Images were acquired with 7-8 z-sections (30–35 μm steps). Brains were allowed to equilibrate for 1 h from the time of fly mounting to the initiation of recording on the microscope. Fresh saline was continuously perfused over the brain from the time of dissection to the end of the experiment. Three stimulation protocols were used to stimulate csChrimson using a red 660 nm LED: (1) a 10 millisecond pulse, (2) a 100 millisecond pulse, and (3) a 1-second 30 Hz stimulation^77^. Only data from the 100-millisecond pulse are presented. GCaMP7b signal was measured for 5 min before each stimulation and 5 min after each stimulation. All flies with any GCaMP7b signal response were included for analysis. Data were analyzed by summing intensities across each Z-stack and computing the mean pixel intensity within a manually drawn region of interest covering the entire brain. Signal was normalized by dividing by the mean value in the 12 seconds prior to stimulation. Peak intensity was computed as the average of values between 0–20 s after stimulation. Steady-state intensity was computed as the average of values between 20–100 s after stimulation.

### Statistics & reproducibility

We used the Shapiro-Wilk test and Kolmogorov-Smirnov tests as pre-tests to assess normality and the need for parametric or non-parametric testing. For significance testing between two groups, the unpaired two-tailed *t* test was used for data that were reasonably assumed to be approximately normally distributed. When we compared three or more groups with multiple comparisons, a one-way ANOVA with Tukey’s multiple comparisons test was used for normally distributed data unless otherwise indicated.

A negative binomial model with a Wald test was implemented for hypothesis testing of spontaneous seizure frequency, as we have demonstrated^13^. For seizure duration and hyperkinetic events per seizure, a mixed-effects model was implemented to account for both intra- and inter-fly variability, given that some flies had multiple seizures.

For spontaneous seizures, the Rayleigh test was used to test the null hypothesis that the distribution of the seizures is uniform across a 24 h period. To assess the contribution of single flies having multiple seizures, we implemented a permutation method done by rotating the timing of the clock for each individual in a random fashion^14^. To compare the timing of spontaneous seizures between groups, a Watson two-sample test of homogeneity was used to ask whether the distribution of events differs across two populations. The null hypothesis was that the two groups are samples from the same population. In rose plots, the radius of each bar indicates the normalized count of seizure episodes occurring during the given time point.

JTK_CYCLE was used to identify daily rhythms in seizure duration for induced seizures, and MetaCycle was used to detect cycles in CaLexA signal^78,79^.

Mean and standard error of the mean are used to visually represent the data. Additional details about the sample size (n) for each experiment, statistical testing, and p-values are provided in the Figures and/or Figure Legends. GraphPad Prism was used for all statistical analyses, except for significance testing of spontaneous seizure frequency, seizure duration, hyperkinetic events per seizure, Rayleigh test, and Watson’s two-sample test of homogeneity, which were performed in R version 4.2.1. Rose plots were rendered using custom scripts in Python.

## Supplementary information

Supplementary Information

Description of Additional Supplementary Files

Supplementary Video 1

Supplementary Video 2

Transparent Peer Review file

## Source data

Source data

## Data Availability

Data generated in this study are provided in the Supplementary Information/Source Data file. Source data are provided in this paper.

## Code availability

Our scripts to detect seizures, circadian timing, sleep, and survival from video tracking data, including the “CynthiSeize” script with detailed user instructions, sample data, and sample outputs, is deposited on Zenodo (https://zenodo.org/records/15619740).
