# Supplementary material for: Clock neurons modulate seizure timing through a diurnal switch
Source: Nat Commun. 2026 Sep 30;17:11241. doi: 10.1038/s41467-026-77884-4 (PMC13720085; doi:10.1038/s41467-026-77884-4)
Supplement: Supplementary file 1 [file 41467_2026_77884_MOESM1_ESM.pdf]

Supplementary Figures

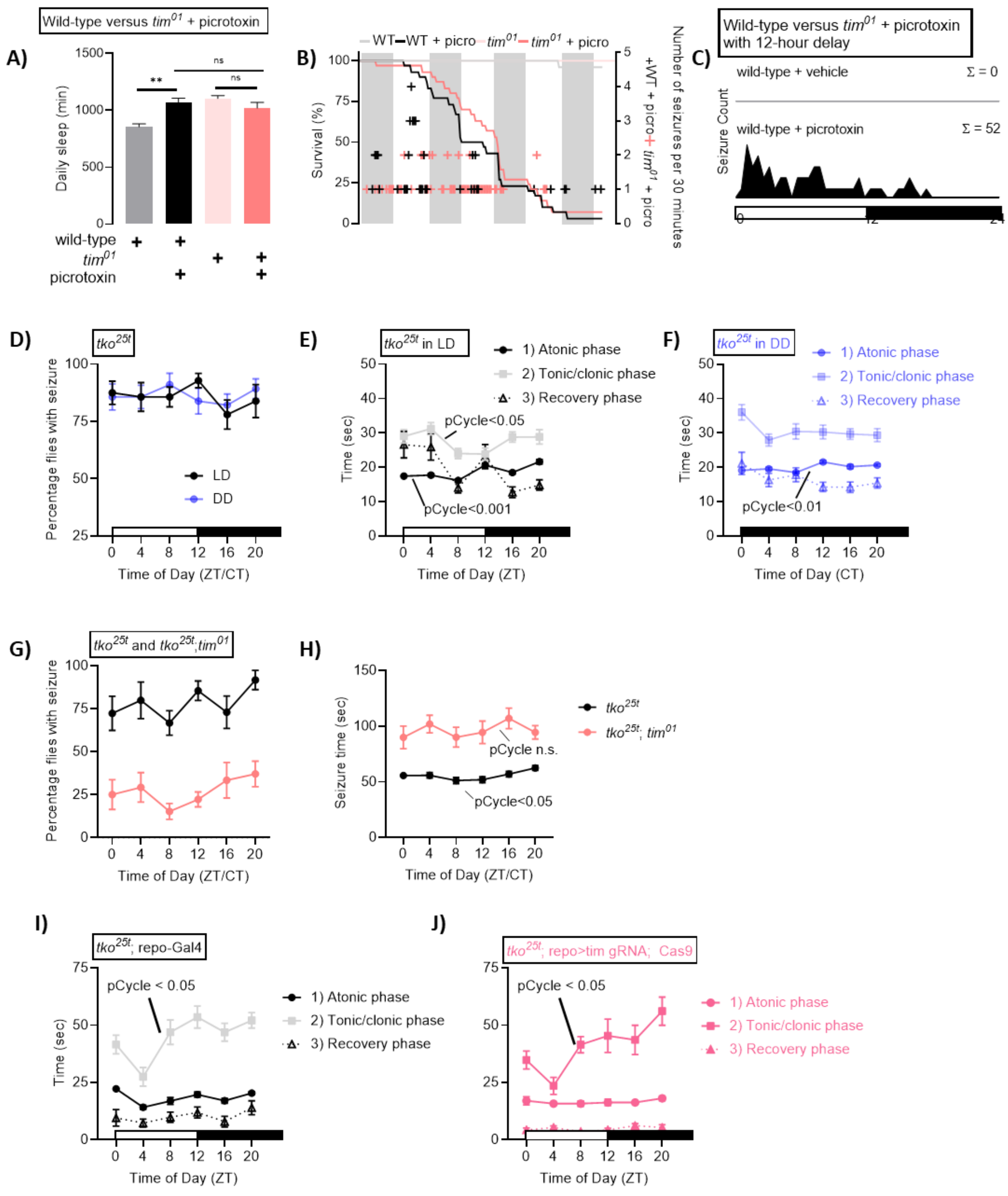

Supplementary Fig. 1 | Profiling of spontaneous and induced seizures across the day. a, Picrotoxin treatment does not induce sleep loss, which is known to increase seizure risk independent of circadian rhythms. n=23-30 flies/condition. One-way ANOVA with Tukey's multiple comparisons test. **b,** Survival curves for picrotoxin-treated flies demonstrating similar survival in wild-type and *tim*^{01}-treated flies. Vertical gray boxes indicate night. "+" indicate number of seizures/30-minute time bin. **c,** Placement of wild-type flies on picrotoxin 12h hours later continues to induce daytime-predominant seizures. **d,**

Percentage of *tko*^{25t} flies experiencing seizures after seizure induction protocol through the day. n=14 vials/condition. There is no rhythm in likelihood of seizure induction as identified by JTK cycle analysis. **e,** *tko*^{25t} flies maintained in a 12-hour light:12-hour dark cycle (LD) exhibit a diurnal rhythm in the atonic phase and tonic clonic phase of induced seizures per JTK cycle analysis (pCycle). n=42-51 seizures/condition. **f,** *tko*^{25t} flies maintained in constant darkness (DD) exhibit a diurnal rhythm in the atonic phase per JTK cycle analysis (pCycle). n=40-47 seizures/condition. **g,** Induced seizures are less likely to occur through the day in *tko*^{25t}/*tim*^{01} flies as compared to *tko*^{25t} flies. n=12 vials/condition. **h,** *tko*^{25t}/*tim*^{01} flies exhibit no rhythms in seizure duration through the day as measured by JTK cycle. n=6-15 seizures/conditions. A diurnal rhythm of seizure duration is detected in *tko*^{25t} flies with JTK cycle analysis. n=28-41 seizures/condition. Note the lower number of seizures quantified in *tko*^{25t}/*tim*^{01} flies because of the lower rate of seizure induction. **i,** In *tko*^{25t} flies expressing only repo-Gal4, a glial driver, a diurnal rhythm in the duration of the tonic/clonic seizure phase is detected by JTK analysis. n= 9-19 seizures/condition. **j,** In *tko*^{25t} flies expressing tim gRNA and Cas9 under control of the repo-Gal4 driver, a diurnal rhythm in the duration of the tonic/clonic phase continues to be detected by JTK analysis. n= 10-18 seizures/condition. **$p<0.01$. Data are presented as mean values ± SEM.

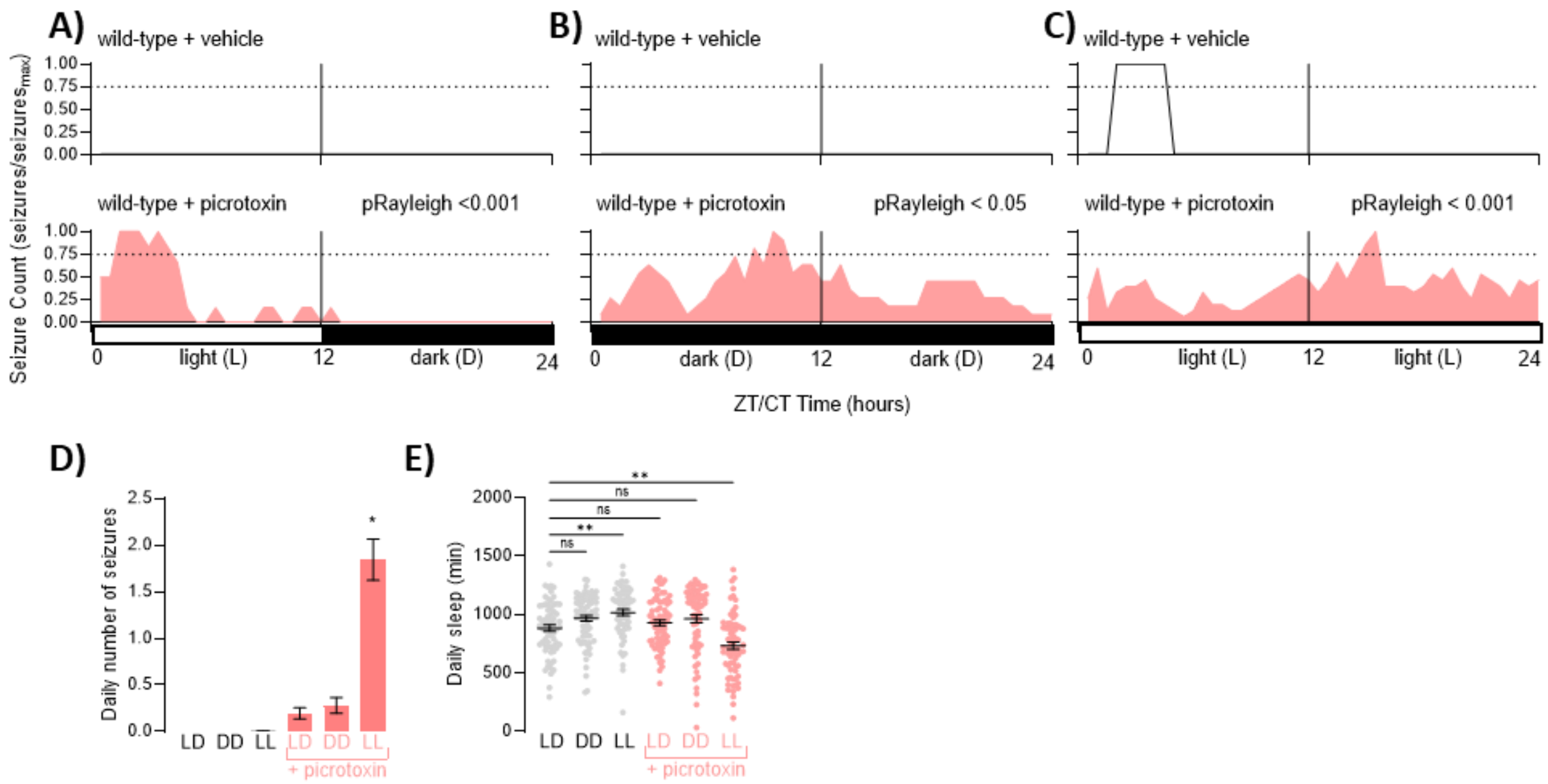

Supplementary Fig. 2 | Daytime seizure predominance persists in constant darkness and is lost in constant light. a, Flies fed picrotoxin have predominantly daytime seizures in a 12-hour light:12-hour dark cycle (LD). **b,** Flies fed picrotoxin continue to have predominantly daytime seizures in constant darkness (DD). **c,** In constant light (LL), rare seizures occur in wild-type flies not fed picrotoxin. When flies are fed picrotoxin, seizures shift in timing with a relative increase in nighttime seizures. "pRayleigh" indicates results of Rayleigh test. n=64-72 flies/condition. **d,** Quantification of daily number of seizures reveals a large increase in seizure counts when flies are in constant light (LL). n=64-72 flies/condition. Negative binomial model with Wald test was used. **e,** Daily sleep is significantly decreased when flies are kept in constant light (LL) and treated with picrotoxin. n=64-72 flies/condition. One-way ANOVA with Tukey's multiple comparisons test.*$p<0.05$ **$p<0.01$ ****$p<0.0001$. Data are presented as mean values ± SEM.

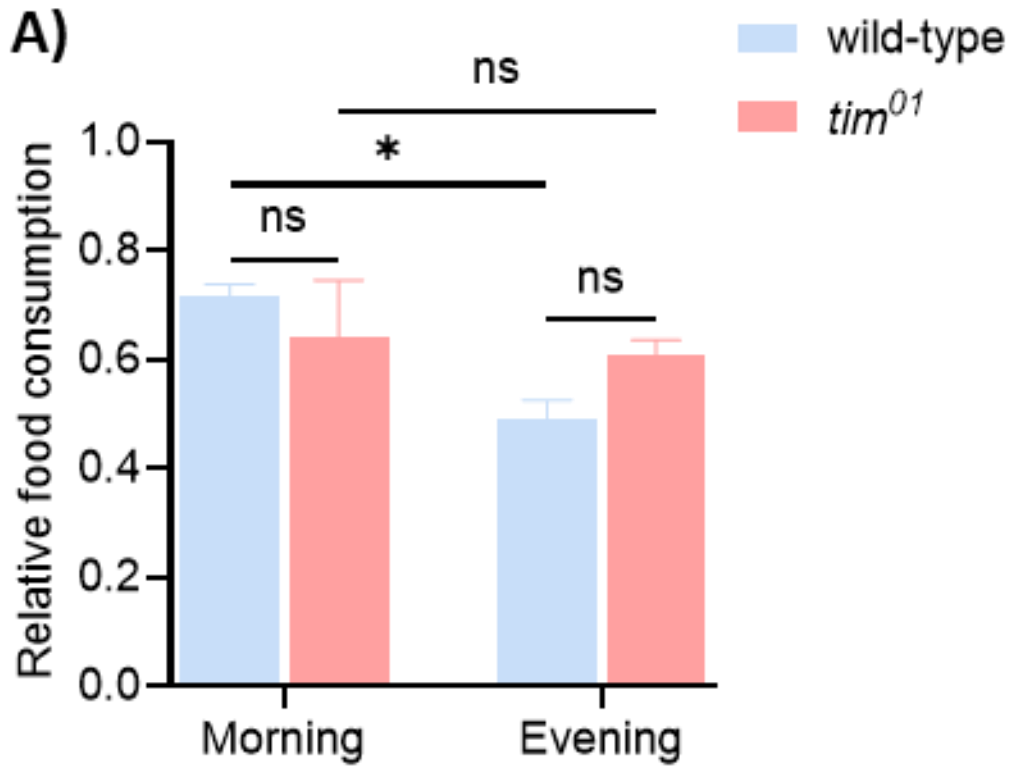

Supplementary Fig. 3 | *tim^{01}* flies maintained in a 12-hour light:12-hour dark cycle consume similar amounts of food as compared to wild-type flies. a, Capillary Feeder Assay was performed at ZT 0-5 (morning) and ZT 12-17 (evening) in wild-type and *tim^{01}* flies housed in a light:dark cycle. n=3 independent experiments/group. Two-way ANOVA was performed. *p<0.05. Data are presented as mean values ± SEM.

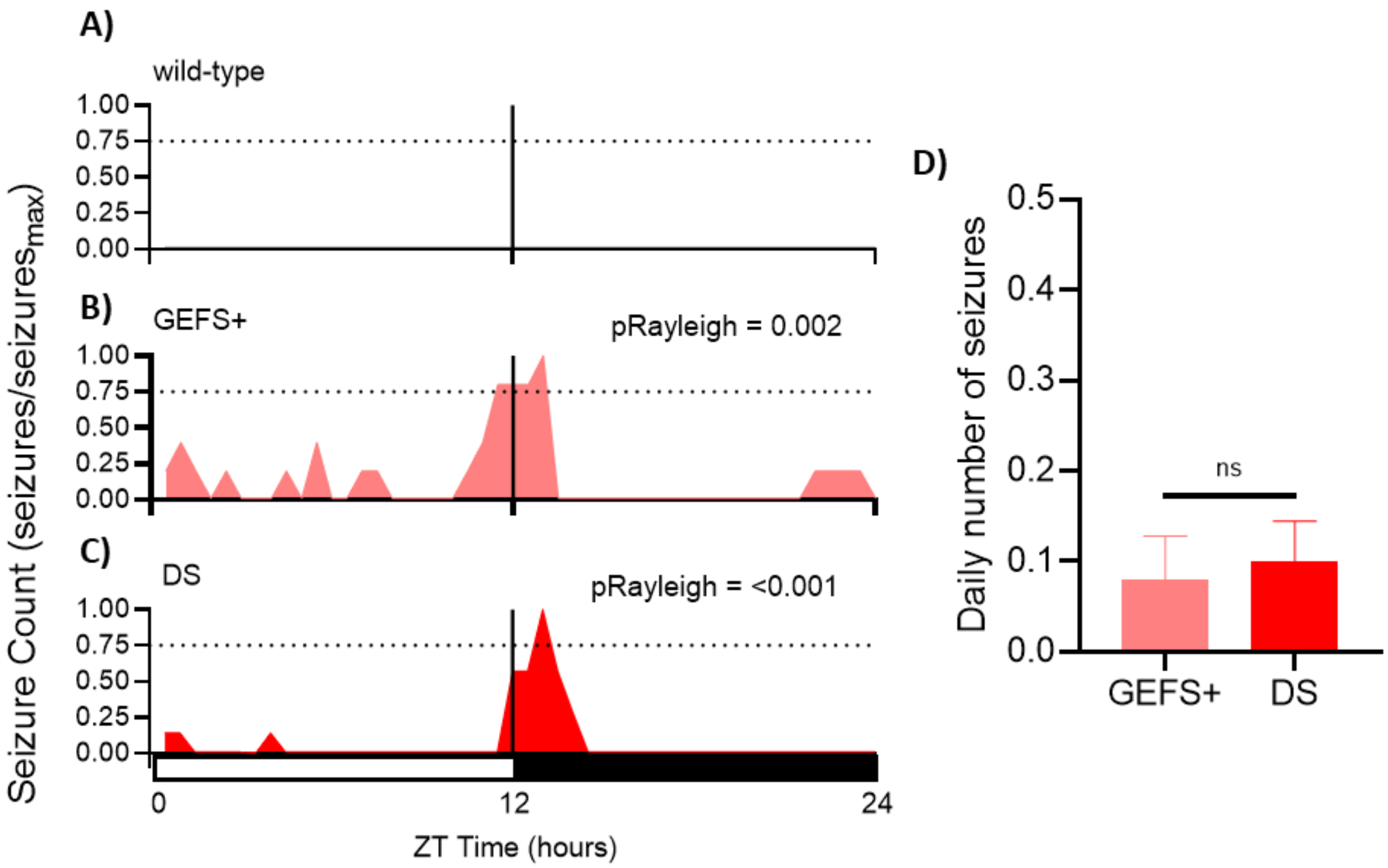

Supplementary Fig. 4 | *para*$^{GEFS+}$ and *para*^{DS} flies exhibit seizures that cluster at the day:night transition. a-c, Wild-type, *para*$^{GEFS+}$, and *para*^{DS} flies were maintained at 30°C for four days in a 12-hour light:12-hour dark cycle. *para*$^{GEFS+}$, and *para*^{DS} flies exhibited seizures that clustered at the light:dark transition. "pRayleigh" indicates results of Rayleigh test. **d,** There were no significant differences in seizure numbers between *para*$^{GEFS+}$, and *para*^{DS} flies maintained at 30°C. Two-tailed t-test was performed. n=24 flies/condition.

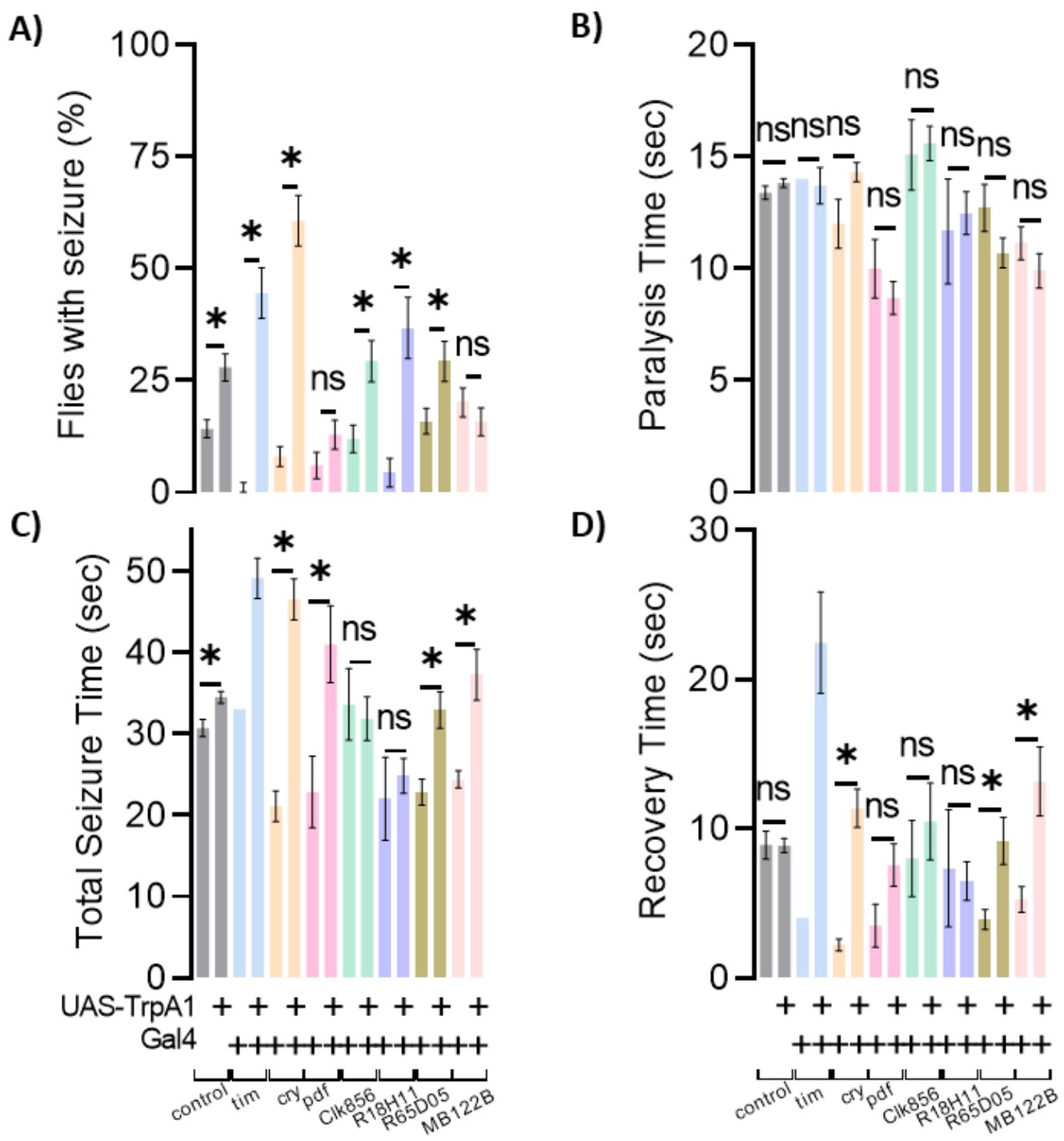

Supplementary Fig. 5 | A Gal4 screen of clock neurons reveals subtypes that acutely worsen induced seizures. a-d, *tko*^{25t} flies exhibited prolonged total seizure times when TrpA1 expression was driven by the *cry*-Gal4, *Pdf*-Gal4, R65D05-Gal4, and MB122B-Gal4 drivers. Paralysis times were not significantly different as compared to genetic controls. For unknown reasons, in *tim*-Gal4;*tko*^{25t} flies, only 1 out of 96 flies tested exhibited a seizure so significance testing is not performed for this group. n=1-67 seizures/condition. Two-group two-tailed t-test. *p<0.05. Data are presented as mean values ± SEM.

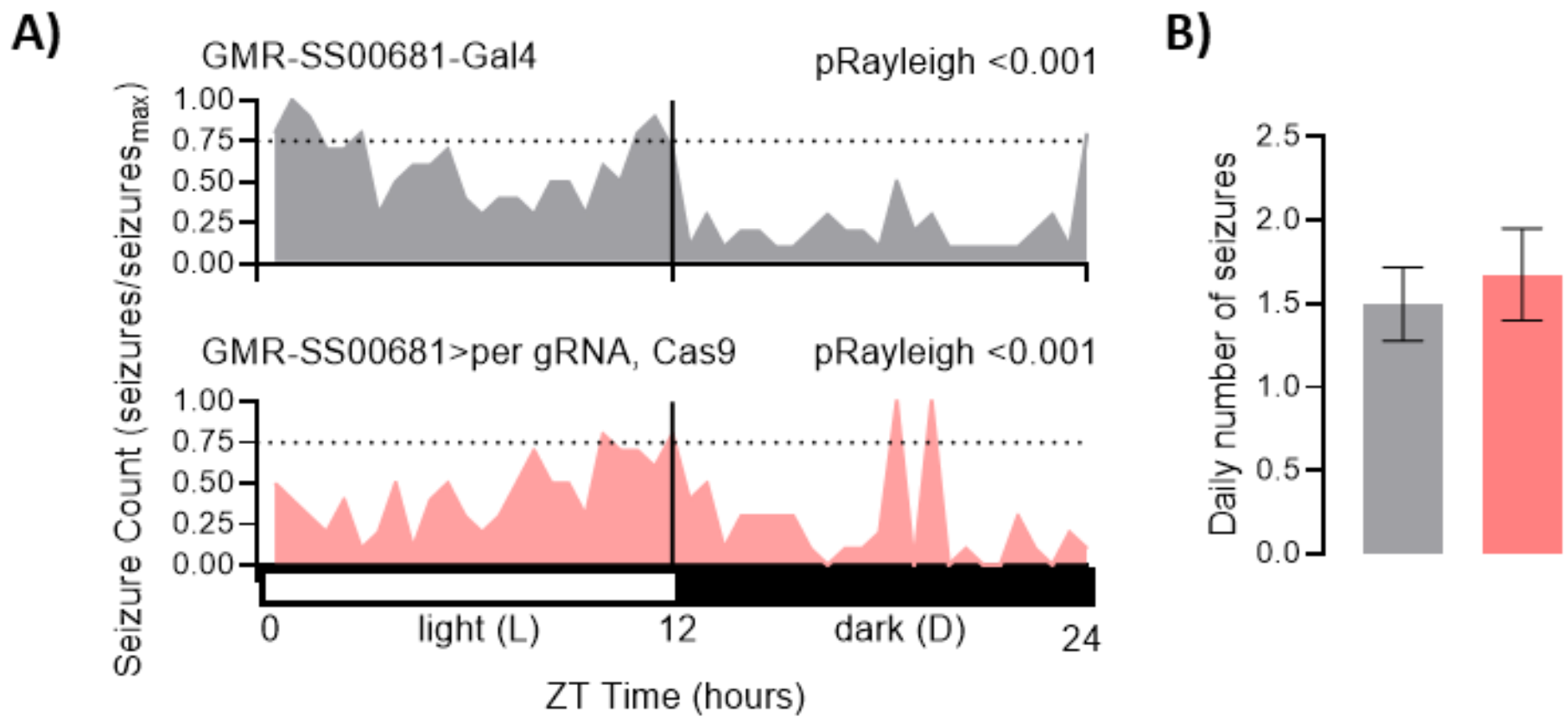

Supplementary Fig. 6 | Perturbation of the molecular clock in small ventrolateral neurons with the GMR-SS00681-Gal4 driver increases nighttime seizures. a, Disruption of the molecular clock in small ventrolateral neurons using the GMR-SS00681-Gal4 driver leads to a nighttime peak in seizures. "pRayleigh" indicates results of Rayleigh test. **b,** Molecular clock disruption with per gRNA in small ventrolateral cells does not affect daily seizure number. n=96 flies/condition. Negative binomial model with Wald test was used. Data are presented as mean values ± SEM.

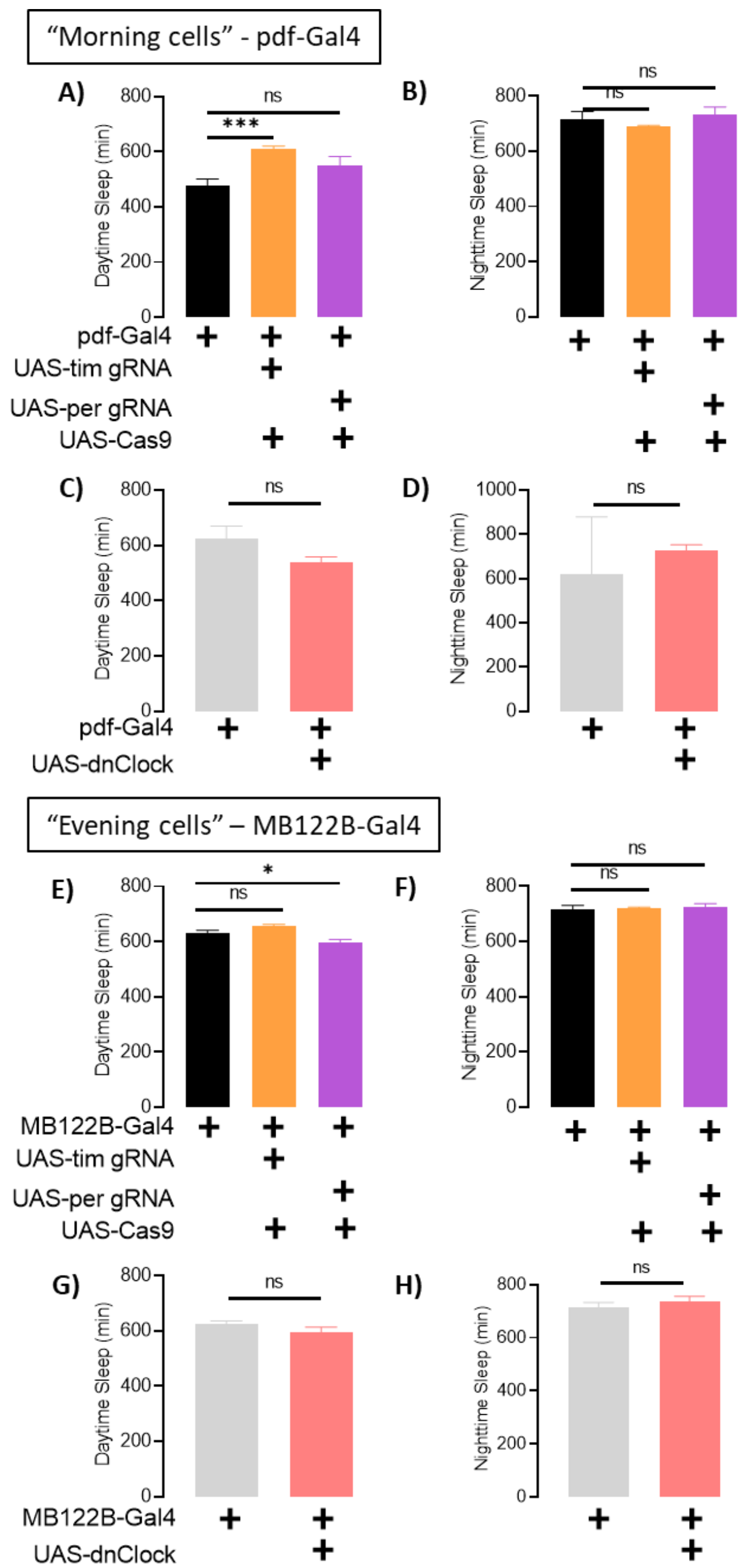

Supplementary Fig. 7 | Changes in sleep quantity do not explain loss of seizure rhythms after molecular clock disruption in morning and evening cells. a-b, Daytime and nighttime sleep, respectively, in genetic controls and after disruption of the molecular clock with tim gRNA or per gRNA in morning cells using the *Pdf*-Gal4 driver reveals no consistent changes in sleep to explain the loss of diurnal rhythms in seizures. n=28-32 flies/condition. One-way ANOVA with Tukey's multiple comparisons test. **c-d,** Daytime and nighttime sleep, respectively, in genetic controls and after disruption of the molecular clock with dnClock in morning cells using the *Pdf*-Gal4 driver reveals no changes in sleep to explain the loss of diurnal rhythms in seizures. n=3-35 flies/condition. Two-group two-tailed t-test. **e-f,** Daytime and nighttime sleep, respectively, in genetic controls and after disruption of the

molecular clock with tim gRNA or per gRNA in evening cells using the MB122B-Gal4 driver reveals no consistent changes in sleep to explain the loss of diurnal rhythms in seizures. n=45-47 flies/condition. One-way ANOVA with Tukey's multiple comparisons test. **g-h,** Daytime and nighttime sleep, respectively, in genetic controls and after disruption of the molecular clock with dnClock in evening cells using the MB122B-Gal4 driver reveals no changes in sleep. n=43-45 flies/condition. Two-group two-tailed t-test. *p<0.05 ***p<0.001. Data are presented as mean values ± SEM.

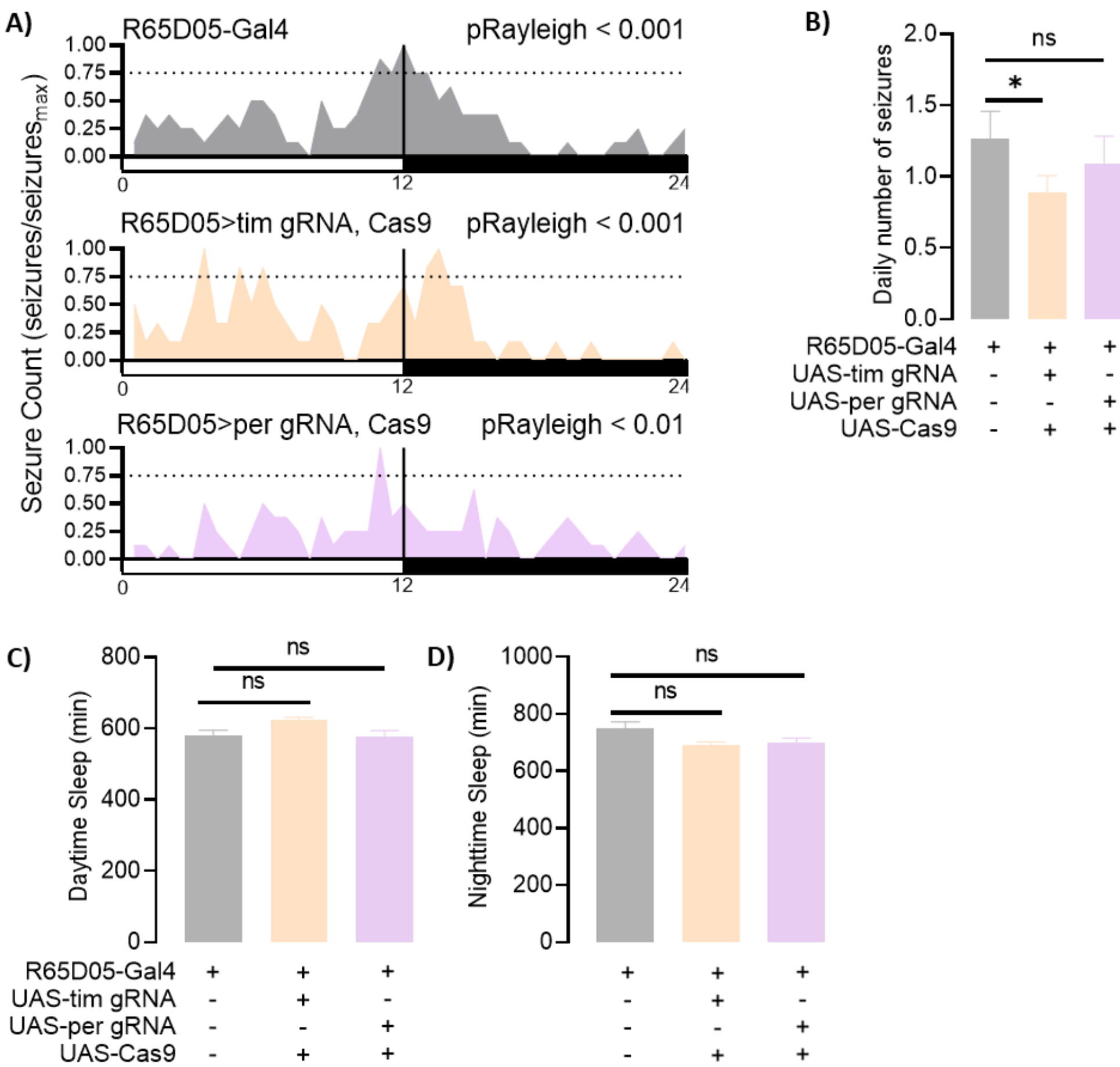

Supplementary Fig. 8 | Perturbation of the molecular clock in LPN clock neurons does not shift seizure rhythms. a, Disruption of the molecular clock in LPN clock neurons using the R65D05-Gal4 driver does not disrupt seizure rhythms. n=62 flies/condition. "pRayleigh" indicates results of Rayleigh test. **b,** Molecular clock disruption with tim gRNA, but not per gRNA, leads to seizure suppression. n=83-109 seizures/condition. Negative binomial model with Wald test was used. **c-d,** Daytime and nighttime sleep, respectively, in genetic controls and after disruption of the molecular clock with tim gRNA or per gRNA in LPN neurons using the R65D05 driver reveals no changes in sleep. n=36-47 flies/condition. One-way ANOVA with Tukey's multiple comparisons test. *p<0.05. Data are presented as mean values ± SEM.

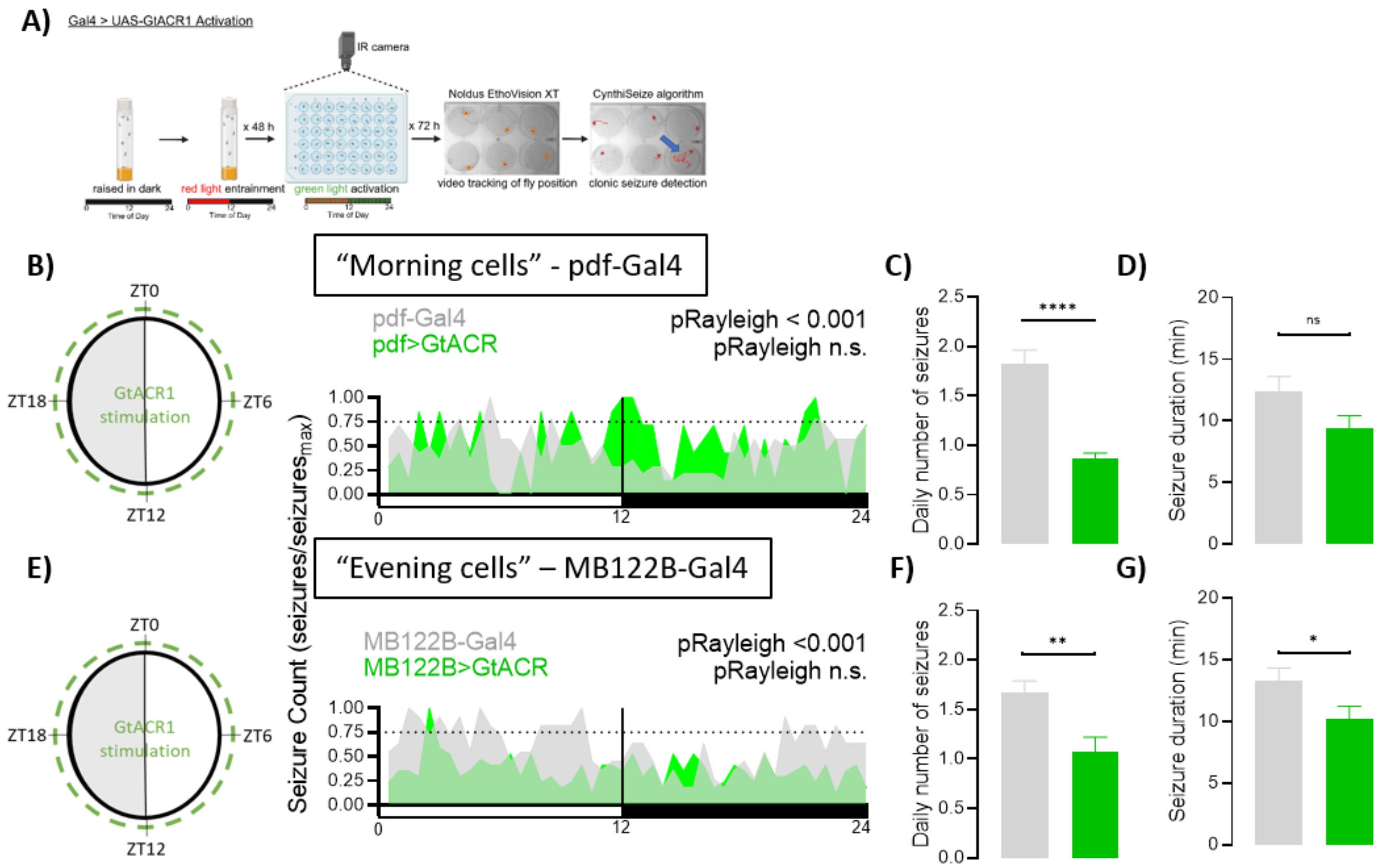

Supplementary Fig. 9 | Direct optogenetic inactivation of morning cells and evening cells changes seizure risk. a, Schematic depicting experimental protocol: flies are raised in the dark then entrained in red light for 48 hours. Flies are then placed into 48-well plates for GtACR1 stimulation with green light for 72 hours. Green light was switched on for 5 minutes and off for 25 minutes throughout the day. Noldus EthoVision XT was used for video tracking, and seizures were detected using the CynthiSeize algorithm. Created in BioRender. Sehgal, A. (2026) https://BioRender.com/9siy0zu **b,e** Clocks depict times of the day that green light was used to activate GtACR1. Inactivation of morning cells or evening cells throughout the day blunted seizure rhythms. n=68-96 flies/condition. "pRayleigh" indicates results of Rayleigh test. **c,f,** Inactivation of morning or evening cells throughout the day leads to a reduced number of seizures. n=172-284 seizures/condition. Negative binomial model with Wald test was used. **d,g,** Inactivation of evening, but not morning, cells throughout the day leads to a reduction in seizure duration. n=172-284 seizures/condition. A mixed effects model was used. *p<0.05 **p<0.01 ****p<0.0001. Data are presented as mean values ± SEM.

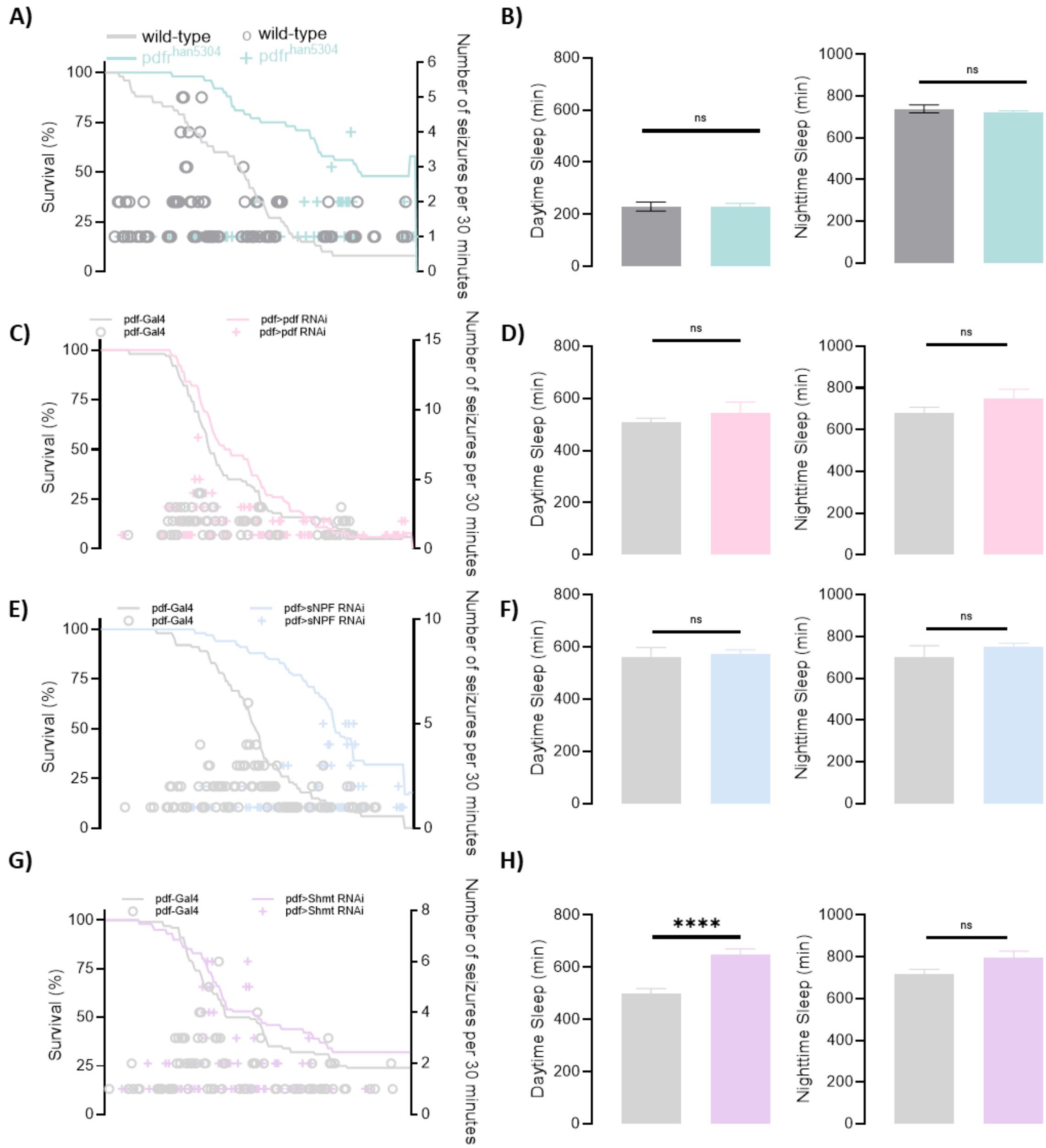

Supplementary Fig. 10 | Survival curves and sleep quantification after manipulation of *pdfr* and neurotransmitters/neuromodulators in *Pdf*+ morning cells. a,c,e,f, Survival curves and seizure timing. Survival enhancement was noted in *pdfr*^{han5304} mutants and after sNPF knockdown. **b,d,f,h,** Daytime and nighttime sleep quantification. Increased sleep was observed during the daytime after *Shmt* knockdown. n=48-68 flies/condition. Two-group two-tailed t-test. ****p<0.0001. Data are presented as mean values ± SEM.

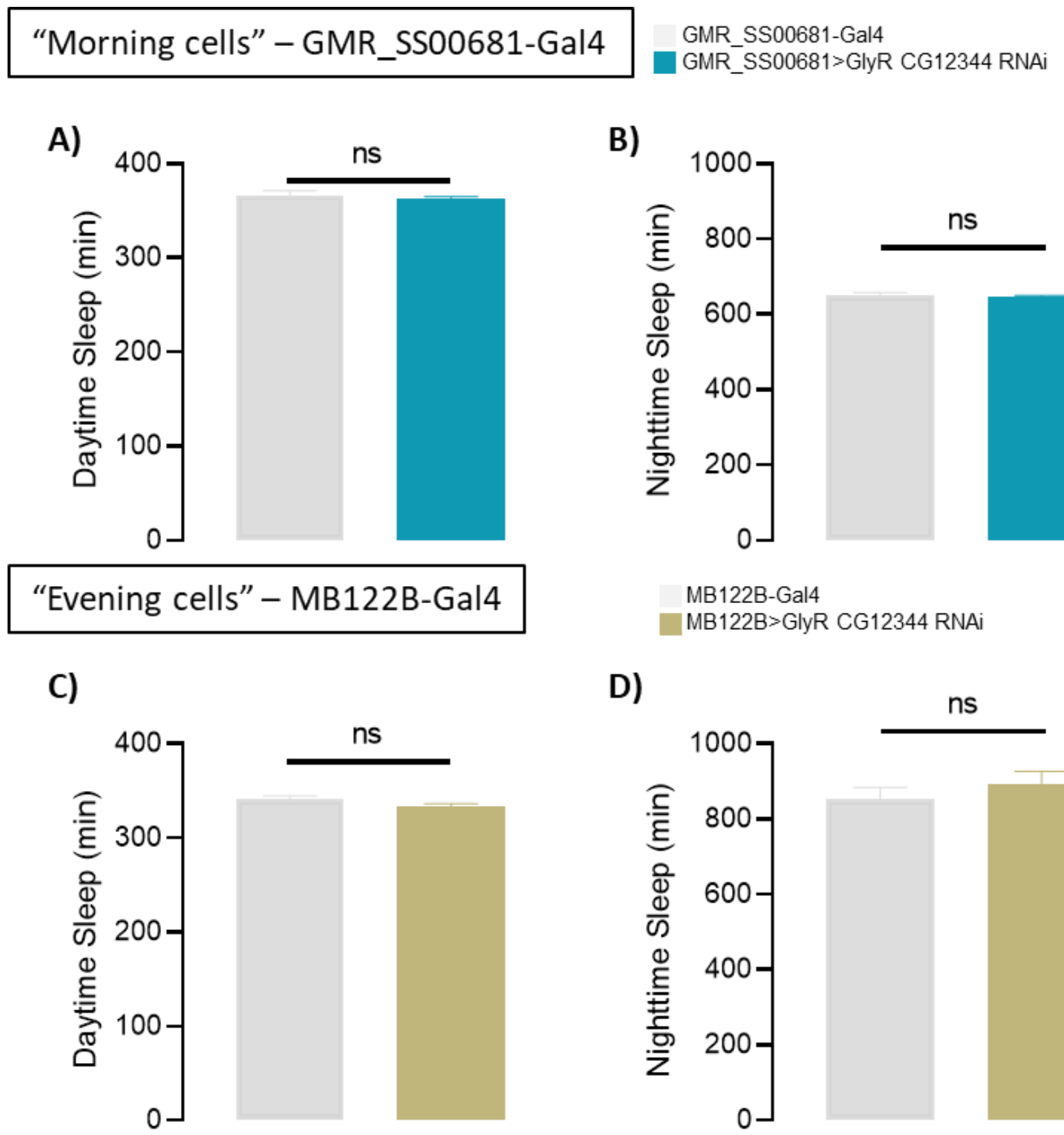

Supplementary Fig. 11 | Changes in sleep are not observed after knockdown of CG12344, the glycine receptor. a-d, No significant changes in daytime or nighttime sleep are noted after CG12344 knockdown in morning and evening cells. n=47-96 flies/condition. Two-group two-tailed t-test. Data are presented as mean values ± SEM.

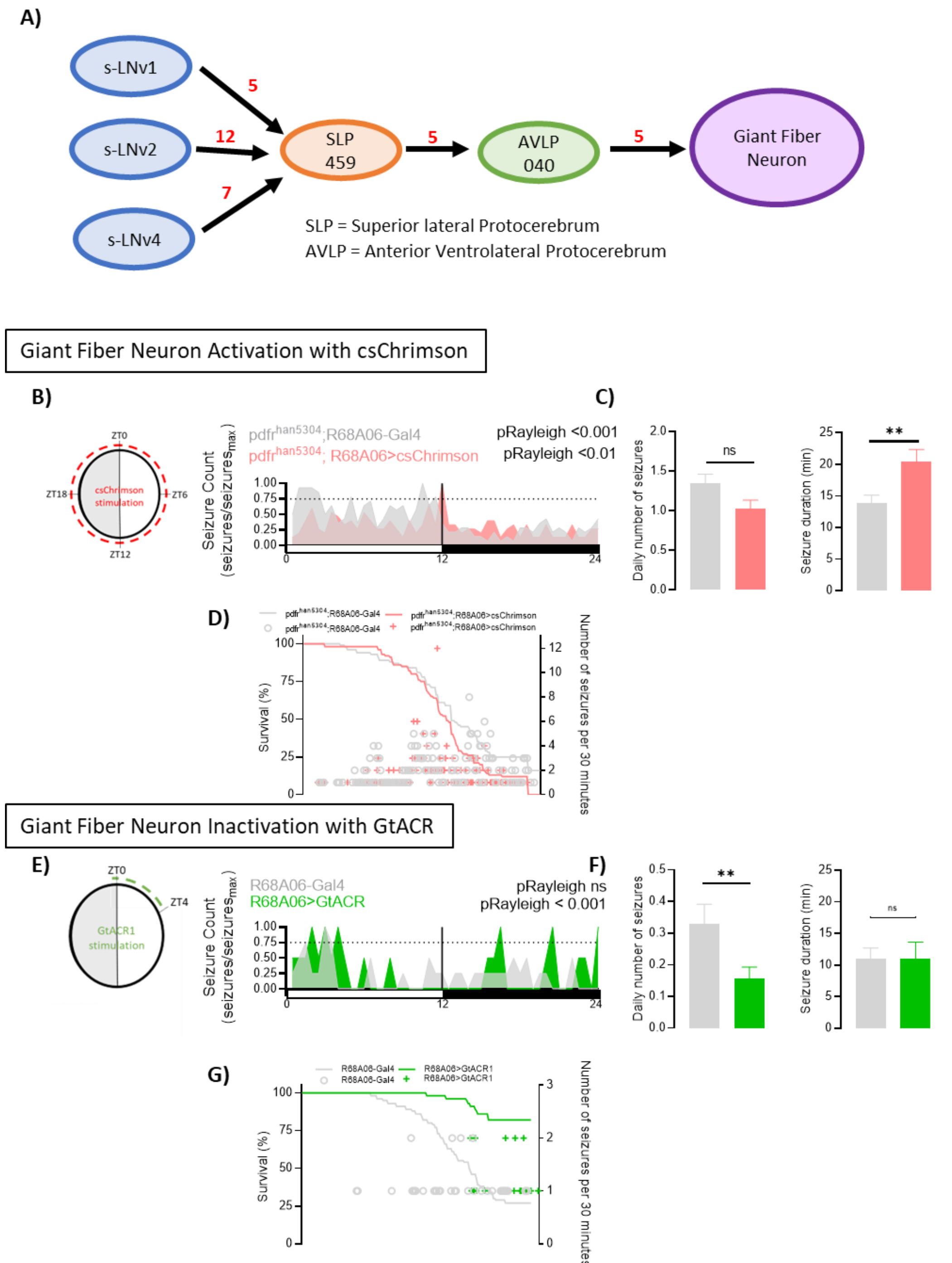

Supplementary Fig. 12 | Hemibrain connectome dataset reveals connections between morning cells and giant fiber neurons, which mediate seizures. a, The NeuPrint hemibrain connectome dataset[1,2] was used with a threshold of 5 synaptic connections. Most morning cells (s-LNv1, s-LNv2, and s-LNv4) are separated by 3 synapses from giant fiber neurons**. b,** Clock depicts around-the-clock timing of csChrimson stimulation. Seizures continue to occur predominantly in the daytime when csChrimson is activated in giant fiber neurons using the R68A006-Gal4 driver in a *pdfr^{han5304}* mutant background. "pRayleigh" indicates results of Rayleigh test. n=85 flies/condition. **c,** Seizure duration, but not seizure number, is significantly increased with giant fiber neuron activation. n=199-273 seizures/condition.

Negative binomial model with Wald test was used for seizure number. A mixed effects model was used for seizure duration. **d,** Survival plot demonstrates similar survival after csChrimson activation. n=85 flies/condition. **e,** Clock depicts GtACR1 stimulation from ZT0-4 with green light. n=56 flies/condition. **f,** Seizure number, but not seizure duration, is significantly decreased with giant fiber neuron inactivation. n=28-48 seizures/condition. Negative binomial model with Wald test was used for seizure number. A mixed effects model was used for seizure duration. **g,** Survival plot demonstrates enhanced survival after inactivation of giant fiber neurons. n=56 flies/condition. **p<0.01. Data are presented as mean values ± SEM.

Supplementary References

1. Plaza, S. M. *et al.* neuPrint: An open access tool for EM connectomics. *Front Neuroinform* **16**, 896292 (2022).
2. Scheffer, L. K. *et al.* A connectome and analysis of the adult Drosophila central brain. *Elife* **9**, e57443 (2020).
