# Supplementary material for: Clock neurons modulate seizure timing through a diurnal switch
Source: Nat Commun. 2026 Sep 30;17:11241. doi: 10.1038/s41467-026-77884-4 (PMC13720085; doi:10.1038/s41467-026-77884-4)
Supplement: Supplementary file 2 [file 41467_2026_77884_MOESM2_ESM.pdf]

Description of Additional Supplementary Files:

Supplementary Videos

Supplementary Video 1 │Sample video depicting induced bang-sensitive seizures in *tko*^{25t} mutant flies at 2 timepoints through the day. Four *tko*^{25t} flies were placed into each vial containing agar and sucrose. The first and second vials contain flies at ZT0, while the second and third vials contain flies at ZT12. Flies were given a strong mechanical stimulus using a vortexer prior to coming into video view. Seizures were quantified for atonic ("paralysis"), tonic/clonic ("convulsive"), and recovery ("postictal") phases.

Supplementary Video 2 │Sample video depicting a spontaneous seizure in wild type fly maintained in constant light. Wild type flies were placed into individual wells of a 48-well plate then maintained in constant light for a 96-hour continuous video recording. A spontaneous seizure was detected in well #46. Seizure manifests as hyperkinetic uncoordinated movements. *Red line* indicates fly position over previous 10 seconds.
