# Supplementary material for: Clock neurons modulate seizure timing through a diurnal switch
Source: Nat Commun. 2026 Sep 30;17:11241. doi: 10.1038/s41467-026-77884-4 (PMC13720085; doi:10.1038/s41467-026-77884-4)
Supplement: Supplementary file 3 [file 41467_2026_77884_MOESM5_ESM.pdf]

nature portfolio

Peer Review File

Clock neurons modulate seizure timing through a diurnal switch

Corresponding Author: Professor Amita Sehgal

This file contains all reviewer reports in order by version, followed by all author rebuttals in order by version.

Version 0:

Reviewer comments:

Reviewer #1

(Remarks to the Author)
In this manuscript, Cuddapah et al. present interesting findings on the mechanisms underlying day-night differences in seizure in Drosophila. Their use of Drosophila offers valuable insights into the cellular and molecular underpinnings of seizure rhythms. The incorporation of long-term video-tracking for seizure detection provides a robust approach to understanding seizure dynamics.

The identification of ventrolateral (morning cells) and dorsolateral (evening cells) neurons as drivers of diurnal oscillations in seizure is particularly noteworthy. Additionally, the involvement of pigment-dispersing factor (PDF), short neuropeptide F (sNPF), and glycine signaling adds depth to our understanding of seizure modulation in Drosophila.

While the study offers valuable insights, several critical aspects could be addressed to strengthen its scientific rigor and impact:
1. The authors could further differentiate the involvement of s-LNv and l-LNvs, as the latter are known as “arousal neurons” and not canonical clock neurons.
2. Glycine is a classic neurotransmitter with its release depending on neuronal activity. It is not straightforward how s-LNvs release glycine to inhibit E cells when s-LNvs exhibit low activity in the evening.
3. The authors report structural connections between clock neurons and giant fiber, but fail to demonstrate functional connections. Including functional connectivity through calcium imaging would strengthen the argument.
4. To entrain the flies with CsChrimson expression in clock neurons, it would be better to use temperature cycles but not light-dark (LD) cycles.
5. The differences between Drosophila and human epilepsy should be elaborated in the discussion. A more detailed comparison would provide context for the relevance and limitations of the findings.

Overall, this manuscript has important implications for understanding the temporal dynamics of seizures and developing targeted interventions. With the addition of further functional data and a more comprehensive discussion of the model's limitations, this study has the potential to be a highly impactful piece of research.

(Remarks on code availability)

Reviewer #2

(Remarks to the Author)
The study by Cuddapah et al addresses a potential role of the biological clock in regulating both seizure occurrence and, possibly, severity. The premise that seizure severity/frequency is regulated by the clock should mean that, regardless of seizure cause, all (most) seizure backgrounds would show clock-mediated rhythmicity. The issue here is that clock influences on human seizure are not robust and do not report consistent effects nor timings (e.g. https://pubmed.ncbi.nlm.nih.gov/33555292/) . Thus, it is difficult to accept the conclusion of a circadian influence when only one seizure mutant has been tested. Note also the timings of PTX-seizures shown in fig 1 (WT) seemingly differ from those reported for either pdf-GAL4 or MB122B-Gal4 (Fig 4, although all are highest in the mornings), and throughout the

manuscript. These are all essentially ‘wild type’ strains. It would greatly strengthen the study to include an additional seizure mutation (or two), possibly one with a more prevalent epilepsy-associated human orthologue (eg ParaBss, DS or GEFS+). This is an interesting study, but the conclusions and tone of the manuscript go beyond the data. I am not convinced that the authors have demonstrated a clear regulatory role for the clock.

Text and data relating toFig.2:
First, the figure legend needs better explanation for non-clock researchers. For example – are all the successive lines in the actograms the same and what is meant by the shading (D and G)? What is the black bar for in F (constant darkness?). The atonic phase in panel C is stated as $P<0.05$ – this value is hard to believe. Moreover, the phase of the atonic activity is out of phase with tonic/clonic activity? Again -better description in the text is needed.

Perhaps more troubling is a comparison between the tonic/clonic P value in panel C with that in panel F (the former being $P<0.001$ and the latter ns). The phase distributions are clearly different but the variation in troughs to peaks is about the same in both (~15 sec).

The splitting of seizure activity to different components in Fig 2 is unhelpful, unless also presented for the fly strains reported in Fig 1. However, I would suggest that Fig 2 include this type of ‘grouped data’ as shown for CS and tim flies in Fig 1 (panels 1B and 1D). The differential analysis/presentation between Figs 1 and 2 is not helpful and further undermines the conclusion that seizure occurrence is regulated by the clock.

The role of the clock in seizure could be strengthened by maintaining flies in either constant dark (free-running clock,) vs. constant light (non-functional clock) and testing for effect to seizure severity/frequency under these conditions.

Text and data relating to Fig.3:
The conclusion that acute activation of some clock neurons can result in increased seizure activity needs to be validated by expression on TrpA1 in other non-clock cell populations (of about the same number of cells). A different conclusion might be that activation of a sufficient number of neurons (of any type?) may be sufficient to affect seizure in a small CNS.

Text and data relating to Fig.5:
Daily number of seizures reported for pdf-Gal4 (panel C) vs. MB122B-Gal4 (panel F) are very different even though both are ‘controls’. This must be a concern because it implies that strain-specific genetics may be having as much an impact on seizure timing as reported clock manipulations. Are all lines cantonized (i.e. isogenic)? This should, at the least, be discussed. Also, why do the same GAL4 controls differ (pdf Gal4 in C vs. I for example?).

Text and data relating to Fig.6:
The effect of stimulating activity in morning cells at ZT0-4 (panel D) is marginal and would appear to be composed of two data subsets: 7 out of 12 showing an increase in Ca signal (i.e. activity) but 5 out of 12 showing no effect. This is not convincing and perhaps the approach to look at whole brain activity is overly simplistic? At the least this should be discussed in the relevant result section.

Text and data relating to Fig.7:
Again, there is deviation in the timing of seizures in the wild type shown in panel A/C compared to that shown in Fig.1 and elsewhere. This further underlines the criticism of definitive evidence to show seizure occurrence is ‘tightly’ regulated by the clock. Perhaps if the tone of the manuscript was altered to ‘indicative of influence’ these biological variations would be more acceptable.

Text and data relating to Fig.8:
The conclusion that morning cells act on evening cells via glycine release is not supported by this data. Sure, it is indicative, but no more.

Text and data relating to supp Fig.8:
Stimulation of the GF is known to induce seizure like activity. Thus, the fact that hyperactivation of this system increased seizure duration is perhaps not surprising. Moreover, as the GFS is one of the most significant brain to thoracic conduits, the effect of silencing this fibre is also not surprising. However, neither observation speaks to regulation by the clock. I do not feel that this section adds much to the study.

Discussion: Very much a rehash of the results and could easily be shortened.

General points:
The term ‘spontaneous seizure’ is used throughout the manuscript when in fact the seizures are induced by exposure to picrotoxin. Terminology to reflect this should be used. Indeed, a significant drawback to using flies is that they do not show truly spontaneous seizures (the few reports that suggest otherwise report only very modest seizure activity).

The introduction needs to go deeper into the literature reporting clock effects on seizure occurrence in humans. The field is confused, and no clear conclusions can be drawn. This does not come through on reading the intro.

Rose plots in some figures have too many colors (and pale colors at that) and are impossible to interpret.

What does FFT stand for (Fig 2E)?

XY plots in Fig 3 are not well explained

Methods state adult male flies were used, but in some expts (eg Fig 6) females are used. Why was this? Also, circadian influences on seizure might be expected to be similar in both sexes, but this has not been tested?

(Remarks on code availability)

Reviewer #3

(Remarks to the Author)
The manuscript employs a video-tracking method to investigate the timing susceptibility of induced seizures in Drosophila using genetics and pharmacological models of epilepsy. The work builds upon an extensive body of literature in humans and mammals demonstrating that seizures do not occur randomly throughout the day but follow a circadian rhythm and aims to create a genetically amenable model to study this phenomenon. The hypothesis is clear, and the manuscript is potentially interesting and impactful. However, there are some major issues with data interpretation and presentation that need to be addressed.

Figure 1A and Main Text: There is a critical issue regarding the algorithm, as it is neither explained nor validated within the paper. It is only referenced in a 2023 preprint that also does not adequately explain or validate the algorithm. This work relies heavily on this algorithm, making the methodology central and critical meaning it must be validated by demonstrating that it can detect seizures at least as reproducibly and reliably as the existing state of the art.

Figure 1B: The Y-axis is not annotated in this figure nor in other figures that use the same visualization techniques. This omission makes comparisons across conditions simply impossible.

Picrotoxin-Related Issues in Figure 1 and Other Figures: This is the main issue with this study. Picrotoxin induces seizures in Drosophila larvae within minutes of exposure. If I understand correctly, the flies are kept on this substrate for the duration of the experiment, meaning that seizures could occur after every feeding episode, which has a circadian component by itself. If that is the case, it is unsurprising that seizure probability is higher during the day than at night and is affected in circadian mutants. This represents a major weakness, as it could invalidate all experiments based on feeding. Feeding itself should be monitored, and the risk of seizures should be controlled and normalized over feeding patterns. Notably, the authors report that “No significant differences in the likelihood of seizure induction were noted in tko25t flies throughout the day (Supp. Fig. 1D)” and that “Again, the likelihood of seizure induction across groups did not change throughout the day (Supp. Fig. 1G)”, indicating that the circadian signal observed with picrotoxin feeding disappears when other proconvulsant models are used. This is one more reason why feeding control is absolutely crucial.

Fig1 D and conclusion 335-337: The observed deaths predominantly occurred in the early evening. While this finding is interesting, attributing it to sleep pressure (L335) is certainly premature.

Figure 2A: Additional details should be provided on how seizure quality was calculated. Line 106 states that the seizures are video recorded, but it is unclear whether the quantification is performed manually or by the algorithm.

Figure 2E/D/G: What are those yellow boxes? They are not labeled in the figure, legend, or text. Additionally, this figure would be completely cryptic to a reader who is not already extremely familiar with the model and the genotypes.

Figure 3: The same data are essentially presented in two different ways: with bar plots on the left and a different system on the right. Choose only one method, as there is no need to show the same information twice. The graph on the right is appealing but does not convey information about standard error and statistical comparisons in general. The legend states: “For unknown reasons, in tim-Gal4;tko25t flies, only 1 out of 96 flies tested exhibited a seizure so significance testing is not performed for this group.” This finding seems quite relevant and striking but is not discussed in the paper.

Fig. 7 and Supplementary Fig. 6: The authors show PDF and sNPF change seizures, while sleep is not affected. However, these findings may be inconsistent with previously studies. For instance, Chung et al Fig. 1C (https://www.sciencedirect.com/science/article/pii/S0960982209006125?via=ihub) reported that pdfrhan5304 showed increased sleep, and Shang et al Fig. 4A (https://www.sciencedirect.com/science/article/pii/S0896627313006533?via=ihub) found that knockdown of sNPF in pdf-Gal4 neurons decreased night sleep.

Figures 4/5/7/8: These figures suffer from the same issues as Figure 1 in terms of data presentation and the inability to draw conclusions related to picrotoxin feeding. May afraid it is simply premature to draw any conclusion until that major caveat is taken into account.

(Remarks on code availability)

Reviewer #4

(Remarks to the Author)
I co-reviewed this manuscript with one of the reviewers who provided the listed reports. This is part of the Nature Communications initiative to facilitate training in peer review and to provide appropriate recognition for Early Career Researchers who co-review manuscripts.

(Remarks on code availability)

Version 1:

Reviewer comments:

Reviewer #1

(Remarks to the Author)
The authors have thoroughly addressed all of my concerns. I have no further suggestions, and I congratulate them on a fine piece of work.

(Remarks on code availability)

Reviewer #2

(Remarks to the Author)
Whilst the revised manuscript has been improved, there are still some areas that require further elaboration/amending. These are:

1. Comparison of the effect of WT+PTX between tim(01) and LL shows obvious differences. The former shows no circadian influence (as expected) but the latter seemingly does (Raleigh $P<0.001$ which is similar to the WT, other than peak seizure count is shifted towards subjective night). Given that both conditions are expected to have a non-functional clock, why the difference?

2. The first discussion paragraph identifies 4 key findings of this study. However, point 1 is not novel. Other studies have shown a circadian influence on seizure in Drosophila, (https://pubmed.ncbi.nlm.nih.gov/41958013/). It is appropriate that previous studies are cited.

3. Whilst the authors make a strong case to maintain the section on the Giant Fiber, their conclusion that the results on this circuit have 'therapeutic potential' is questionable. Indeed, the penultimate paragraph of the Discussion points out the many limitations of using the fly for human epilepsy. The mention of 'therapeutic potential' should either be fully explained or deleted.

(Remarks on code availability)
outside of my expertise

Reviewer #3

(Remarks to the Author)
I thank the authors for their thorough and considered response to my original comments. The revision represents a substantial body of additional work, and I find that my concerns have been addressed convincingly. The methodological transparency around the seizure-detection pipeline is now resolved and I commend the authors for sharing the methods on Zenodo. My main concern — that the diurnal rhythm in picrotoxin-induced seizures might simply reflect rhythmic feeding — has been addressed through a multi-pronged argument that I find persuasive in aggregate.

Overall, this is a careful, technically demanding study that establishes Drosophila as a tractable system for dissecting the molecular and circuit basis of diurnal seizure rhythms. I am happy to recommend acceptance.

Giorgio Gilestro

(Remarks on code availability)

Reviewer #4

(Remarks to the Author)
I co-reviewed this manuscript with one of the reviewers who provided the listed reports. This is part of the Nature Communications initiative to facilitate training in peer review and to provide appropriate recognition for Early Career Researchers who co-review manuscripts.

(Remarks on code availability)

The authors have deposited a comprehensive "CynthiSeize" package on Zenodo. This includes a detailed PDF with step-by-step instructions and screenshots for every stage of the pipeline, from video acquisition in Ethovision XT to final data quantification in MATLAB.

Open Access This Peer Review File is licensed under a Creative Commons Attribution 4.0 International License, which permits use, sharing, adaptation, distribution and reproduction in any medium or format, as long as you give appropriate credit to the original author(s) and the source, provide a link to the Creative Commons license, and indicate if changes were made.
In cases where reviewers are anonymous, credit should be given to 'Anonymous Referee' and the source.
The images or other third party material in this Peer Review File are included in the article's Creative Commons license, unless indicated otherwise in a credit line to the material. If material is not included in the article's Creative Commons license and your intended use is not permitted by statutory regulation or exceeds the permitted use, you will need to obtain permission directly from the copyright holder.
To view a copy of this license, visit https://creativecommons.org/licenses/by/4.0/

REVIEWER COMMENTS

Reviewer #1 (Remarks to the Author):

In this manuscript, Cuddapah et al. present interesting findings on the mechanisms underlying day-night differences in seizure in Drosophila. Their use of Drosophila offers valuable insights into the cellular and molecular underpinnings of seizure rhythms. The incorporation of long-term video-tracking for seizure detection provides a robust approach to understanding seizure dynamics.

The identification of ventrolateral (morning cells) and dorsolateral (evening cells) neurons as drivers of diurnal oscillations in seizure is particularly noteworthy. Additionally, the involvement of pigment-dispersing factor (PDF), short neuropeptide F (sNPF), and glycine signaling adds depth to our understanding of seizure modulation in Drosophila.

While the study offers valuable insights, several critical aspects could be addressed to strengthen its scientific rigor and impact:
1. The authors could further differentiate the involvement of s-LNv and l-LNvs, as the latter are known as “arousal neurons” and not canonical clock neurons.

We thank the Reviewer for their encouraging feedback regarding the significance of our work, as well as this important suggestion. To distinguish the contributions of small versus large LNvs, we performed additional experiments and expressed *per* guide RNA and Cas9 in small LNvs selectively using the GMR-SS00681-Gal4 driver. This allowed us to selectively disrupt the clock in small LNvs and then assess the contribution to seizure timing. As pictured below (and now included as Supplementary Figure 6), clock disruption in small LNvs led to the emergence of a nighttime seizure peak. The rhythm was not completely lost, and the total number of seizures did not change. Together with Figure 4, these data are consistent with a model in which both small and large LNvs promote daytime seizures. These data also indicate that large LNvs play a more important role in controlling seizure number than small LNvs, as total seizure numbers dropped when *per* gRNA, Cas9 were expressed in small and large LNvs together using the *Pdf*-Gal4 driver (Fig. 4C) but not when *per* gRNA, Cas9 were expressed in small LNvs only (Supp. Fig. 6B). As suggested by the Taghert lab (Liang et al 2022), small and large LNvs may control downstream circuits at discrete morning and midday times, respectively. These additional data suggest both cell types play a role in promoting daytime seizure timing as well. The manuscript text has been updated accordingly.

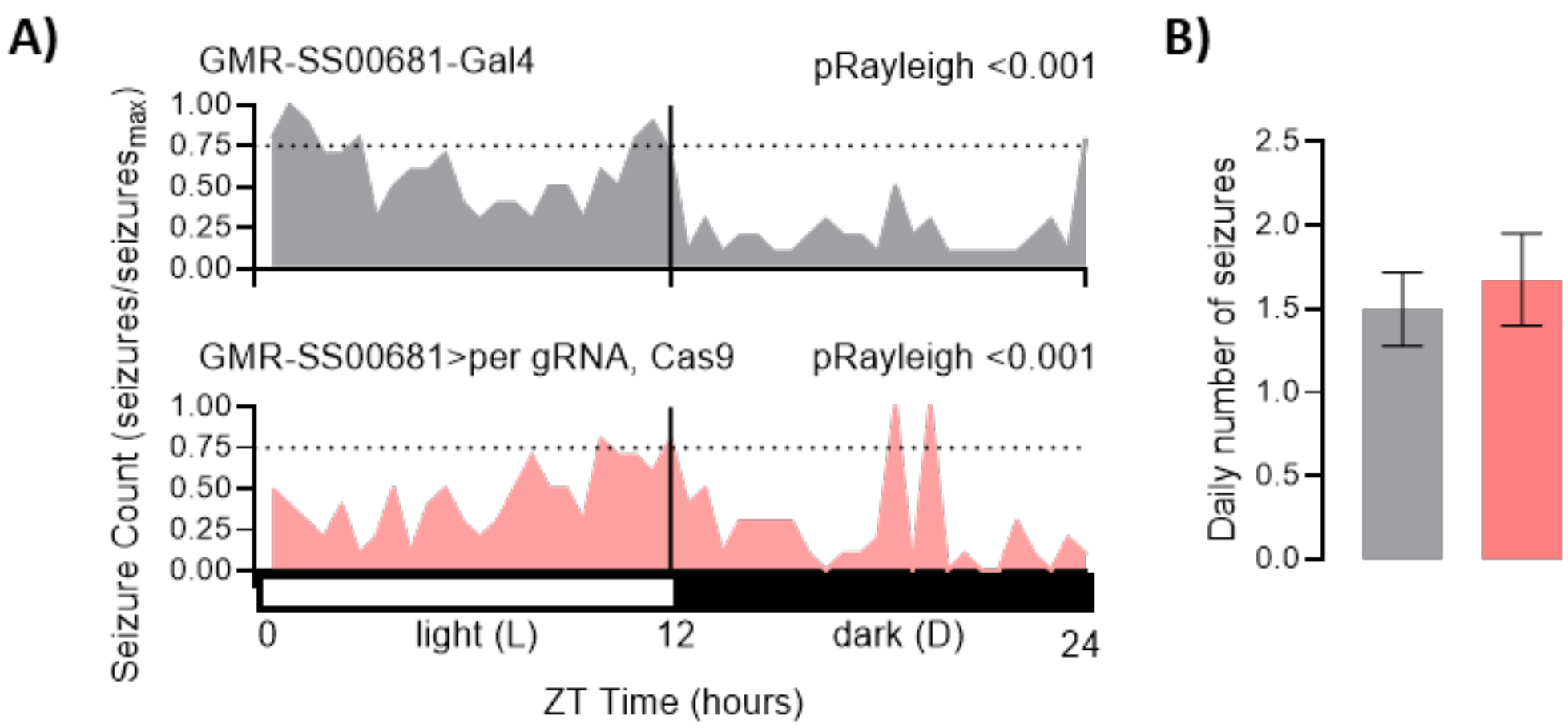

2. Glycine is a classic neurotransmitter with its release depending on neuronal activity. It is not straightforward how s-LNvs release glycine to inhibit E cells when s-LNvs exhibit low activity in the evening.

Small LNv activity oscillates with a peak in the dark-to-light transition and nadir at the light-to-dark transition. As the night progresses, small LNv activity ramps up, as seen in Ca^{2+} imaging data from the Taghert lab (Liang et al 2017). Our data suggest that during this ramping up of small LNv activity through the night, glycine acts on LNds to suppress nighttime seizures. Our model is consistent with one proposed by the Ceriani lab in which glycine is released from small LNvs tonically, but structural connections with LNds are greater at night, thereby enhancing the functional inhibition of small LNvs on LNds at night (Gorostiza et al 2014; Frenkel et al 2017). Despite an overall decrease in synapse number, the number of small LNv synapses through which fast neurotransmission occurs (including glycine) remains high at about 30 presynaptic sites per neuron at night (Ispizua et al 2025).

The Discussion has been modified as follows: "As the night progresses, morning cell activity ramps up[43] and direct synaptic contact with evening cells increases[41,44,45]. Therefore, our data suggest that during this ramping up of morning cell activity through the night, glycine can act on evening cells to suppress nighttime seizures."

3. The authors report structural connections between clock neurons and giant fiber but fail to demonstrate functional connections. Including functional connectivity through calcium imaging would strengthen the argument.

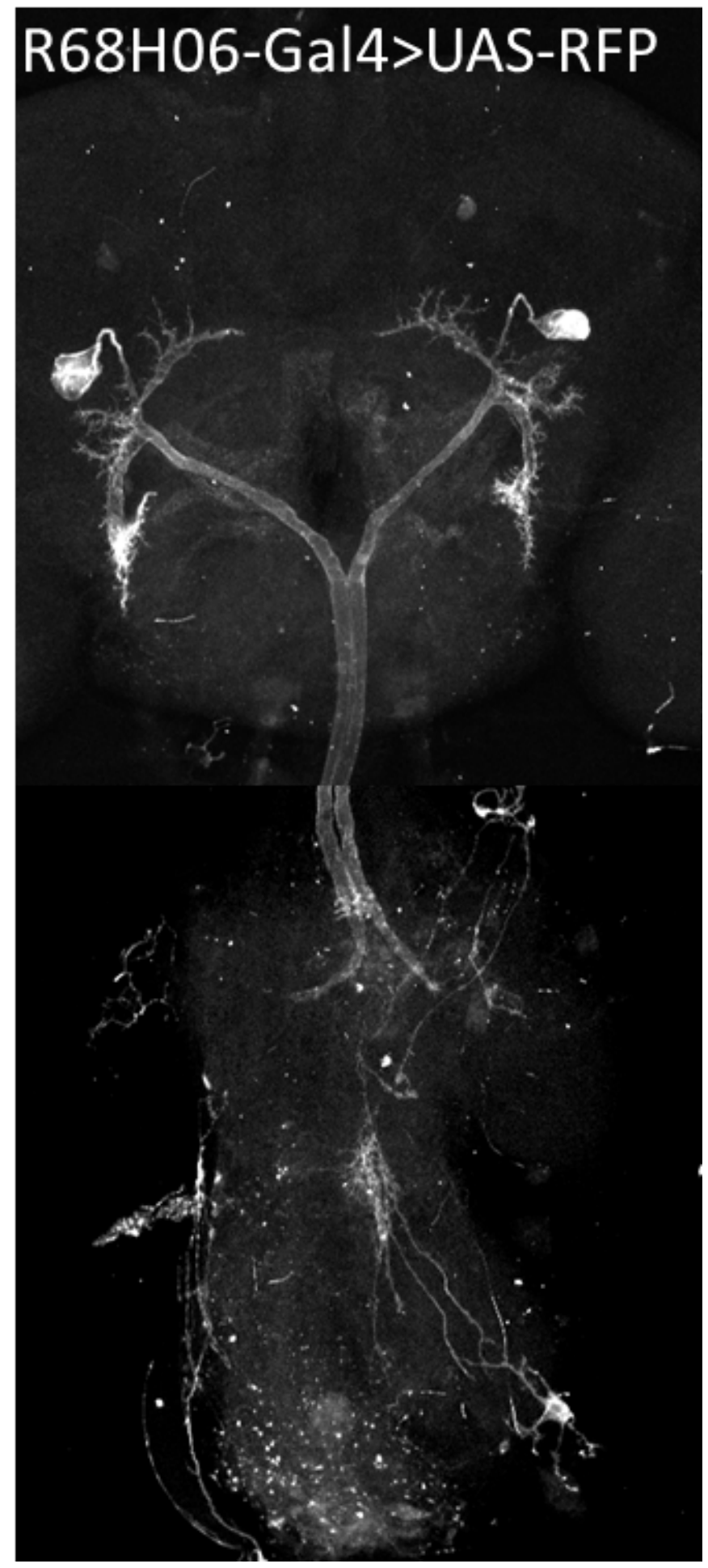

We appreciate the Reviewer's insightful suggestion and agree that functional connectivity data would complement the structural findings in Supp Fig. 12A. To address this, we conducted extensive calcium imaging experiments in the giant fiber (GF) neurons using the CaLexA system, mirroring our approach in Figure 6B.

We utilized two distinct GF-targeting drivers (R68H06-Gal4 and R17A04-Gal4). Despite optimization, we were unable to detect a consistent calcium signal. To rule out axotomy-induced injury to the large-diameter descending axons as a confounding factor, we performed imaging on both isolated brains and brains with intact ventral nerve cords. We evaluated both endogenous fluorescence and anti-GFP immunohistochemistry to maximize detection sensitivity; however, neither approach yielded a CaLexA signal.

Importantly, we confirmed the efficiency and specificity of both drivers by expressing RFP, which resulted in robust labeling of the GF neurons and their VNC projections (pictured to the right).

Through these multiple attempts, we find the CaLexA system is technically limited in its application to GF neurons. This may be due to the massive cytoplasmic volume of GF neurons diluting the CaLexA reporter molecules below the threshold of detection, and/or unique intracellular calcium buffering dynamics in these giant descending neurons that are insufficient to drive the NFAT-based transcription required by the CaLexA system.

In the Discussion this is indicated as a limitation with the following text: "Here, beyond noting the structural connections as identified by electron microscopy (Supp. Fig. 12A), we do not explore how clock neurons and giant fiber neurons are functionally connected due to technical limitations in calcium imaging of giant fiber neurons."

4. To entrain the flies with CsChrimson expression in clock neurons, it would be better to use temperature cycles but not light-dark (LD) cycles.

For the CsChrimson experiments, we entrained flies in blue light. Blue light penetration of the *Drosophila* cuticle is poor (Inagaki et al 2013) but can be used to entrain flies (Stanewsky et al 1998). Therefore, circadian entrainment with blue light has emerged as a popular strategy (Sitaraman et al 2024, Ni et al 2019). Temperature itself can have an impact on seizure likelihood (Burg and Wu 2012), so we avoided temperature manipulation when possible. The text has been edited as follows to make it clearer that we used blue light entrainment:

"Blue light was used to entrain flies while limiting excitation of csChrimson through the cuticle[25–28]."

5. The differences between Drosophila and human epilepsy should be elaborated in the discussion. A more detailed comparison would provide context for the relevance and limitations of the findings.

We thank the Reviewer for highlighting this point and now further emphasize this in the Discussion. We now explicitly discuss the anatomical and physiological differences between seizures in *Drosophila* and people, while reiterating that the highly conserved molecular pathways in *Drosophila* make it a valuable tool for mechanistic discovery.

"While the fly model offers valuable insights into mechanisms controlling seizure timing, translating these findings to human epilepsies presents inherent limitations. The mammalian brain utilizes complex networks across billions of neurons of various subtypes. In contrast, the fly brain contains approximately 140,000 neurons and lacks a cerebral cortex, which is the primary seat of seizures in humans. The specific brain regions driving fly seizures, beyond the giant fiber system, remain unmapped. Seizures in people are defined by both stereotyped behavioral and electrographic features. Because obtaining electrical access in freely moving flies is technically challenging, fly seizures have been predominantly described using behavioral readouts. Consequently, seizures that do not manifest as sustained tonic-clonic movements are likely undetected. Nevertheless, the fundamental molecular mechanisms governing neuronal excitability are highly conserved between flies and humans. This includes voltage-gated ion channels, synaptic release machinery, and molecular clock machinery. Despite its anatomical simplicity, the fly model not only exhibits seizures but also demonstrates daily rhythms in seizure susceptibility, providing a powerful reductionist system for identifying conserved seizure triggers."

Overall, this manuscript has important implications for understanding the temporal dynamics of seizures and developing targeted interventions. With the addition of further functional data and a more comprehensive discussion of the model's limitations, this study has the potential to be a highly impactful piece of research.

We thank the Reviewer for recognizing the value of this work.

Reviewer #2 (Remarks to the Author):

The study by Cuddapah et al addresses a potential role of the biological clock in regulating both seizure occurrence and, possibly, severity. The premise that seizure severity/frequency is regulated by the clock should mean that, regardless of seizure cause, all (most) seizure backgrounds would show clock-mediated rhythmicity. The issue here is that clock influences on human seizure are not robust and do not report consistent effects nor timings (e.g. https://pubmed.ncbi.nlm.nih.gov/33555292/) . Thus, it is difficult to accept the conclusion of a circadian influence when only one seizure mutant has been tested. Note also the timings of PTX-seizures shown in fig 1 (WT) seemingly differ from those reported for either pdf-GAL4 or MB122B-Gal4 (Fig 4, although all are highest in the mornings), and throughout the manuscript. These are all essentially 'wild type' strains. It would greatly strengthen the study to include an

additional seizure mutation (or two), possibly one with a more prevalent epilepsy-associated human orthologue (eg ParaBss, DS or GEFS+). This is an interesting study, but the conclusions and tone of the manuscript go beyond the data. I am not convinced that the authors have demonstrated a clear regulatory role for the clock.

We sincerely thank the Reviewer for their critical assessment of our manuscript and for the constructive suggestions; we have extensively revised the manuscript to incorporate each of these comments. (a) We provide a more detailed introduction to diurnal seizure rhythms in people with epilepsy and emphasize that while the specific timing of seizures is not the same in all people and etiology-specific, more than 80% of people with epilepsy exhibit daily fluctuations in seizure risk. (b) Following the Reviewer's advice, in addition to the genetic (tko^{25t}) and pharmacological (picrotoxin) models we initially presented, we have generated new data using two additional seizure models (DS and GEFS+ temperature-sensitive seizures); these models also exhibit daily rhythms in seizure risk and are now included in the manuscript. (c) Though daytime seizures are preserved across genetic controls (as the Reviewer states), we now acknowledge differences in timing of seizures during the daytime in control genotypes. (d) Where appropriate, we have tempered the tone of the manuscript, including the title, to not overstate our findings.

(a) Daily rhythms in seizure occurrence versus timing of seizure peaks in people with epilepsy:

The Reviewer rightfully points out that human clinical data, including the referenced study (PMID: 33555292), shows considerable variability regarding the specific timing of seizures across different patients. We completely agree. However, we would like to emphasize the distinction between (i) the phase of peak seizure timing, versus (ii) the presence of rhythmicity. While the specific time of day a seizure is most likely to occur varies between individuals, likely due to etiology-specific considerations, the presence of a diurnal rhythm in seizure risk is remarkably widespread. Multiple studies demonstrate that more than 80% of people with epilepsy exhibit significant diurnal rhythmicity in their seizure occurrence. In the referenced study (PMID: 33555292), 89% of individuals experienced diurnal oscillation in seizures.

Thus, this distinction of seizure *rhythms* being highly prevalent in people with epilepsy with a *phase* that is variable was not detailed. We have now added the following to the Introduction: “While more than 80% of people with epilepsy exhibit diurnal oscillations in seizure risk, the exact time of day that seizure risk peaks varies from person to person and relates to underlying genetic and structural etiologies[2–5]. Across all etiologies, the peak time for seizure occurrence on average is around the early- to mid-day, though across individuals, seizure peaks can occur at any time of the day [1,4,6]. Interictal epileptiform discharges also exhibit diurnal rhythms as revealed by prolonged recordings from implanted brain stimulation devices[2]. The phase of peak seizure timing is frequently tied to underlying brain regions involved, as well as genetic etiologies[5,6].”

These points have also been emphasized in the Discussion in the following revised paragraph: “The molecular clock does not drive a single “seizure time” for all epilepsies. Instead, the molecular clock likely drives rhythms in physiology that interact with distinct genetic and structural etiologies to produce individualized peak seizure times. For example, for the picrotoxin-induced seizures and bang-sensitive seizures in tko^{25t} mutants we study here, seizure severity was greatest during the daytime, but in $para^{GEFS+}$ and $para^{DS}$ flies, temperature-sensitive seizures clustered at the day-to-night transition. In people with epilepsy, sleep-related

hypermotor epilepsy is typified by nighttime seizures, and pediatric focal seizures arising from the occipital lobe can arise in the early evening[5]. These nighttime predominant seizures might result from regulation by other clock neurons, e.g. LNds, that are predominantly active in the evening[43,57]. Just as the multiple circadian phases of downstream non-clock neurons are set by various clusters of clock neurons[57], the phase-shifting of seizure risk might be predominantly controlled by these same subclasses of clock neurons. Alternatively, genes implicated in monogenic epilepsies, including *SCN1A* (orthologous to the *paralyic* gene in flies we study here), *KCND2*[58], and *KCNA4*[59], also control firing of neurons in the suprachiasmatic nucleus[60,61], which might alter the phase of seizure timing."

(b) Inclusion of additional seizure models (DS and GEFS+):

We are grateful for the suggestion to test additional, clinically relevant epilepsy models. We have performed new experiments utilizing established *Drosophila* models for Dravet Syndrome (DS) and Genetic Epilepsy with Febrile Seizures Plus (GEFS+). $para^{GEFS+}$ and $para^{DS}$ flies exhibit temperature-sensitive seizures and were maintained at 30°C for four days.

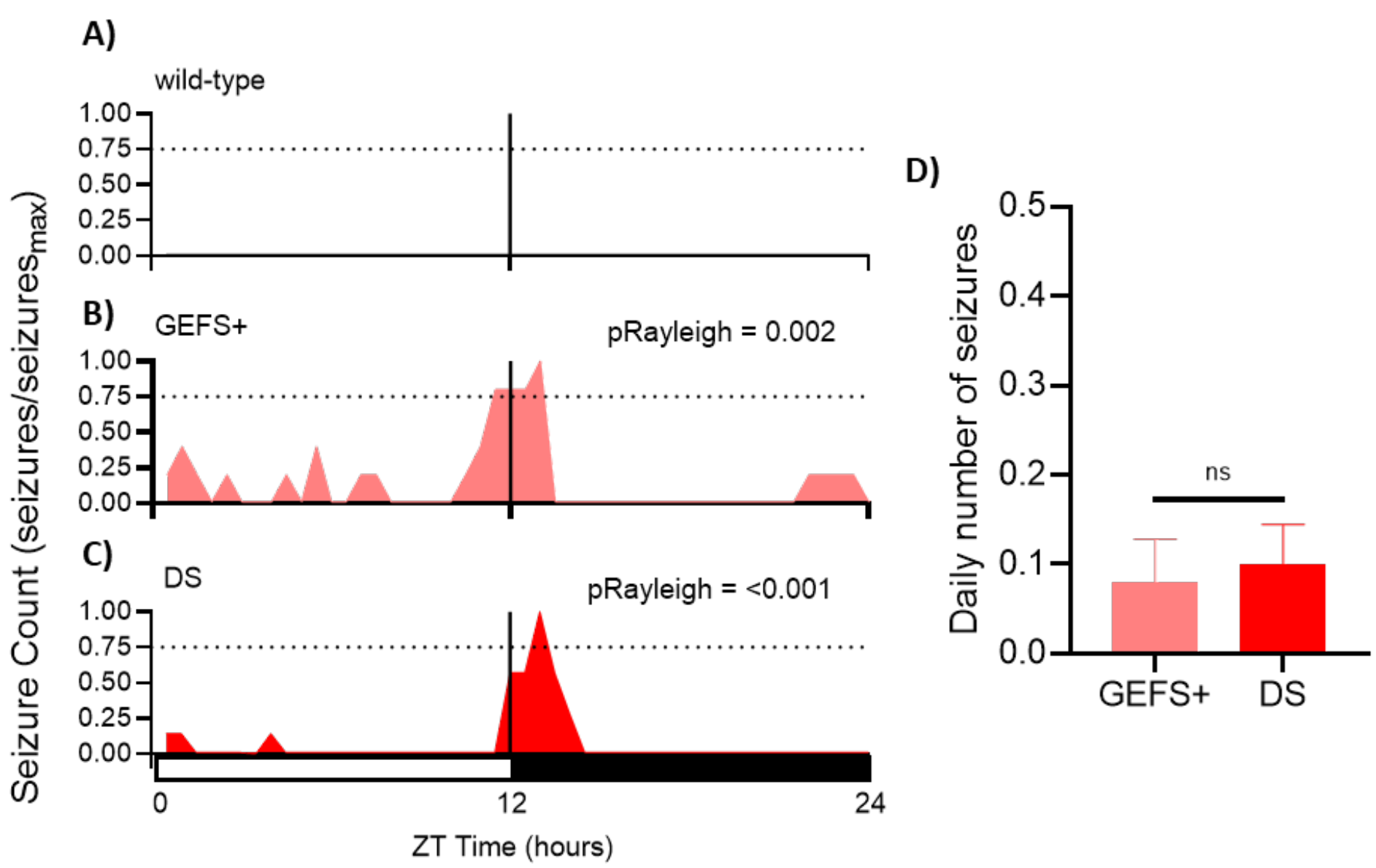

As now shown in Supp. Fig 4, both the DS and GEFS+ models exhibit robust diurnal oscillations in seizure susceptibility. The timing of peak seizure susceptibility differs between these new models and the tko^{25t} mutants and picrotoxin-induced model. This variability in peak seizure timing reflects the clinical reality discussed above: peak times of seizure risk are present across epilepsy etiologies, but the specific timing of peak vulnerability is unique to the underlying etiology. We believe this new data strengthens our findings that diurnal oscillations in seizure risk are common, and through incorporation of the Reviewer's constructive comments, we also now point out the timing of seizure peaks may vary. The investigation into daytime seizures we undertake in this study provides a baseline model of how the molecular clock and clock neurons can control seizure timing.

The Results have been edited as follows: "We also tested two clinically relevant models of epilepsy: $para^{GEFS+}$ and $para^{DS}$ [18,19]. These flies contain knock-in mutations in *paralytic*, the fly ortholog of the human *SCN1A*, which is implicated in Genetic Epilepsy with Febrile Seizures

Plus (GEFS+) and Dravet Syndrome. Incubation of flies at 30°C revealed a diurnal seizure rhythm with a peak occurring at the day-to-night transition (Supp. Fig 4). Thus, as seen in people with epilepsy, rhythms in seizure severity are widespread, occurring across the 3 genetic models and 1 pharmacological model we studied here, with the phase of the seizure peak being etiology-specific."

As noted above, the Discussion has been edited to incorporate these new findings.

(c) Variability in daytime seizures between genetic controls:

As the Reviewer notes, while picrotoxin-induced seizures are consistently most severe in the daytime, there is variability in when during the daytime a seizure peak occurs. For example, while seizures peak in the midday in Fig. 1B (wild-type Canton S) and Fig. 4A (pdf-Gal4), seizures peak in the early day and late day in Fig. 1E (MB122B-Gal4) and Supp. Fig. 6A (GMR-SS00681-Gal4). We are stringent about ensuring each experiment is performed on the same genetic background, allowing genetic controls to be compared to experimental animals. It is important to note that the seizure behavior we study here are not a typical function of clock neurons, but instead a pathological behavior modulated by clock neurons. Therefore, biological variance is expected.

We have added the following acknowledging this variance, while highlighting that the overarching pattern of daytime predominance in seizures remains intact across control groups: "We note that among control genotypes, daytime seizures varied from midday (Fig. 1B and Fig. 4A) to early and late in the day (Supp. Fig 6A)."

(d) Tempering the tone:

We have taken the Reviewer's guidance to heart and revised the manuscript to temper our language. The title has been changed from "Clock neurons time seizures through a diurnal switch" to "Clock neurons modulate seizure timing through a diurnal switch." We now do a better job of framing clock neurons as a "modulator" of seizure rhythms rather than the primary driver. As suggested and noted above, we now explicitly discuss the limitations of extrapolating our findings across all seizure etiologies. Examples include:

- Abstract: "Our findings reveal an unexpected role for brain clock neurons in controlling daily seizure risk." → "Our findings reveal an unexpected role for brain clock neurons in modulating daily seizure risk."
- Abstract: "At night, morning cells switch to glycine signaling acting on evening cells to suppress seizures." → "At night, morning cells switch to glycine signaling likely acting on evening cells to decrease seizure risk."

Text and data relating to Fig.2:
First, the figure legend needs better explanation for non-clock researchers. For example – are all the successive lines in the actograms the same and what is meant by the shading (D and G)? What is the black bar for in F (constant darkness?). The atonic phase in panel C is stated as $P<0.05$ – this value is hard to believe. Moreover, the phase of the atonic activity is out of phase with tonic/clonic activity? Again -better description in the text is needed.

We thank the Reviewer for this constructive criticism. The Fig. 2 legend has been updated as follows: "**a,** Experimental overview showing 6 timepoints at which bang-sensitive seizures were

induced using a table-top vortexor. Seizures were then video recorded to quantify the atonic, tonic/clonic, and recovery phases. **b-c,** Duration of seizures or seizure components in tko^{25t} flies across the day. JTK cycle, a test for rhythmicity, was used and p-value is provided in the Figure as pCycle. n=12-22 seizures/timepoint. **d,g,** Representative activity records in which vertical lines indicate activity. The rows represent successive days over which activity was monitored. Yellow shading indicates light for a 12-hour light:12-hour dark period. **e,h,** Fast Fourier Transform **(**FFT) calculations. n=27-48 flies/condition. One-way ANOVA with Tukey's multiple comparisons test was used. **f,** Seizure duration in tko^{25t};tim^{01} double-mutants show no rhythms through the day in constant darkness, as indicated by the *black* bar. n=6-41 seizures/timepoint. JTK cycle was used to test rhythmicity, and p-value is provided in the Figure as pCycle. **i-j,** Rhythms of seizure duration are suppressed after perturbation of the molecular clock. n=12-37 seizures/timepoint. JTK cycle was used to test rhythmicity, and p-value is provided in the Figure as pCycle. ****p<0.0001. Data are presented as mean values ± SEM."

In Fig. 2C, the Reviewer brings attention to the cycle identified in the atonic phase, which has a pCycle <0.05 as calculated by JTK cycle. We would like to emphasize here that while the amplitude of the rhythm is very low, a rhythm is present. As the Reviewer indicates, the phase of this weak rhythm is not aligned with the tonic clonic phase. This is addressed in the Results section as, "This oscillation in seizure duration was predominantly driven by a strong cycle in tonic-clonic durations, and to a lesser extent, by a weak cycle in the atonic phase (Fig. 2C)."

Perhaps more troubling is a comparison between the tonic/clonic P value in panel C with that in panel F (the former being P<0.001 and the latter ns). The phase distributions are clearly different but the variation in troughs to peaks is about the same in both (~15 sec).

In the revised Fig. 2 legend as noted above, we make it clearer that the p-values provided in Fig. 2B,C,F,I,J as "pCycle" are calculated by JTK cycle. JTK cycle is an algorithm used to identify rhythms in time-series data. It is sensitive to detecting rhythms with low amplitudes and does this by comparing datapoints to reference cosine waveforms. Thus, as the Review notes, while the amplitudes of change are comparable in Fig. 2C vs Fig. 2F, the datapoints in Fig. 2F do not fit to a 24-hour rhythm, and therefore the pCycle is n.s. To make this clearer, we have revised the results section as follows: "tko^{25t}; tim^{01} flies exhibited no 24-hour rhythms in seizure duration with emergence of a possible ultradian rhythm (Fig. 2F, Supp. Fig. 1H)."

The splitting of seizure activity to different components in Fig 2 is unhelpful, unless also presented for the fly strains reported in Fig 1. However, I would suggest that Fig 2 include this type of 'grouped data' as shown for CS and tim flies in Fig 1 (panels 1B and 1D). The differential analysis/presentation between Figs 1 and 2 is not helpful and further undermines the conclusion that seizure occurrence is regulated by the clock.

The spontaneous tonic clonic seizures detected through continuous video tracking in Fig. 1 are detected though an experimental approach that is methodologically different from the induced bang-sensitive seizures in Fig. 2. In Fig. 1, as depicted in Fig. 1A, flies are video recorded for 4 days, and through our automated analysis pipeline, we detect tonic clonic seizures. Using video tracking as in Fig. 1, we cannot distinguish the atonic phase and recovery phase from normal fly behaviors, if these phases even exist for picrotoxin-induced seizures (this is currently unknown). In contrast, for induced bang-sensitive seizures, we distinguish discrete the classical atonic, tonic clonic, and recovery phases as depicted in Fig. 2A. We find this information helpful to provide

for induced seizures as it shows that a specific phase, the tonic clonic phase, drives the rhythm in seizure severity seen in *tko^25t^* mutant flies.

The data in Fig. 1B and 1D reflect seizures that are detected on a continuous timeline through the 24-hour day, but the data in Fig. 2 are from 6 discrete timepoints in the day, because bang-sensitive seizures cannot be induced on a continuous timeline. Therefore, we are unable to represent the data in the same manner.

Despite seizures occurring using orthogonal approaches, we find that the worsening of daytime seizures is clock-dependent.

We now add the following sentence to the Results section to make it clear that we cannot quantify subphases of seizures for the video-tracking experiments: "This identification of seizure phases was not possible using our video tracking-based approach in Fig. 1A."

The role of the clock in seizure could be strengthened by maintaining flies in either constant dark (free-running clock,) vs. constant light (non-functional clock) and testing for effect to seizure severity/frequency under these conditions.

We appreciate the Reviewer's recommendation and performed these experiments. We find that daytime predominant seizures persist in constant darkness, though as expected for other circadian processes, the rhythm is dampened (PMID: 18840359). Additionally, in constant light, time-of-day differences were dampened and there was a shifting in phase to a peak in nighttime seizures, confirming circadian regulation of seizures but also indicating homeostatic control of seizures, likely associated with decreased sleep (PMID: 40730814). Surprisingly in constant light, we detected a seizure in a wild-type fly, demonstrating the toxicity of constant light exposure. These data are now included in Supp. Fig. 2 (also below) and the seizure in a wild-type fly in constant light is presented in Supp. Video 2.

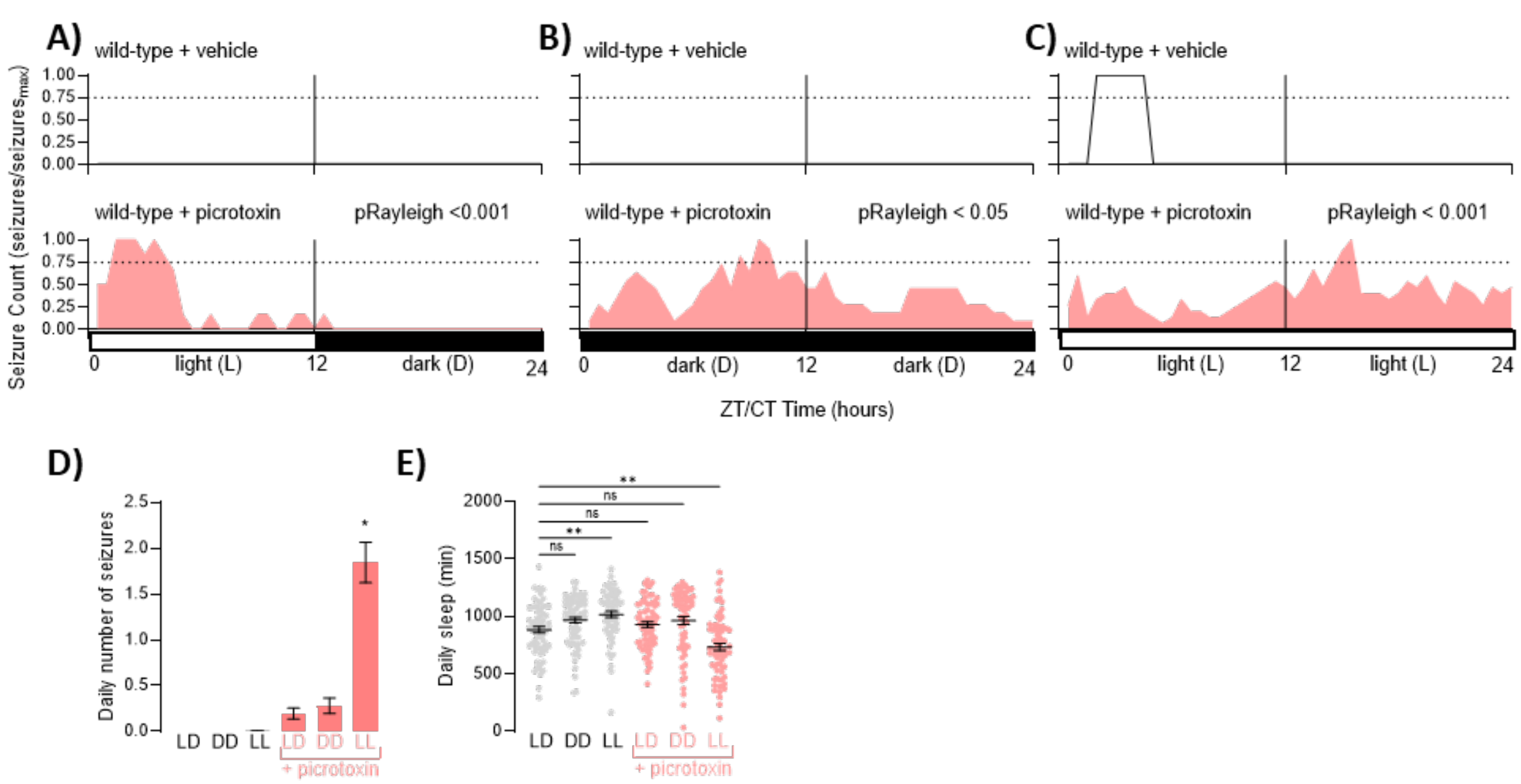

The Results section has been updated as follows: "These daytime predominant seizures persisted in constant darkness, though as seen for other circadian processes[16], the rhythm is dampened

(Supp. Fig. 2A-B). In constant light, diurnal differences in seizures were dampened, and there was a shift in phase to a nighttime peak in seizures, confirming circadian regulation of seizures but also indicating homeostatic control of seizures, likely associated with decreased sleep[13] (Supp. Fig. 2C, E). Surprisingly in constant light, we detected a seizure in a wild-type fly and a six-fold increase in picrotoxin-induced seizures, reflecting the toxicity of constant light exposure (Supp. Fig. 2D; Supp. Video 2)."

Text and data relating to Fig.3:
The conclusion that acute activation of some clock neurons can result in increased seizure activity needs to be validated by expression on TrpA1 in other non-clock cell populations (of about the same number of cells). A different conclusion might be that activation of a sufficient number of neurons (of any type?) may be sufficient to affect seizure in a small CNS.

Our data presented here suggest it is the type of neuron, not the total number of neurons, that is important in modulating seizure severity. As depicted in Fig. 3, driving activation of neurons labeled by *cry*-Gal4 (targeting LNvs, LNds, most DN1 cells, two DN2 cells, a fraction of DN3 cells, and some other neuronal groups that do not express clock genes), *pdf*-Gal4 (targeting 4 small and 4 large LNvs per hemisphere), R65D05-Gal4 (targeting 3 LPN neurons per hemisphere), and MB122B-Gal4 (targeting 6 LNd neurons per hemisphere) led to seizure exacerbation. However, activation with Clk856-Gal4 (targeting 60-75 clock neurons per hemisphere), and R18H11-Gal4 (targeting 16 DN1s per hemisphere), did not exacerbate seizures. Thus, there does not appear to be a correlation between the number of neurons targeted and the likelihood of seizure exacerbation.

In addition, our recently published study using an identical assay (PMID: 40730814) also support the conclusion that accounting for neuronal identity, and not neuronal number, is more important in toggling seizure severity. For example, activation of broad subsets of neurons including peptidergic (C929-Gal4), serotonergic/dopaminergic (Ddc-Gal4), octopaminergic (Tdc2-Gal4), and dopaminergic (TH-Gal4) neurons did not alter seizure severity.

Text and data relating to Fig.5:
Daily number of seizures reported for pdf-Gal4 (panel C) vs. MB122B-Gal4 (panel F) are very different even though both are 'controls'. This must be a concern because it implies that strain-specific genetics may be having as much an impact on seizure timing as reported clock manipulations. Are all lines cantonized (i.e. isogenic)? This should, at the least, be discussed. Also, why do the same GAL4 controls differ (pdf Gal4 in C vs. I for example?).

When we activated P*df*+ neurons around the clock with csChrimson initially, we saw a large increase in seizures that led to lethality. Therefore, we decreased the concentration of picrotoxin by 10-fold and repeated the experiment to avoid a ceiling effect and better assess increases in seizure severity. This is why the number of seizures between the initial control group is different from the following control groups. In the initial manuscript, this was noted in the Results section as "We noted that a high seizure density was associated with lethality (Supp. Fig 1B), so we reduced the concentration of picrotoxin by 10-fold to avoid lethality and improve our ability to see increases in seizure numbers."

To improve clarity, we have (a) changed the position of these sentences in the Results section to make it clearer that this pertains to the first *Pdf*-Gal4 experiment presented in the new Fig. 5B-C

and (b) added the phrase "using the higher picrotoxin concentration" when discussing the following experiments. This was also indicated in the initial Methods section.

We agree with the Reviewer that genetic background can impact behavior. We are very careful to ensure that the genetic background is consistent between control and experimental conditions in each experiment.

Text and data relating to Fig.6:
The effect of stimulating activity in morning cells at ZT0-4 (panel D) is marginal and would appear to be composed of two data subsets: 7 out of 12 showing an increase in Ca signal (i.e. activity) but 5 out of 12 showing no effect. This is not convincing and perhaps the approach to look at whole brain activity is overly simplistic? At the least this should be discussed in the relevant result section.

We find it incredible that stimulation of the morning cells can change activity of the entire brain. This has implications for a variety of physiological functions including arousal, as well as potential pathological consequences such as seizures. We are very interested in the specific downstream brain regions that are driving this whole-brain response as well as the kinetics of that activation. A detailed anatomical and temporal map of downstream activation could be possible with BIFROST mapping (PMID: 39541350), but this is outside the scope of the present study.

As recommended by the Reviewer, we now review these points in the revised Discussion: "A limitation of our *in vivo* two-photon experiments was that upon activation of *Pdf*+ neurons, we assessed whole brain activity instead of activity of specific brain regions. While it is remarkable that activation of *Pdf*+ neurons in the morning can increase brain-wide activity, future studies should attempt to construct more specific anatomical and temporal maps of activation using standardization techniques, including BIFROST mapping[62]."

Text and data relating to Fig.7:
Again, there is deviation in the timing of seizures in the wild type shown in panel A/C compared to that shown in Fig.1 and elsewhere. This further underlines the criticism of definitive evidence to show seizure occurrence is 'tightly' regulated by the clock. Perhaps if the tone of the manuscript was altered to 'indicative of influence' these biological variations would be more acceptable.

We appreciate the Reviewer's feedback and have made an effort to acknowledge that while seizures peak during the daytime, the timing of the daytime peak can vary. We also have tempered the tone of the manuscript as suggested, including changing of the title. We do not use the word 'tightly' and make an effort to ensure that the tone does not suggest the same.

These points are reviewed in more detail above in response to the Reviewer's introductory comments in sections (c) and (d).

Text and data relating to Fig.8:
The conclusion that morning cells act on evening cells via glycine release is not supported by this data. Sure, it is indicative, but no more.

As suggested by the Reviewer, we have tempered the tone of these conclusions. The Results section has been modified to "This resembled the result obtained with glycine knockdown in morning cells and indicates that glycine from morning cells may act on glycine receptors on evening cells at night to decrease seizure risk. As the night progresses, morning cell activity ramps up[43] and direct synaptic contact with evening cells increases[41,44,45]. Therefore, our data suggest that during this ramping up of morning cell activity through the night, glycine can act on evening cells to suppress nighttime seizures."

As noted above in response to the introductory comments, the Abstract has been modified to "At night, morning cells switch to glycine signaling likely acting on evening cells to decrease seizure risk.".

Text and data relating to supp Fig.8:
Stimulation of the GF is known to induce seizure like activity. Thus, the fact that hyperactivation of this system increased seizure duration is perhaps not surprising. Moreover, as the GFS is one of the most significant brain to thoracic conduits, the effect of silencing this fibre is also not surprising. However, neither observation speaks to regulation by the clock. I do not feel that this section adds much to the study.

We agree that the role of the GF in seizures is known, but the time-of-day effect we note here supports the rhythmicity of seizure occurrence. We find that inhibition of giant fiber neurons in a relatively narrow time window from ZT0-4 (~16% of the day) reduced the average daily seizure number by ~50% and improved survival after 96 hours from 25% to 75%. These results are consistent with our hypothesis that seizure vulnerability is increased during the daytime, and important from a therapeutic perspective, inhibition of giant fiber neurons during this narrow time window dramatically improves seizure burden and survival.

To better emphasize these points, the Results have been modified as follows: "Inhibition of giant fiber neurons with GtCR1 during this narrow time window using the R68A06-Gal4 driver led to a ~50% reduction in the daily number of seizures (Supp. Fig. 12E-F). Interesting from a therapeutic perspective, this inhibition of seizures led to improved survival from 25% to 75% at 96 hours (Supp. Fig. 12G). Together, these data suggest descending motor neurons that mediate seizures are downstream of clock inputs, and that inhibition of downstream motor pathways during relatively narrow windows can be used to decrease seizures and improve survival."

Discussion: Very much a rehash of the results and could easily be shortened.

We thank the Reviewer for the constructive feedback. We have reduced repetition of the results and cut ~400 words from the previous Discussion.

General points:
The term 'spontaneous seizure' is used throughout the manuscript when in fact the seizures are induced by exposure to picrotoxin. Terminology to reflect this should be used. Indeed, a significant drawback to using flies is that they do not show truly spontaneous seizures (the few reports that suggest otherwise report only very modest seizure activity).

Since initial review of this manuscript, we published a study examining how sleep loss increases seizure risk (PMID: 40730814). There, we demonstrated that chronic video tracking can be used to identify spontaneous seizures in flies. The spontaneous events we classify as seizures (1)

match the classical movement patterns of previously described *Drosophila* seizures, (2) do not occur in wild-type flies, (3) occur concurrently with hypersynchronous brain activity, (4) worsen with sleep loss, as seen in people and mammalian models, and (5) resolve with conventional anti-seizure medications. These data provide robust evidence that the sustained hyperkinetic events we detect with continuous video tracking are indeed seizures.

To clarify how we use the word spontaneous in the current manuscript, we added the following to the Results section: "We refer to the seizures induced by picrotoxin feeding as "spontaneous" seizures, as no acute stimulus is delivered to precipitate a seizure (in contrast to "bang-sensitive" or temperature-sensitive seizures described below)."

The introduction needs to go deeper into the literature reporting clock effects on seizure occurrence in humans. The field is confused, and no clear conclusions can be drawn. This does not come through on reading the intro.

As noted above, we now include the following in the Introduction: "While more than 80% of people with epilepsy exhibit diurnal oscillations in seizure risk, the exact time of day that seizure risk peaks varies from person to person and relates to underlying genetic and structural etiologies[2–5]. Across all etiologies, the peak time for seizure occurrence on average is around the early- to mid-day, though across individuals, seizure peaks can occur at all times of the day[1,4,6] . Interictal epileptiform discharges also exhibit diurnal rhythms as revealed by prolonged recordings from implanted brain stimulation devices[2]. The phase of peak seizure timing is frequently tied to underlying brain regions involved, as well as genetic etiologies[5,6]."

We agree with the Reviewer that the field is currently confused and rationalize that the fly model can provide insights with the following paragraph in the Introduction: "Identification of the molecular and cellular mechanisms that drive these diurnal oscillations in seizure susceptibility has been challenging, because there are multiple processes that oscillate over a 24-hour period, driven by the circadian clock or by environmental cycles. Dissecting the contribution of these different processes to seizure rhythms in people[7,8], or even rodent models[7–10], has been complex. Therefore, we sought to identify underlying mechanisms in *Drosophila*, as flies are an experimentally tractable model organism exhibiting well characterized biological rhythms."

Rose plots in some figures have too many colors (and pale colors at that) and are impossible to interpret.

We appreciate the Reviewer's feedback and note that Reviewer #3 also provided similar constructive feedback. To simplify our data representation, we have completely removed these rose plots as the additional information provided was marginal.

What does FFT stand for (Fig 2E)?

This is now provided in the Fig. 2 figure legend as "Fast Fourier Transform (FFT) calculations".

XY plots in Fig 3 are not well explained

We have now explained this in the Fig. 3 figure legend: "In the *xy* plots, the *x* is calculated by subtracting the Gal4 genetic control average seizure duration from the experimental group. The *y* is calculated by subtracting the UAS genetic control average seizure duration from the

experimental group. Groups that are significantly different from the corresponding genetic control are labeled in *red* with an asterisk."

Methods state adult male flies were used, but in some expts (eg Fig 6) females are used. Why was this? Also, circadian influences on seizure might be expected to be similar in both sexes, but this has not been tested?

We, like others, use female flies for in vivo two-photon imaging because they are larger and easier to secure to the stage. The following has been added in the Methods section under two-photon imaging: "Female flies were used as they are larger and more amenable to microdissection."

We have not compared the effects of the molecular clock on seizures between sexes.

Reviewer #3 (Remarks to the Author):

The manuscript employs a video-tracking method to investigate the timing susceptibility of induced seizures in Drosophila using genetics and pharmacological models of epilepsy. The work builds upon an extensive body of literature in humans and mammals demonstrating that seizures do not occur randomly throughout the day but follow a circadian rhythm and aims to create a genetically amenable model to study this phenomenon. The hypothesis is clear, and the manuscript is potentially interesting and impactful. However, there are some major issues with data interpretation and presentation that need to be addressed.

Figure 1A and Main Text: There is a critical issue regarding the algorithm, as it is neither explained nor validated within the paper. It is only referenced in a 2023 preprint that also does not adequately explain or validate the algorithm. This work relies heavily on this algorithm, making the methodology central and critical meaning it must be validated by demonstrating that it can detect seizures at least as reproducibly and reliably as the existing state of the art.

We understand the Reviewer's valid concerns. When this manuscript was under review, another manuscript detailing our video tracking methodology was also under review. We are happy to share the other manuscript has now been published (PMID: 40730814).

We have now published detailed step-by-step instructions on the use of our seizure detection algorithm from fly video tracking data. Specifically, we provide (1) all required MATLAB code, (2) a detailed pdf with step-by-step instructions including screenshots, (3) a sample dataset, (4) sample analysis files, and (5) sample outputs. Sample screenshots from our step-by-step directions are included below:

Overview

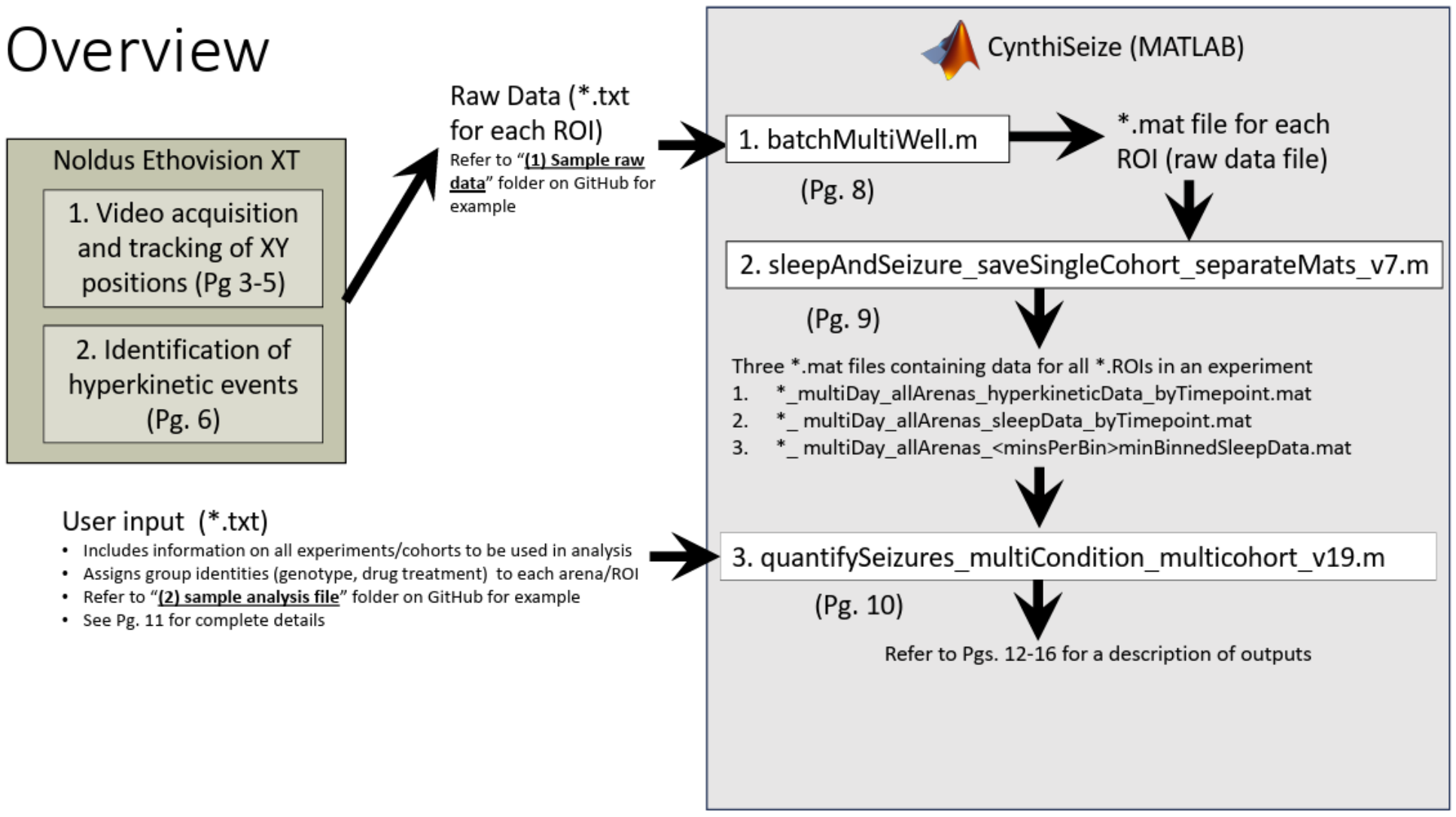

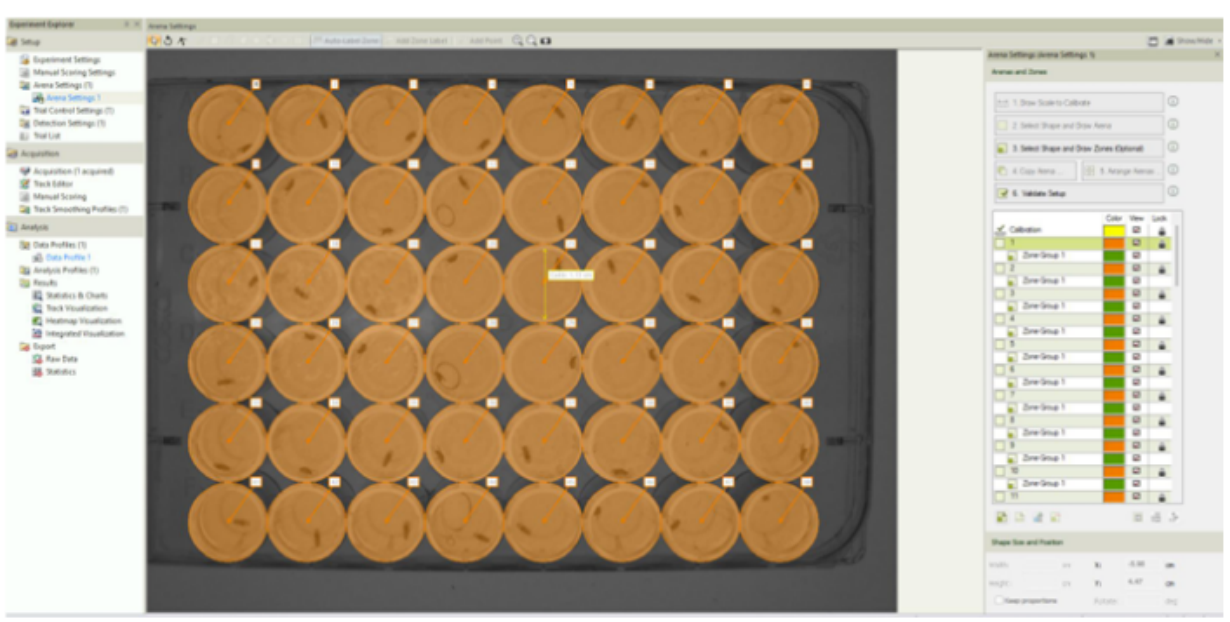

- <u>"Arena Settings"</u>
  - Here, a region of interest (ROI) is created for each well containing 1 fly
  - Well diameter is set (e.g. 1.13 cm for a 48-well plate)

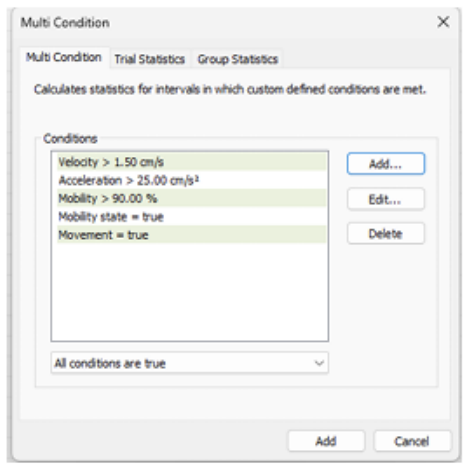

Identification of Hyperkinetic Events

- "Analysis profile"
  - Hyperkinetic events are then identified as defined by the metrics shown
    - Velocity > 1.5 cm/sec
    - Acceleration > 25 cm/sec^2
    - Mobility > 90%
    - Mobility state = true
      - Highly mobile, with highly mobile thresholds at 80% and immobility below 30%
    - Movement = true
      - Moving of the center-point, with thresholds at 1.5 and 1.0 cm/sec
  - "Trial Statistics" and "Raw Data" are then exported for further analysis in MATLAB
    - Export data as an ANSI text file with semicolon delimiters.
    - Sample outputs (a separate *.txt file is generated for each ROI/arena):
      - Track-20240301 picro, LEV, VPA VT2-Trial 1-1-Subject 1.txt
      - Track-20240301 picro, LEV, VPA VT2-Trial 1-1-Subject 34.txt

All of this has been deposited on Zenodo, an open-access repository with a persistent Digital Object Identifier (DOI). This is all publicly accessible at https://zenodo.org/records/15619740.

In this other manuscript, we also provide validation that video-tracking can detect spontaneous seizures in *Drosophila* in an automated manner. We demonstrated that these paroxysmal events of hyperkinetic movement are indeed seizures because (1) the tonic clonic movements match those previously ascribed to classical bang-sensitive seizures, (2) these movements do not occur in wild type flies on typical media in light:dark conditions, (3) these hyperkinetic events are associated with a surge in brain activity as measured by a calcium-dependent luciferase activity, (4) there is a worsening with poor sleep as seen in rodent models and people with epilepsy, and (5) these hyperkinetic events resolve with clinically-relevant anti-seizure medications.

Figure 1B: The Y-axis is not annotated in this figure nor in other figures that use the same visualization techniques. This omission makes comparisons across conditions simply impossible.

We sincerely appreciate the Reviewer's constructive feedback and have improved our data representation throughout the manuscript to allow for comparisons between experiments. In the former version, we depicted two major pieces of data with this visualization technique: the daily timing of seizures and the number of seizures. However, the number of seizures was also more clearly quantified and compared in our bar plots depicting "Daily number of seizures". Because this may be perceived as repetitive, we now use the 24-hour plots of seizures to focus exclusively on depicting seizure timing and maintain the bar plots to depict seizure numbers. In these revised plots, all seizures within an experiment are normalized to the seizure peak, allowing for comparisons of seizure timing across experiments. The bar plots continue to allow for comparison of seizure numbers across experiments.

Picrotoxin-Related Issues in Figure 1 and Other Figures: This is the main issue with this study. Picrotoxin induces seizures in Drosophila larvae within minutes of exposure. If I understand correctly, the flies are kept on this substrate for the duration of the experiment, meaning that seizures could occur after every feeding episode, which has a circadian component by itself. If that is the case, it is unsurprising that seizure probability is higher during the day than at night

and is affected in circadian mutants. This represents a major weakness, as it could invalidate all experiments based on feeding. Feeding itself should be monitored, and the risk of seizures should be controlled and normalized over feeding patterns. Notably, the authors report that “No significant differences in the likelihood of seizure induction were noted in tko25t flies throughout the day (Supp. Fig. 1D)” and that “Again, the likelihood of seizure induction across groups did not change throughout the day (Supp. Fig. 1G)”, indicating that the circadian signal observed with picrotoxin feeding disappears when other proconvulsant models are used. This is one more reason why feeding control is absolutely crucial.

We thank the Reviewer for their feedback and have performed additional experiments to demonstrate that feeding does not drive the picrotoxin-induced diurnal oscillations in seizure risk we observe. Below, we highlight (1) in light:dark cycles, clock mutants, including the *tim^{01}* mutant we use here, exhibit intact diurnal rhythms of locomotor activity, (2) in light:dark cycles, clock mutants, including the *tim^{01}* mutant we use here, exhibit intact diurnal feeding rhythms, (3) in additional experiments, we tested daytime and evening feeding and find that *tim^{01}* mutants and wild-type flies feed similarly, (4) as compared to larvae, we do not observe seizures immediately after feeding; thus seizure risk is not directly tied to feeding bouts as observed in larvae, (5) when picrotoxin feeding was shifted by 12 hours, daytime seizures still predominated, and (6) that three seizure-prone lines exhibit diurnal oscillations in seizure severity independent of feeding. Thus, strong evidence indicates that the diurnal oscillations in seizure risk we describe here are not driven by diurnal feeding rhythms.

(1) In light:dark cycles, clock mutants exhibit largely intact behavioral rhythms. In our video-tracking experiments presented here, we would like to emphasize that the experiments are conducted in a 12-hour light:12-hour dark cycle, unless otherwise indicated (e.g. Supp. Fig. 2). In light:dark cycles, *tim^{01}* mutant flies, as well as other clock mutants including *per^{0}*, continue to exhibit diurnal rhythms in activity (Sehgal et al, *Science*, 1994; Helfrich and Engelmann, *Z Naturforsch C J Biosci.* 1987). Thus, the lack of seizure rhythms in clock mutants we observe cannot be explained by rhythms in activity.
This is now highlighted in the Results as “When *timeless* (*tim^{01}*) mutant flies, which lack a functional molecular clock but still exhibit diurnal patterns of activity in a light:dark cycle[15], were fed picrotoxin, seizures were randomly distributed throughout the day and did not show evidence of differing from a uniform distribution (Fig. 1B-D; pRayleigh >0.05).”

(2) In light:dark cycles, multiple laboratories have shown that clock mutants, including *tim^{01}* mutants, have intact diurnal feeding rhythms similar to wild-type flies. We use light:dark cycles in our video-tracking experiments (unless otherwise indicated). Preserved diurnal feeding rhythms in light:dark cycles have been demonstrated by multiple groups specifically for the *tim^{01}* mutants we use here across multiple modalities including Capillary Feeder Assay and ingestion of radioactive food (Seay and Thummel, *J Biological Rhythms*, 2011 – see Fig. 1B and related text) (Maruko et al, *iScience*, 2023 – see Fig 2A and related text). Rhythmic feeding in light:dark is also observed in *Clk^{jrk}* mutants (Xu et al, *Cell Metabolism*, 2008). These circadian rhythms of feeding in clock mutants are lost in complete darkness, as expected of a circadian process.

(3) Consistent with previous studies, we find that wild-type flies and *tim^{01}* mutants consume similar amounts of food in light and dark. As recommended by the Reviewer, we

examined feeding in wild-type and *tim*^{01} mutants using a Capillary Feeder Assay in light:dark conditions. We find that wild-type and *tim*^{01} mutant flies eat similar amounts of food in the morning and evening, in agreement with previous studies. Wild-type flies continue to consume food through the evening at levels similar to *tim*^{01} flies, but very few seizures occur at night in wild-type flies. Thus, feeding does not drive the seizure rhythm. These new data are below and in the manuscript as Supp. Fig. 3.

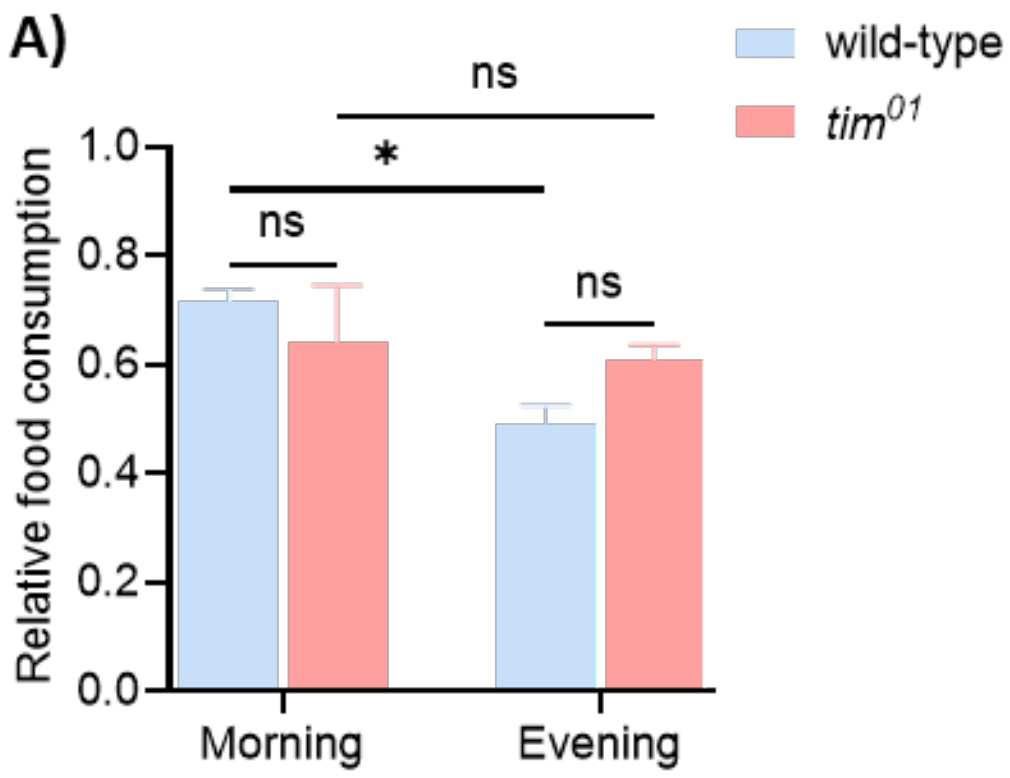

This is summarized in the Results as "Also, differences are not due to altered feeding as *tim*^{01} mutants and other clock mutants show rhythmic feeding in a light:dark cycle[16–18]. We find too that food intake is similar in wild-type flies and *tim*^{01} mutants at two different times of day (Supp. Fig. 3A)."

(4) Picrotoxin-induced seizures in adult flies do not occur immediately after feeding. The Reviewer points out that seizures are induced in larvae minutes after picrotoxin feeding.

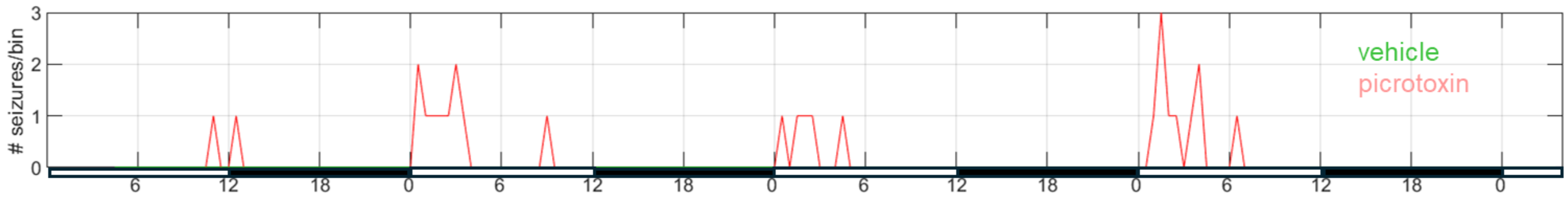

In contrast in adult flies, we find that seizures are not directly tied to feeding bouts. In the experiments from Supp. Fig. 2A show below, flies are placed on picrotoxin at ZT0-4. When the data are plotted across consecutive day:night cycles as shown below, the first seizure does not occur until ZT11.

This is consistent with previous findings demonstrating that the induction of seizures in adult flies is inversely proportional to the dose administered and ranging from several hours to ~24 hours (Stilwell et al, *Eur J Neurosci*, 2006). Thus, in adult flies, there is not a 1:1 relationship between picrotoxin feeding and seizures at the concentrations we use (0.25-0.5 mg/ml).

(5) When picrotoxin feeding is shifted by 12 hours, daytime seizures still predominate. We also show that when feeding is preferentially started in the nighttime (ZT12), daytime seizures persist (Supp. Fig. 1C). This is important given that flies do continue to eat throughout the night, albeit less than the daytime.

(6) We identified diurnal oscillations in seizure severity in mutants that do not rely on seizure induction through feeding. We identified diurnal seizure rhythms in the 3 mutant lines tested here (tko^{25t}, $para^{DS}$, and $para^{GEFS+}$), which exhibit seizures independent of feeding. tko^{25t} is a bang-sensitive model of epilepsy and relies on a strong mechanical stimulus to induce seizures in nearly all flies tested. Therefore, we are not able to use the likelihood of seizure induction as a proxy for seizure risk but instead use seizure duration as a measure of seizure severity. Thus, while we report the percentage of tko^{25t} flies having a seizure for the sake of completeness (Supp. Fig. 1D), the circadian rhythm for this bang-sensitive mutant is actually in seizure duration (Fig. 2B). This measure of "seizure duration" or "time to recovery" as a measure of seizure severity for bang-sensitive flies is widely used in the literature, e.g. Richard Baines' group (Mituzaite et al, *eNeuro*, 2021) and Hugo Bellen's group (Lu et al, *AJHG*, 2022; Bereshneh et al, *AJHG*, 2025). In addition, we identify diurnal rhythms in temperature-sensitive seizures in $para^{DS}$ and $para^{GEFS+}$ mutant flies in a new Supp. Fig. 4.

Fig1 D and conclusion 335-337: The observed deaths predominantly occurred in the early evening. While this finding is interesting, attributing it to sleep pressure (L335) is certainly premature.

We agree with the Reviewer that tying lethal seizures occurring in the early evening to sleep pressure is entirely speculative. We have edited the Discussion as follows to make it clearer that further work needs to test this possibility: "The increased risk of lethal seizures at night in clock mutant flies demonstrates that this process is clock-independent; though speculative at this point, one possibility is that lethal seizures in the early evening are driven by sleep need. At ZT12, when the lights are switched from on to off, flies have a high sleep need and fall asleep. Thus, the high lethality at night could be tied to (a) the sleep state, (b) a transition from wake to sleep, or (c) a high sleep need. Therefore, further studies examining susceptibility to lethal seizures during sleep-wake transitions or during times of high sleep need are warranted."

Figure 2A: Additional details should be provided on how seizure quality was calculated. Line 106 states that the seizures are video recorded, but it is unclear whether the quantification is performed manually or by the algorithm.

We now clarify that the quantification of duration of seizure phases is done manually: "Seizures were induced at 6 timepoints through the day (ZT0, ZT4, ZT8, ZT12, ZT16, and ZT20 where "ZT" is zeitgeber time) and video recorded to allow for manual quantification of each phase of the seizure (Fig. 2A, Supp. Video 1)."

In addition, more detail regarding manual quantification of bang-sensitive seizures is provided in the Methods section: "As previously described [6], tko^{25t} bang-sensitive mutants were placed in groups of 4 flies into vials containing agar (2%) (Fisher NC1429200) and sucrose (5%). Vials were struck on a countertop four times and then vortexed on a Fisher Vortex Genie 2 (#12-812) at setting '5' for 5 seconds. Vials were then placed in front of a camera for video recording on a PC using OBS Studio 29.0. After the experiment, videos were manually scored for percentage flies with seizure in each vial, duration in atonic ("paralysis") phase, duration in tonic/clonic ("convulsive") phase, and duration in postictal ("recovery") phase for each individual fly. Scorers were blinded when possible. To achieve the multiple timepoints tested, separate sets of flies were entrained in incubators on different light-dark cycles."

Figure 2E/D/G: What are those yellow boxes? They are not labeled in the figure, legend, or text. Additionally, this figure would be completely cryptic to a reader who is not already extremely familiar with the model and the genotypes.

We regret this oversight. The figure legend has been corrected: “d,g, Representative activity records in which vertical lines indicate activity. Rows represent successive days over which activity was monitored. Yellow shading indicates light for a 12-hour light:12-hour dark period. e,h, Fast Fourier Transform (FFT) calculations. n=27-48 flies/condition. One-way ANOVA with Tukey’s multiple comparisons test was used.”

Figure 3: The same data are essentially presented in two different ways: with bar plots on the left and a different system on the right. Choose only one method, as there is no need to show the same information twice. The graph on the right is appealing but does not convey information about standard error and statistical comparisons in general. The legend states: “For unknown reasons, in tim-Gal4;tko25t flies, only 1 out of 96 flies tested exhibited a seizure so significance testing is not performed for this group.” This finding seems quite relevant and striking but is not discussed in the paper.

We have taken the Reviewer’s constructive feedback to improve our data visualization. We have removed the rose plots as the additional information conveyed is marginal as the Reviewer indicates. In addition, in response to reviewer feedback, we have normalized all 24-hour plots of seizure timing for each experiment to allow for comparison of seizure timing across experiments (e.g. Fig. 1B). Information about seizure numbers is still preserved and is quantified in our plots of “Daily number of seizures” (e.g. Fig. 1C). In making these suggested modifications, we agree there is less repetition of the data. This also allows us to incorporate feedback from Reviewer #2 regarding the lack of clarity of rose plots when multiple conditions are overlaid.

We are uncertain why the *tim*-Gal4;*tko*^{25t} flies exhibited so few seizures. While this Gal4 screen was performed on an isogenic background, it is possible a genetic variant segregating with *tim*-Gal4 on chromosome II has a seizure-suppressive effect. Due to the speculative nature of this possibility, we simply state this occurred for unknown reasons.

Fig. 7 and Supplementary Fig. 6: The authors show PDF and sNPF change seizures, while sleep is not affected. However, these findings may be inconsistent with previously studies. For instance, Chung et al Fig. 1C (https://www.sciencedirect.com/science/article/pii/S0960982209006125?via=ihub) reported that pdfrhan5304 showed increased sleep, and Shang et al Fig. 4A (https://www.sciencedirect.com/science/article/pii/S0896627313006533?via=ihub) found that knockdown of sNPF in pdf-Gal4 neurons decreased night sleep.

All of our experiments involving seizure, sleep, and survival quantification for Figure 7 and the related supplementary figures are in the presence of picrotoxin. In Supp. Fig 1A, we show that picrotoxin itself can change sleep amount (by increasing daily sleep). The presence of picrotoxin may account for the differences between our study and the previously published ones.

We added the following note to the Results section: “We note that prior studies have demonstrated changes in sleep amount in *pdfr*^{han5304} mutants and after sNPF knockdown[43,44], but in the presence of picrotoxin, we did not see significant differences.”

Figures 4/5/7/8: These figures suffer from the same issues as Figure 1 in terms of data presentation and the inability to draw conclusions related to picrotoxin feeding. May afraid it is simply premature to draw any conclusion until that major caveat is taken into account.

We have made the changes suggested by the Reviewer regarding data presentation. As noted above, multiple lines of evidence indicate the rhythm in picrotoxin-induced seizures is not driven by rhythms in feeding.

Reviewer #4 (Remarks to the Author):

I co-reviewed this manuscript with one of the reviewers who provided the listed reports. This is part of the Nature Communications initiative to facilitate training in peer review and to provide appropriate recognition for Early Career Researchers who co-review manuscripts.

REVIEWERS' COMMENTS

Reviewer #1 (Remarks to the Author):

The authors have thoroughly addressed all of my concerns. I have no further suggestions, and I congratulate them on a fine piece of work.

We thank the Reviewer for their constructive feedback.

Reviewer #2 (Remarks to the Author):

Whilst the revised manuscript has been improved, there are still some areas that require further elaboration/amending. These are:

1. Comparison of the effect of WT+PTX between tim(01) and LL shows obvious differences. The former shows no circadian influence (as expected) but the latter seemingly does (Raleigh P<0.001 which is similar to the WT, other than peak seizure count is shifted towards subjective night). Given that both conditions are expected to have a non-functional clock, why the difference?

We thank the reviewer for the comparison between methods of clock disruption and have clarified the distinction. Constant light (LL) and tim^{01} mutant flies exhibit disrupted molecular clocks in fundamentally different ways.

tim^{01} mutant flies lack a core circadian oscillator constitutively. Accordingly, we see that picrotoxin-induced seizures in tim^{01} mutants show no circadian oscillation (Fig. 1B). In contrast, LL disrupts the clock indirectly and progressively via CRY-mediated TIM degradation. The effects of LL on rhythms are not immediate and may not completely block molecular clock function. Our own previous work demonstrates that wild-type flies require 1-2 days of LL exposure before becoming arrhythmic (PMID: 16794082). The data from our LL experiments are presented from the time LL starts; flies were not pre-exposed to LL before seizure recording. Therefore, some residual and progressively dampening molecular clock function is expected. We therefore interpret the significant Rayleigh statistic under LL (Supp. Fig. 2C) not as evidence of an intact clock, but as the consequence of LL being a slower and less complete clock-disrupting manipulation as compared to tim^{01} mutant flies.

In addition, LL is toxic, and picrotoxin-treated flies in LL sleep less. LL elevates reactive oxygen species, lipid peroxidation, and mitochondrial dysfunction (PMID: 29655867). We found that LL significantly increased seizure number (Supp. Fig. 2D) and even induced a seizure without picrotoxin (Supp. Fig. 2C, *upper*). Picrotoxin-treated flies in LL sleep less (Supp. Fig. 2E), and we have previously demonstrated that elevated sleep pressure independently increases seizure risk (PMID: 40730814). This could account for the peak referred to by the reviewer.

We have further clarified this in the Results: "In constant light, diurnal differences in seizures were dampened, and there was a shift in phase to a nighttime peak in seizures. Because constant light disrupts the clock less more slowly (PMID: 16794082) and less completely than a tim^{01} null mutant, residual circadian organization of seizures is not surprising. In addition, flies in constant light exposed to picrotoxin sleep less (Supp. Fig. 2E), which independently increases seizure risk[13]. Surprisingly in constant light, we detected a seizure in a wild-type fly and a six-fold increase in picrotoxin-induced seizures, reflecting previously-described toxicity of constant light exposure[19] (Supp. Fig. 2C-D; Supp. Video 2)."

2. The first discussion paragraph identifies 4 key findings of this study. However, point 1 is not

novel. Other studies have shown a circadian influence on seizure in Drosophila, (https://pubmed.ncbi.nlm.nih.gov/41958013/). It is appropriate that previous studies are cited.

We have added the following to the Discussion: "While this manuscript was under review, Huertas-Radi et al. reported clock-dependent rhythms in the severity of mechanically induced seizures in bang-sensitive mutants[55], consistent with our finding that seizure timing is under circadian control."

3. Whilst the authors make a strong case to maintain the section on the Giant Fiber, their conclusion that the results on this circuit have 'therapeutic potential' is questionable. Indeed, the penultimate paragraph of the Discussion points out the many limitations of using the fly for human epilepsy. The mention of 'therapeutic potential' should either be fully explained or deleted.

The referenced sentence has been rephrased to "This inhibition of seizures led to improved survival from 25% to 75% at 96 hours (Supp. Fig. 12G)."

Reviewer #2 (Remarks on code availability):
outside of my expertise

Reviewer #3 (Remarks to the Author):
I thank the authors for their thorough and considered response to my original comments. The revision represents a substantial body of additional work, and I find that my concerns have been addressed convincingly. The methodological transparency around the seizure-detection pipeline is now resolved and I commend the authors for sharing the methods on Zenodo. My main concern — that the diurnal rhythm in picrotoxin-induced seizures might simply reflect rhythmic feeding — has been addressed through a multi-pronged argument that I find persuasive in aggregate.
Overall, this is a careful, technically demanding study that establishes Drosophila as a tractable system for dissecting the molecular and circuit basis of diurnal seizure rhythms. I am happy to recommend acceptance.
Giorgio Gilestro

We thank Dr. Gilestro for his valuable feedback to improve the manuscript.

Reviewer #4 (Remarks to the Author):
I co-reviewed this manuscript with one of the reviewers who provided the listed reports. This is part of the Nature Communications initiative to facilitate training in peer review and to provide appropriate recognition for Early Career Researchers who co-review manuscripts.

Reviewer #4 (Remarks on code availability):
The authors have deposited a comprehensive "CynthiSeize" package on Zenodo. This includes a detailed PDF with step-by-step instructions and screenshots for every stage of the pipeline, from video acquisition in Ethovision XT to final data quantification in MATLAB.

We thank the reviewer for their constructive feedback.
